# Over 400 food resources from Brazil: evidence-based records of wild edible mushrooms

**DOI:** 10.1186/s43008-024-00171-8

**Published:** 2024-12-13

**Authors:** Mariana P. Drewinski, Marina Pires Corrêa-Santos, Vitor X. Lima, Felipe T. Lima, Melissa Palacio, Maria Eduarda A. Borges, Larissa Trierveiler-Pereira, Altielys C. Magnago, Ariadne N. M. Furtado, Alexandre R. Lenz, Alexandre G. S. Silva-Filho, Cristiano C. Nascimento, Renato L. M. Alvarenga, Tatiana B. Gibertoni, Jadson J. S. Oliveira, Juliano M. Baltazar, Maria Alice Neves, Ruby Vargas-Isla, Noemia K. Ishikawa, Nelson Menolli

**Affiliations:** 1 IFungiLab, Subárea de Biologia, Departamento de Ciências da Natureza E Matemática, Instituto Federal de Educação, Ciência e Tecnologia de São Paulo, Campus São Paulo, Rua Pedro Vicente 625, São Paulo, SP 01109-010 Brazil; 2 Núcleo de Pós-Graduação Stricto Sensu, Pós-Graduação em Biodiversidade Vegetal e Meio Ambiente, Instituto de Pesquisas Ambientais, Av. Miguel Stefano 3687, Água Funda, São Paulo, SP 04301-012 Brazil; 3 Centro de Biociências (CB), Departamento de Micologia, Universidade Federal de Pernambuco, Avenida da Engenharia, S/N – Cidade Universitária, Recife, PE 50740-600 Brazil; 4 Laboratório de Micologia, Departamento de Botânica, Instituto de Biociências, Universidade Federal Do Rio Grande Do Sul, Av. Bento Gonçalves 9500, Prédio 43.433, Campus Do Vale, Agronomia, Porto Alegre, RS 91501-970 Brazil; 5 Algas e Plantas, Laboratório de Micologia (MICOLAB-UFSC), Departamento de Botânica, Centro de Ciências Biológicas, Programa de Pós-Graduação em Biologia de Fungos, Universidade Federal de Santa Catarina, Campus Universitário Reitor João David Ferreira Lima, S/nº, Florianópolis, SC 88040-900 Brazil; 6 Laboratório de Estudos Micológicos (LEMic-UFSCar), Centro de Ciências da Natureza, Universidade Federal de São Carlos, Campus Lagoa Do Sino, Buri, SP Brazil; 7 Departamento de Botânica, Universidade Federal do Espírito Santo (UFES), Av. Fernando Ferrari, 514, Vitória, ES 29075-910 Brazil; 8 Laboratório de Genética Evolutiva Paulo Leminski, Departamento de Biologia Molecular, CCEN, Universidade Federal da Paraíba, Cidade Universitária, João Pessoa, PB 58051-900 Brazil; 9 Grupo de Pesquisa em Bioinformática e Biologia Computacional (G2BC), Departamento de Ciências Exatas e da Terra, Universidade do Estado da Bahia, Campus I, Salvador, BA Brazil; 10 Divisão do Curso de Pós-Graduação em Botânica, Coordenação de Biodiversidade, Instituto Nacional de Pesquisas da Amazônia, Av. André Araújo 2936, Manaus, AM 69067-375 Brazil; 11 Grupo de Pesquisa Cogumelos da Amazônia, Coordenação de Biodiversidade (COBIO), Instituto Nacional de Pesquisas da Amazônia (INPA), Av. André Araújo, 2936, Manaus, AM 69067-375 Brazil

**Keywords:** Biodiversity, Brazilian edible mushrooms, Funga, Phylogeny, Species list, Wild edible fungi

## Abstract

Many species of mushroom-forming fungi have been harvested in the wild and used for food and medicine for thousands of years. In Brazil, the knowledge of the diversity of wild edible mushrooms remains scattered and poorly studied. Based on new samples, bibliographic records revision, and searches through the GenBank, we recorded 409 species of wild edible mushrooms in Brazil, of which 350 can be safely consumed and 59 are edible but with conditions. Additionally, other 150 species represent taxa with unclear evidence of consumption or unconfirmed edibility status. A total of 86 of the 409 edible species represents consistent records in Brazil based on molecular data and/or Brazilian nomenclatural types. Other 323 names represent species that need further taxonomic investigations to confirm their identity and occurrence in the country, with 41 of them having some record of consumption by part of the Brazilian population. The remaining 282 species can represent new food resources for the country. We generated 143 DNA sequences, representing 40 species within 29 genera. Edible mushrooms are an important non-wood forest product and the knowledge about them adds value to the local biodiversity and the population, increasing the incentive to conservation allied to sustainable rural development.

**Supplementary Information:**

The online version contains supplementary material available at 10.1186/s43008-024-00171-8.

## INTRODUCTION

Wild edible mushrooms (WEM)^1^ have been harvested and used by people for food and medicine in more than 90 countries for thousands of years (Li et al. 2021). The oldest evidence of fungi consumption by humans is based on food debris from dental calculus samples from Neanderthals, who became extinct around 40,000 years ago (Higham et al. 2014; Weyrich et al. 2017), and Magdalenian individuals who lived around 18,700 years ago in El Mirón Cave, Spain (Morales and Straus 2015; Power et al. 2015).

Considering a best estimation of the diversity of fungi (about 2.5 million species; Niskanen et al. 2023) and the proportion of 18.75% mushroom-forming fungi (Hawksworth 2001), the estimated number of macrofungi species is about between 469,000 species. Despite the magnitude of the numbers, the real diversity is poorly known. Currently, about 148,000 species of fungi are recognized (Antonelli et al. 2020) of which 27,750 are mushrooms if we consider the proportion estimated by Hawksworth (2001).

Traditional knowledge remains an important source of recognition of the edibility of wild fungi (Boa 2004). Recently, Li et al. (2021) published a review of the world’s edible mushroom species and proposed a system for categorizing species in a final edible status. The authors recorded 2,189 edible species, of which 2,006 can be safely consumed and 183 require some preparation or have been associated with allergic reactions.

The use of wild mushrooms by contemporary human populations varies different geographic regions, from the long and notable traditional use in China (Wu et al. 2019) and Mexico (López-García et al. 2020; Pérez-Moreno et al. 2020) to more restricted consumption by the indigenous people in South America (Pérez-Moreno et al. 2021a). In Brazil, Fidalgo (1965) was a pioneer in ethnomycological studies, recording that Brazilian indigenous people from the Amazon region recognize fungi and differentiate them from plants and animals, and sometimes designating them as food or medicine.

Sir Ghillean T. Prance, a British botanist, also made a huge contribution to ethnomycological study in Brazil (Prance 1972, 1973, 1984, 1986; Fidalgo and Prance 1976). Prance (1972) conducted an ethnobotanical comparison between four indigenous Amazonian communities during a collecting expedition in 1971, in which the use of four edible fungi was recorded. In Prance’s study, the Waikás (Yanomami ethnic group) were the only community observed using fungi as part of their diet, but he found that the Sanöma group (also Yanomami) recognized and ate many mushrooms. Oswaldo Fidalgo and G.T. Prance returned to the Sanöma village in 1974 and recorded 21 species of WEM consumed by this Yanomami group, most of them collected from cassava plantations (Fidalgo and Prance 1976). The authors reported that due to lack of fishing and hunting, the Sanöma used caterpillars, larvae, and fungi to provide protein in their diet (Fidalgo and Prance 1976).

Another important ethnomycological study from Brazil was carried out with the Caiabi, Txicão, and Txucarramãe groups in the Xingu Indigenous Park, in the state of Mato Grosso, in the southern part of the Brazilian Amazon Forest (Fidalgo and Hirata 1979). In this study, 26 indigenous mycological terms have been mentioned and discussed. For the Caiabi group, most of the red or brown mushroom species are considered inedible, whilst some white or black mushroom species are considered edible (Fidalgo and Hirata 1979). Among the fungi collected during the expedition in the Xingu Indigenous Park, the Caiabi mentioned a single species for medicinal use, *Pycnoporus sanguineus*, but no edible mushrooms consumed by the Caiabi group were collected at that time. The Txicão group reported the consumption of some mushrooms, two of them collected during that expedition: *Lentinus crinitus* and *Auricularia fuscosuccinea* (Fidalgo and Hirata 1979). For the Txucarramãe group, fungi are used only as a last resource, in the absence of other food (Fidalgo and Hirata 1979).

More recently, in the twenty-first century, some other works have been published reviewing previous studies and updating and systematizing the information on WEM based on ethnomycological records (Góes-Neto and Bandeira 2003; Cardoso et al. 2010; Vargas-Isla et al. 2013). According to Vargas-Isla et al. (2013), *Auricularia*, *Favolus*, *Lentinula*, *Lentinus*, *Panus*, and *Pleurotus* are the genera with edible species most reported by the indigenous and traditional groups of the Amazon region. In 2016, Sanuma et al. (2016) published a book as result of a joint effort of researchers, including non-indigenous and the Sanöma group, the Yanomami people who inhabit the Brazilian Amazon Forest. The book presented 15 WEM species used by this ethnic group, all harvested from wood because the Sanöma group does not consume species that grow on the soil (Sanuma et al. 2016).

For other regions and ethnic groups from Brazil, little is known about the consumption habits of wild mushrooms. Meijer (2001) reported the use of *Agaricus arvensis* and *Auricularia fuscosuccinea* by European and Japanese immigrants in the state of Paraná, Southern Brazil. Recently, three species were recorded as edible for the first time based on ethnomycological records from Southeastern Brazil. Trierveiler-Pereira (2019) reported the consumption of *Neofavolus subpurpurascens*, Prado-Elias et al. (2022) recorded the edibility of *Phlebopus beniensis* by rural communities in the state of São Paulo and Coelho-Nascimento et al. (2024) recorded the use of *Pseudohydnum viridimontanum*. Ishikawa et al. (2017) carried out a bibliographic survey and reported the occurrence of about 90 edible mushroom species in the state of São Paulo, but the authors only mentioned the name of 12 wild species with potential to test cultivation conditions.

Despite these aforementioned works and considering the enormous biodiversity in Brazil, the knowledge about the diversity of WEM remains scattered and poorly documented and used for food. Thus, based on bibliographical records, new sampling, and molecular identification with DNA sequences of specimens from Brazil, we aim to summarize the current knowledge about the diversity of WEM in the country and to categorize the gathered data to certify the occurrence and consumption of each species recorded.

## MATERIALS AND METHODS

### Bibliographical research

From the global list of edible and poisonous species published by Li et al. (2021), we carried out searches in the literature for the record of WEM species in Brazil. Searches for the current name and synonyms of the species were based on the ‘Flora e Funga do Brasil’ project (http://floradobrasil.jbrj.gov.br/) and on Brazilian checklists (Putzke 1994; Meijer 2001, 2006; Baltazar and Gibertoni 2009; Trierveiler-Pereira and Baseia 2009; Sá et al. 2013; Sulzbacher et al. 2013; Coimbra 2014, 2015; Alvarenga and Xavier-Santos 2015; Meiras-Ottoni et al. 2017) and macrofungal species guides (Pegler 1997; Meijer 2008; Neves et al. 2013; Sanuma et al. 2016; Putzke and Putzke 2017, 2019; Santos 2017; Timm 2018, 2021; Trierveiler-Pereira 2019, 2022). In addition, the Google Scholar (https://scholar.google.com/) search and the authors’ personal bibliographic database were also consulted. All the original literatures were checked, and the current species names, synonyms, and authorities were based primarily on the Index Fungorum database (http://www.indexfungorum.org/), unless taxonomic and identification notes were added (see Supplementary Information 1). Species records identified as affinis (aff.) were not included in the list because they do not represent the species whose edibility is known. The data recovered from the literature are compiled in the Supplementary Information 1.

## SAMPLING

**Fig. 1:**
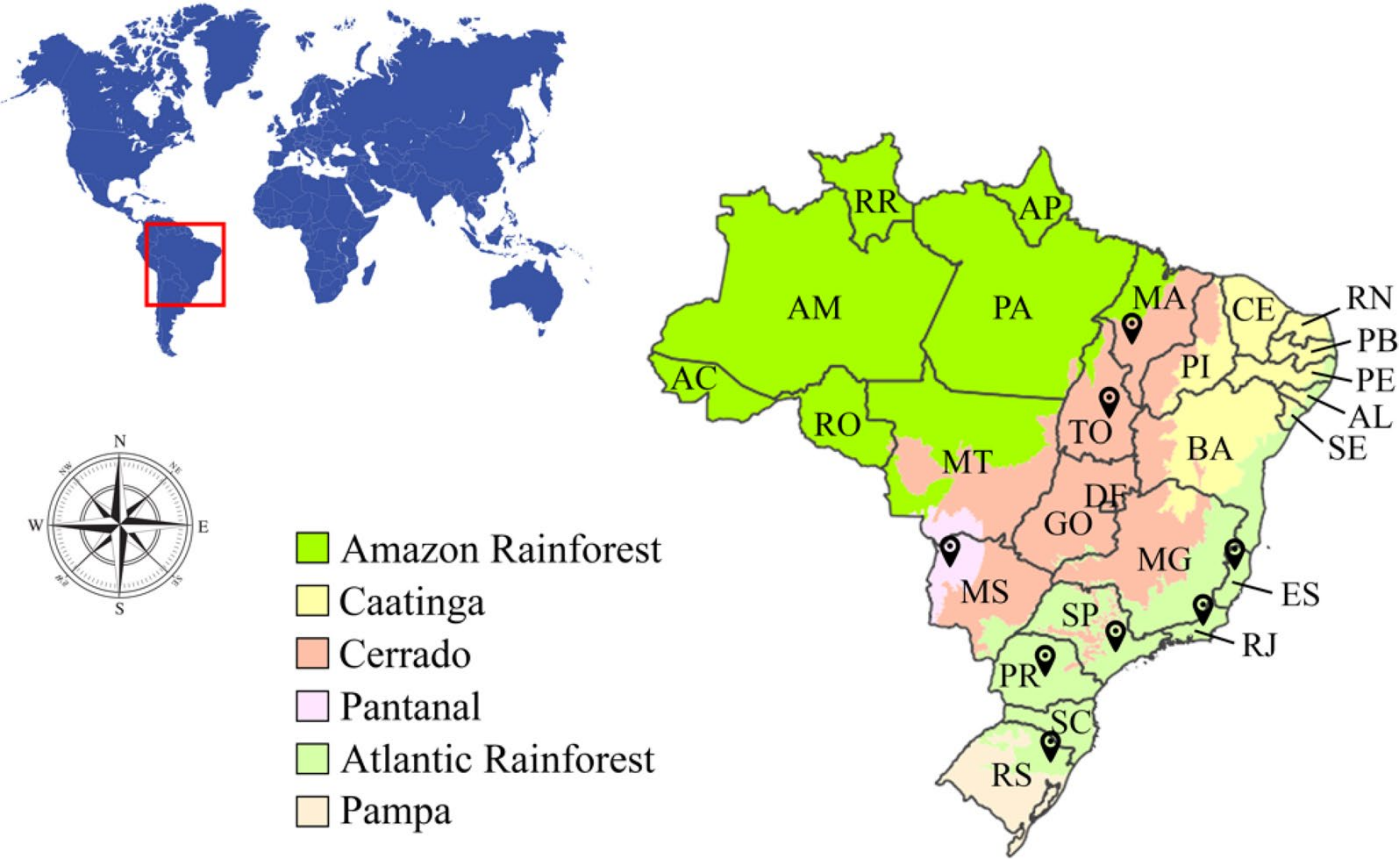
Map of the Brazilian federative units and biomes. Colored areas are the Brazilian biomes (IBGE 2019) and the black points represent the sampling sites

We carried out opportunistic collections of WEM in three different Brazilian biomes and domains from eight Brazilian states: the Atlantic Forest domain, in the states of Espírito Santo, Paraná, Rio de Janeiro, Rio Grande do Sul, and São Paulo; the Pantanal biome, in the state of Mato Grosso do Sul; and in the Cerrado biome, in the states of Maranhão and Tocantins (Fig. 1). Strain isolation was performed in the field whenever possible. For this, fragments of the mushroom context were inoculated into Petri dishes containing Potato Dextrose Agar (PDA) medium and were incubated at 25 °C until complete mycelial growth. The dried vouchers of the collected specimens are deposited at the Herbarium SP (Maria Eneyda P.K. Fidalgo), and the mycelial cultures at the ‘Coleção de Culturas de Algas, Fungos e Cianobactérias’, both at the ‘Instituto de Pesquisas Ambientais’ (São Paulo, SP, Brazil). Duplicates of the dried specimens are at the Fungarium IFungiLab (FIFUNGI) at the ‘Instituto Federal de Educação, Ciência e Tecnologia de São Paulo’ (São Paulo, SP, Brazil). This study is according to the Brazilian legislation on access to genetic biodiversity heritage and is registered in the ‘Sistema Nacional de Gestão do Patrimônio Genético e do Conhecimento Tradicional Associado’ (SisGen #A1886D5).

## MOLECULAR STUDIES

Total DNA was extracted from cultures or from small pieces of dried specimens, following a modified CTAB extraction method (Doyle and Doyle 1987). The nuc ITS1–5.8S–ITS2 (ITS) region was amplified and sequenced using the primer pair ITS1F and ITS4R (White et al. 1990; Gardes and Bruns 1993) and the nuclear ribosomal large subunit (LSU) region was amplified and sequenced using the primer pair LR0R/LR5 or LR0R/LR7 (James et al. 2006; Vilgalys and Hester 1990). The PCR reactions were carried out in 25 µl volume reaction and the thermal profile was according to Oliveira et al. (2014) or Binder and Hibbett (2003). The amplified products were purified with QIAquick PCR Purification Kit and sequenced at MacroGen (South Korea). The generated sequences were manually reviewed and edited with Geneious v.8.1 (Kearse et al. 2012).

## AVAILABLE SEQUENCES

All newly DNA sequences generated in this work are deposited in GenBank. Additional searches were conducted in GenBank based on the metadata generated by Menolli and Sánchez-García (2020) to retrieve sequences from samples of edible mushrooms previously recorded in Brazil. All data from new and previously available sequences are compiled in the Supplementary Information 1.

## PHYLOGENETIC ANALYSES

Phylogenetic analyses were carried out to confirm the identity of the sequences recovered from the WEM from Brazil. The matrices for the analyses were built mainly by genus taxonomic rank. We used the standard nucleotide Basic Local Alignment Search Tool (BLASTn) to find similarity between the sequences of Brazil’s specimens with those available at GenBank. Alignment of each ITS sequence dataset was performed using MAFFT (v7.505) (Katoh et al. 2019) and manually optimized using AliView (v. 1.26) (Larsson 2014). Subsequently, the CIPRES Science Gateway (v. 3.3) (Miller et al. 2010) was used to perform the Maximum Likelihood (ML) analyses by using the IQ-TREE (v. 2.1.2) (Nguyen et al. 2015). ML search using IQ-Tree automatically selected the best substitution model and thereafter performed a thorough bootstrap with 1,000 replicates. The resulting trees were visualized and configured using iTOL (Letunic and Bork 2019). Bootstrap support values are placed at the top of the branches. Values less than 80% bootstrap support are not shown.

## DATA ORGANIZATION

We used the edibility information for each recorded species based on the Final Edibility Status (FES) proposed by Li et al. (2021) and detailed in Table 1. For each consulted reference, we recovered the documented data of the identification and consumption of each mushroom species recorded from Brazil to categorize the Record of Occurrence in Brazil (ROB) and the Documentation of Consumption in Brazil (DCB), according to the system proposed in Table 1. All data are compiled in the Supplementary Information 1.

**Table 1:** Categories used to classify the wild edible mushroom species occurring in Brazil

Category	Code	Description
Final edibility status (FES)	E1	Clear evidence that a species has been consumed without any adverse or harmful effects
	E2	Clear evidence that a species has been consumed after it has been cooked or prepared in such a way that it is safe and suitable for consumption. It also includes edible species that can cause allergic reactions or adverse responses when eaten with alcohol, for example
	E3	Evidence of safe consumption is uncertain or incomplete
	U	Unconfirmed edibility
	P	Causes adverse and harmful reaction when consumed
Record of occurrence in Brazil (ROB)	D	Occurrence confirmed based on molecular data (DNA sequence)
	T	Occurrence confirmed based on a nomenclatural type from Brazil
	M	Occurrence based on complete morphological description*
	S	Occurrence based on a short morphological description*
	L	Occurrence registered only in a list
Documentation of consumption in Brazil (DCB)	C	Clear record or documentation of consumption in Brazil
	R	Reports as edible in Brazil but with no clear documentation of consumption
	N	No documentation of consumption in Brazil

^*^To categorize the morphological descriptions presented in the references as M or S, it was considered the expertise of the taxonomists (authors of this work) that have worked on the data curation of each group of fungi

Based on the combination of FES, ROB, and DCB, we propose a final status to the Brazilian Edible Mushrooms (BEM) to categorize the occurrence and consumption of these mushroom species in Brazil, according to Table 2.

**Table 2:** Categories used to determine the final status of the Brazilian Edible Mushrooms (BEM)

Category	Code*	Description
BEM1	E1 + D + C; E1 + D + R; E1 + D + N; E1 + T + C; E1 + T + R; E1 + T + N	Edible species that clearly occurs and is consumed in Brazil or that represents a new food resource
BEM2	E2 + D + C; E2 + D + R; E2 + D + N; E2 + T + C; E2 + T + R; E2 + T + N	Edible species (after some previous preparing or cautions) that clearly occurs and is consumed in Brazil or that represents a new food resource
BEM3	E1 + M + C; E1 + S + C; E1 + L + C	Edible species consumed in Brazil but that requires further studies to confirm its identity and occurrence
BEM4	E2 + M + C; E2 + S + C; E2 + L + C	Edible species (after some previous preparing or cautions) consumed in Brazil but that requires further studies to confirm its identity and occurrence
BEM5	E1 + M + R; E1 + M + N; E1 + S + R; E1 + S + N; E1 + L + R; E1 + L + N	Edible species not clearly consumed in Brazil, and which requires further studies to confirm its identity and occurrence
BEM6	E2 + M + R; E2 + M + N; E2 + S + R; E2 + S + N; E2 + L + R; E2 + L + N	Edible species (after some previous preparing or cautions) not clearly consumed in Brazil, and which requires further studies to confirm its identity and occurrence
BEM7	E3 + D + R; E3 + D + N; E3 + T + R; E3 + T + N	Species that clearly occurs in Brazil but with unclear or missing evidence of safe consumption
BEM8	E3 + M + R; E3 + M + N; E3 + R + S; E3 + R + N; E3 + L + R; E3 + L + N	Species with unclear or missing evidence of safe consumption and that requires further studies to confirm its identity and occurrence
BEM9	U + D + R; U + D + N; U + T + R; U + T + N	Species that clearly occurs in Brazil but with unconfirmed edibility, including few poisonous records
BEM10	U + M + R; U + M + N; U + S + R; U + S + N; U + L + R; U + L + N	Species with unconfirmed edibility, including few poisonous records, and that requires further studies to confirm its identity and occurrence
P1	P + D + R; P + D + N; P + T + R; P + T + N	Poisonous species that clearly occurs in Brazil
P2	P + S + R; P + S + N; P + L + R; P + L + N	Poisonous species that requires further studies to confirm its identity and occurrence

^*^The meaning of the codes can be consulted in Table 1

## RESULTS

### Brazilian Edible Mushrooms

**Fig. 2:**
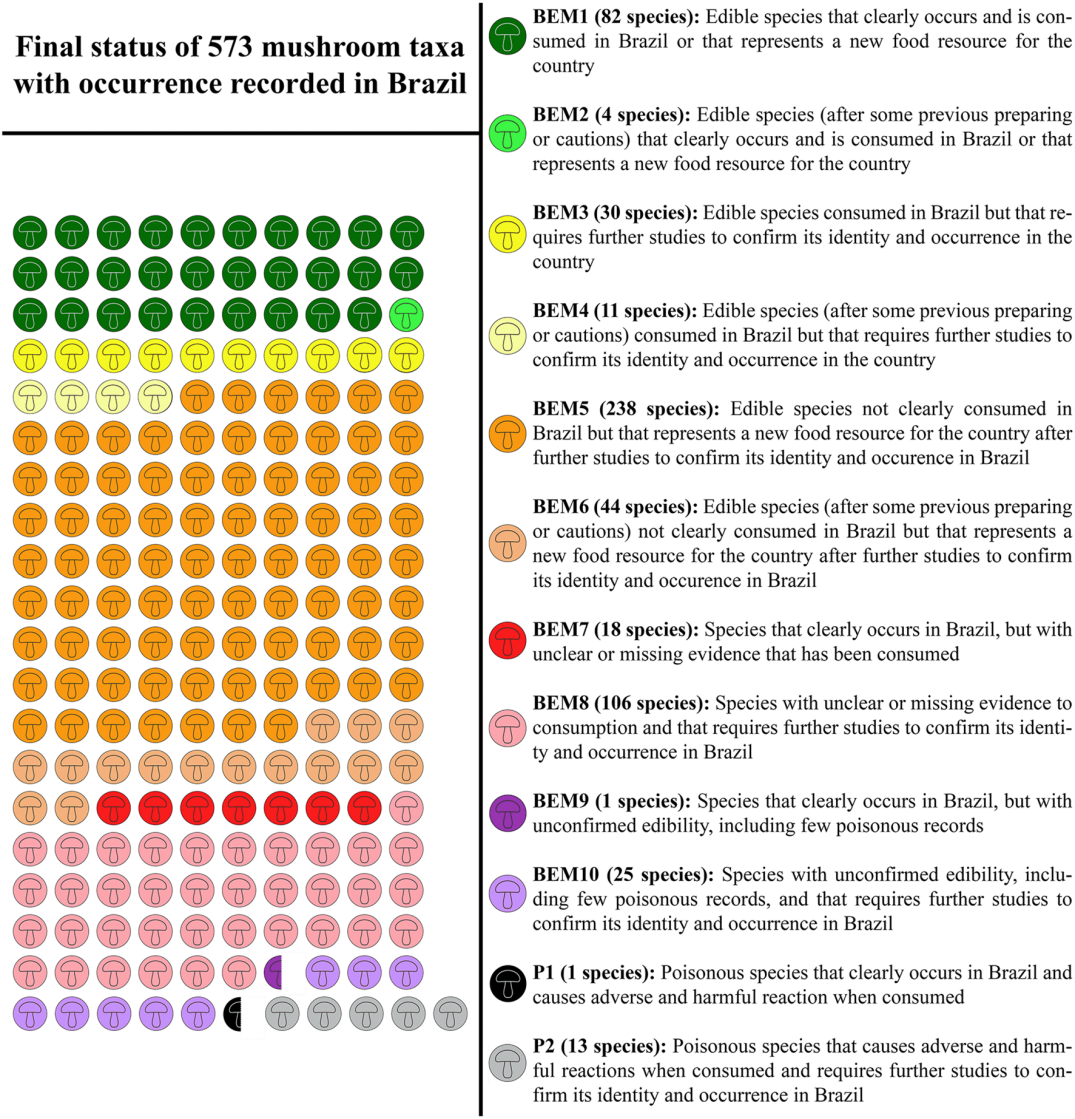
Final status of 573 macrofungi with occurrence recorded in Brazil

From the global list of 2786 macrofungal species (Li et al. 2021) plus the 13 species considered here as edible, we gathered records of the occurrence of 573 species in Brazil (Fig. 2) distributed in 10 edible categories (BEM1 to BEM10) plus two poisonous categories (P1 and P2). The complete dataset contains more than 3500 records of species occurrence from over 600 references (Supplementary Information 1).

For *Amanita dulciodora*, *Auricularia brasiliana*, *Auricularia tremellosa*, *Cookeina speciosa*, *Filoboletus gracilis*, *Gyroporus austrobrasiliensis*, *Lactarius hepaticus*, *Marasmiellus cubensis*, *Panus tephroleucus*, *Phlebopus beniensis*, *Pleurotus magnificus*, *Pseudohydnum viridimontanum*, and *Trechispora thelephora*, the FES was defined in this work (Supplementary Information 2). For five species, the FES was considered different from that proposed by Li et al. (2021): *Inonotus obliquus* was considered E1; *Lactarius taedae* and *Polyporus pes-simiae* were considered E3; *Stropharia coronilla* and *Chlorophyllum molybdites* were considered U (Supplementary Information 2).

There are records of 409 WEM in Brazil, of which 350 species can be consumed safely (BEM1, BEM3, BEM5), and 59 species that need some preparation to be safely consumed (BEM2, BEM4, BEM6) (Fig. 2). Among the 409 WEM recorded in the country, 86 species have a consistent record of occurrence in Brazil based on molecular data and/or Brazilian nomenclatural types (cf. Table 3, Figs. 4, 5, 6 and 7), being classified as BEM1 (82 species) and BEM2 (four species). Among the 86 species classified as BEM1 and BEM2, 52 are clearly consumed in Brazil, nine have uncertain or incomplete evidence of consumption in Brazil, and 25 are not consumed in the country and can be used as a new food resource (Table 3).

**Fig. 3:**
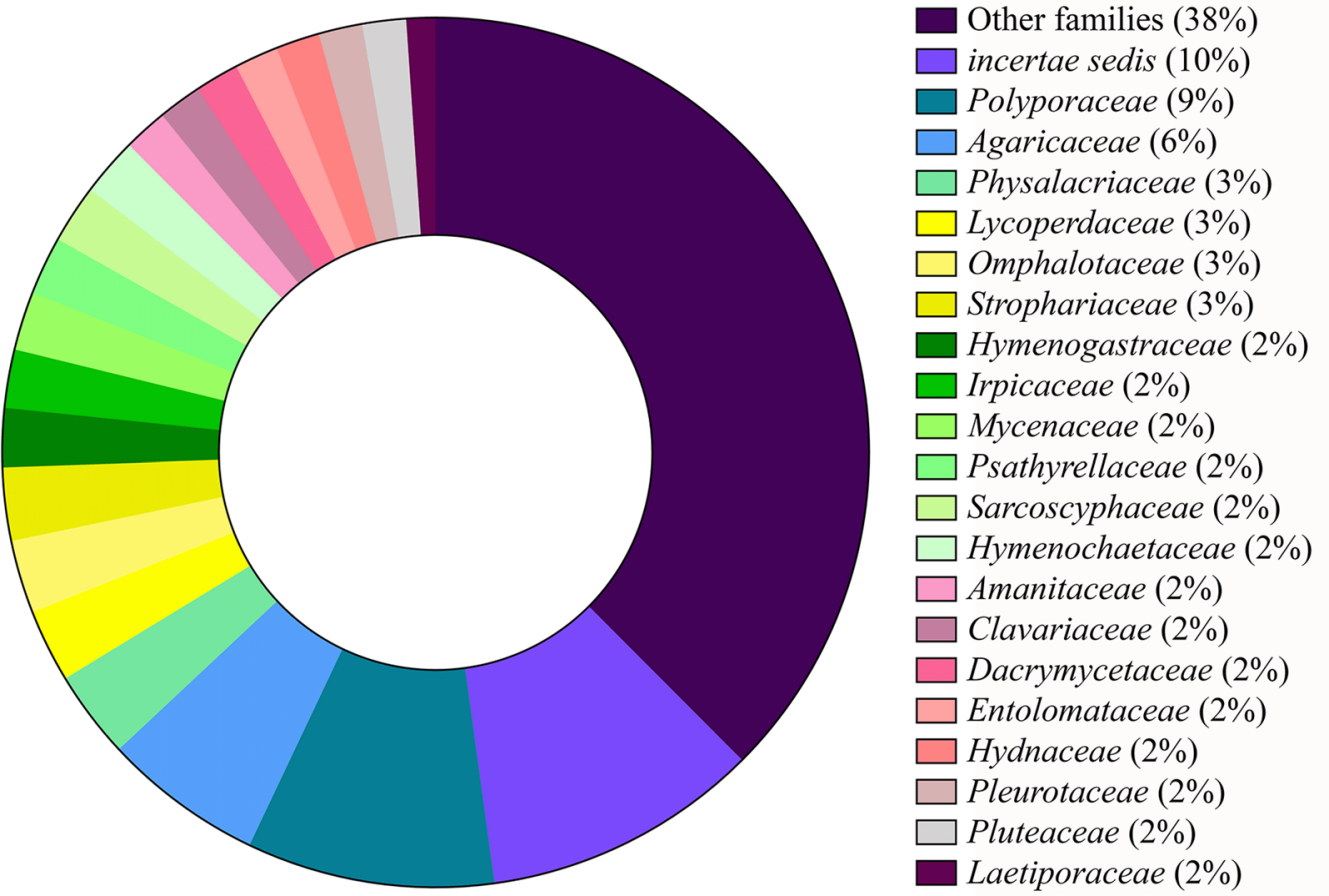
Relative proportion into families of the wild edible mushrooms with records to Brazil

**Table 3:**
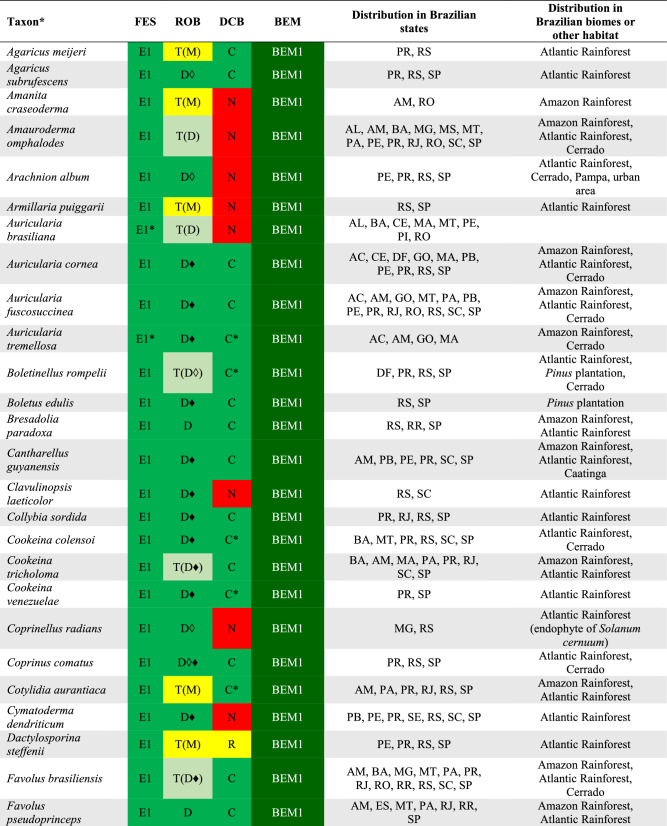

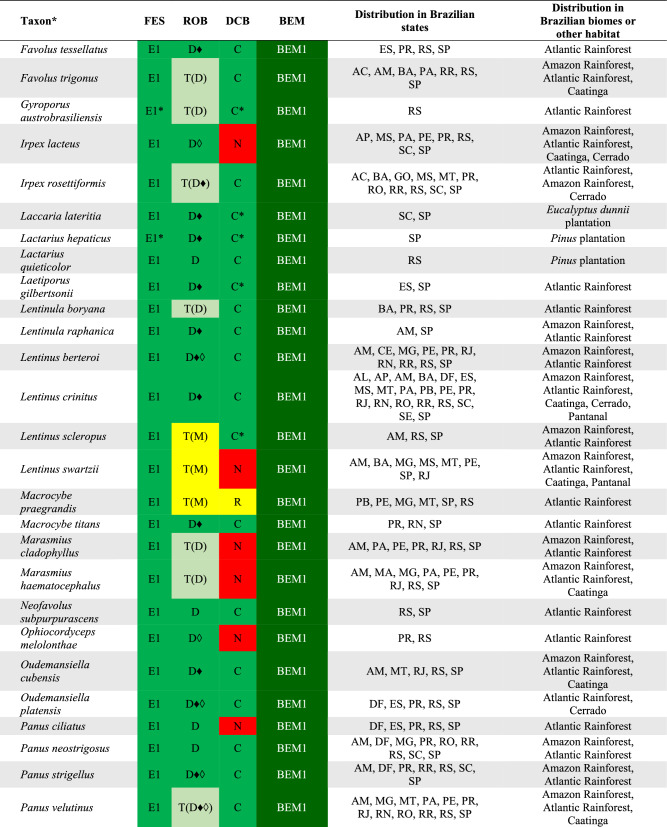

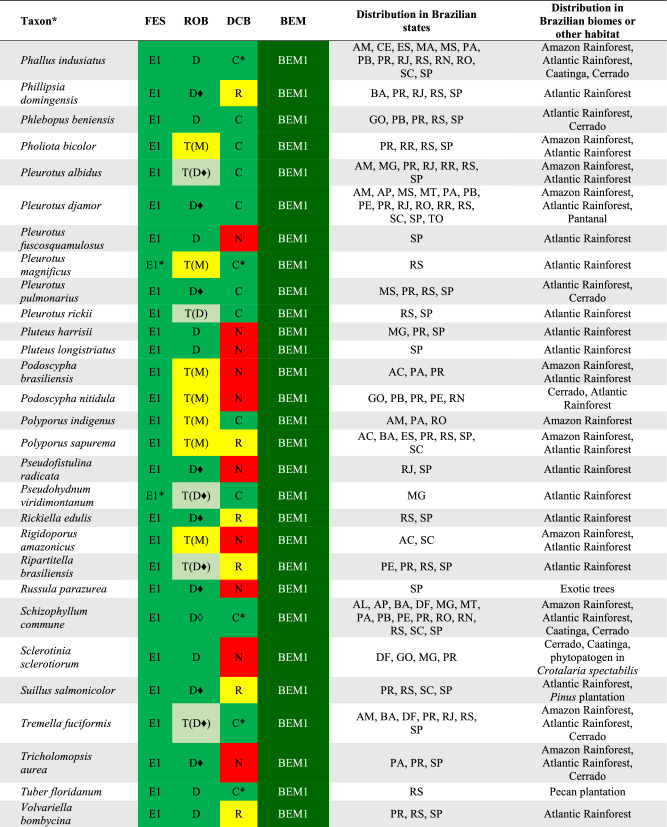

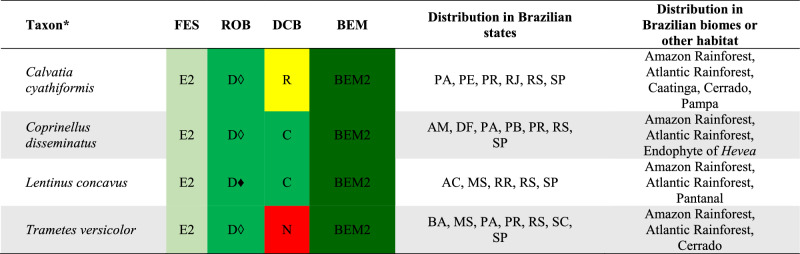
Species of wild edible mushrooms from Brazil classified into BEM1 and BEM2 categories

Taxon*: the complete citation for each species with their authorities can be consulted in the Supplementary Information 1. FES: Final Edibility Status, ROB: Record of occurrence in Brazil, DCB: Documentation of consumption in Brazil, BEM: Final status to the Brazilian edible mushrooms. The complete description of the FES, ROB, DCB, and BEM categories can be consulted in Tables 1 and Fig. 2. The complete name of Brazilian states and federative units can be consulted in Table 4. E*: edibility status defined in this work, C*: consumption in Brazil recorded in this work based on the authors’ experience, D♦: identity confirmed based on DNA sequence generated in this work, D◊: identity confirmed based on unpublished DNA sequence recovered from GenBank, T(D): occurrence confirmed based on nomenclatural type from Brazil and DNA sequence, T(M): occurrence confirmed based on nomenclatural type from Brazil and complete morphological description

**Fig. 4:**
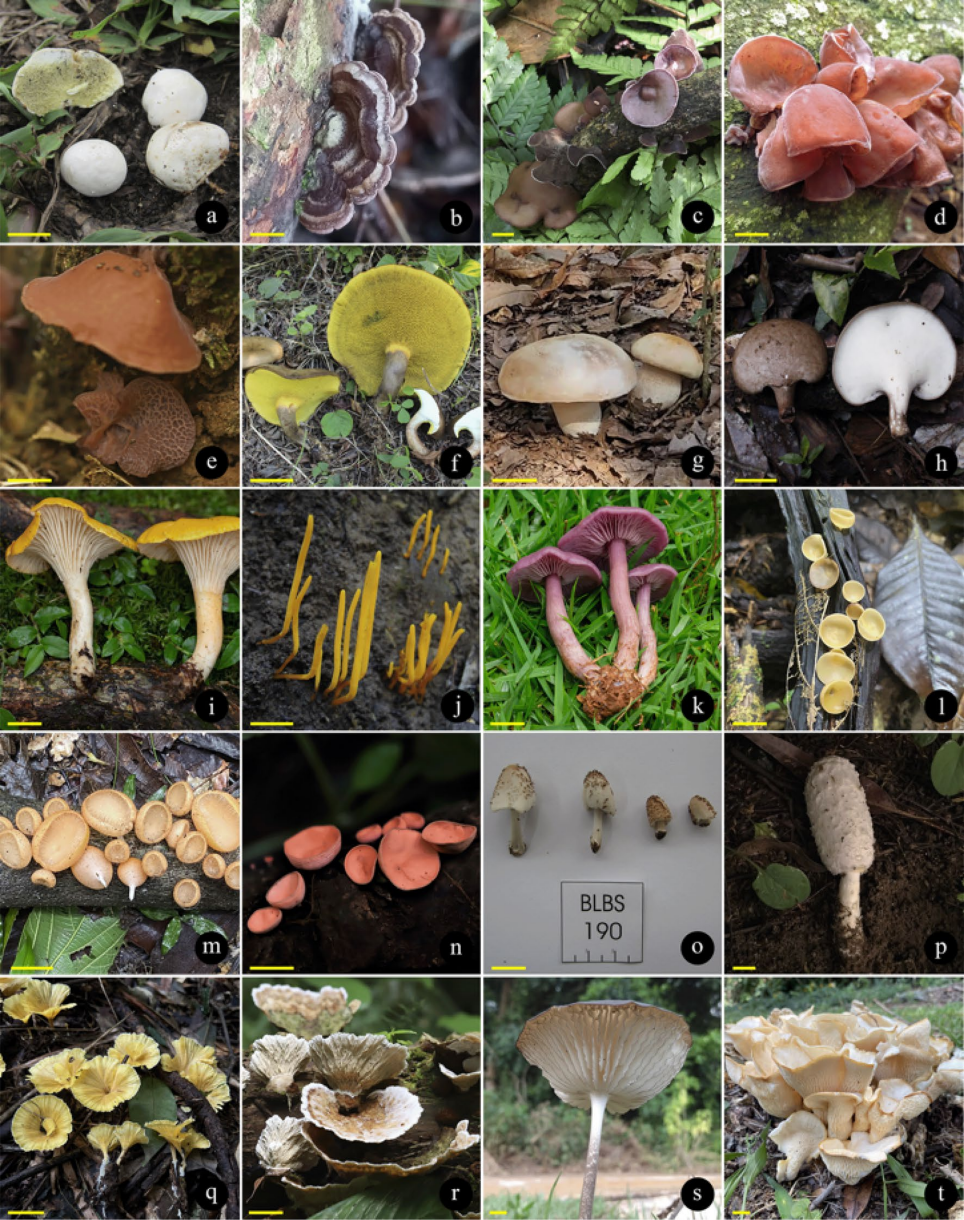
Wild edible mushrooms occuring in Brazil and classified as BEM1. **a** *Arachnion album*. **b** *Auricularia brasiliana*. **c** *Auricularia cornea*. **d** *Auricularia fuscosuccinea*. **e** *Auricularia tremellosa*. **f** *Boletinellus rompelii*. **g** *Boletus edulis*. **h** *Bresadolia paradoxa*. **i** *Cantharellus guyanensis*. **j** *Clavulinopsis laeticolor*. **k** *Collybia sordida*. **l** *Cookeina colensoi*. **m** *Cookeina tricholoma*. **n** *Cookeina venezuelae*. **o** *Coprinellus radians*. **p** *Coprinus comatus*. **q** *Cotylidia aurantiaca*. **r** *Cymatoderma dendriticum*. **s** *Dactylosporina steffenii*. **t** *Favolus brasiliensis*. Scale bars a–e, i–q, s,t = 1 cm, f–h, r = 3 cm. Photo courtesy of: (a,b,s) Larissa Trierveiler-Pereira; (c,d,l,p,q,r,t) Mariana Drewinski; (e,n) Marina Corrêa-Santos; (f) Altielys Magnago; (g) Sthefany Viana; (h) Amanda Micalloni; (i) Cristiano C. Nascimento; (j) Ariadne Furtado; (k) Denis Zabin; (m) Nelson Menolli Jr.; (o) Báraba L.B. Schünemann

**Fig. 5:**
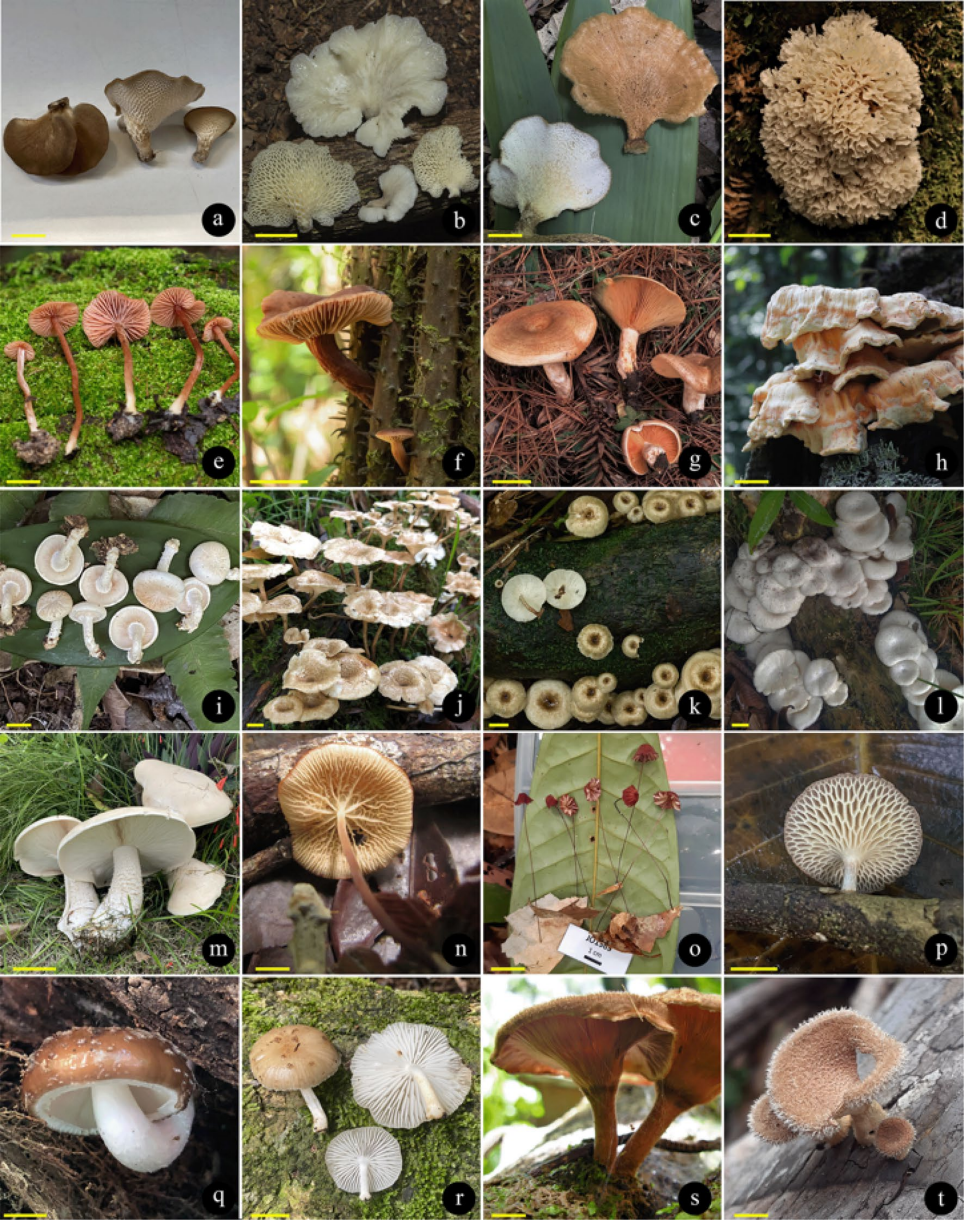
Wild edible mushrooms occuring in Brazil and classified as BEM1. **a** *Favolus pseudoprinceps*. **b** *Favolus tessellatus*. **c** *Favolus trigonus*. **d** *Irpex rosettiformis*. **e** *Laccaria lateritia*. **f** *Lactarius hepaticus*. **g** *Lactarius quieticolor*. **h** *Laetiporus gilbertsonii*. **i** *Lentinula raphanica*. **j** *Lentinus berteroi*. **k** *Lentinus crinitus*. **l** *Lentinus scleropus*. **m** *Macrocybe titans*. **n** *Marasmius cladophyllus*. **o** *Marasmius haematocephalus*. **p** *Neofavolus subpurpurascens*. **q** *Oudemansiella cubensis*. **r** *Oudemansiella platensis*. **s** *Panus ciliatus*. **t** *Panus neostrigosus*. Scale bars a–c, e, *i*–l, *n*–*t* = 1 cm, d, f–h, *m* = 3 cm. Photo courtesy of: (a,b,g,j,m,p,q,r) Mariana Drewinski; (c) Tamile Rodrigues (d,k,l) Marina Corrêa-Santos; (e) Denis Zabin; (f) Cristiano C. Nascimento; (h) Altielys Magnago; (i) Nelson Menolli Jr.; (n,o) Jadson Oliveira; (s) Fernanda Karstedt; (t) Ruby Vargas-Isla

**Fig. 6:**
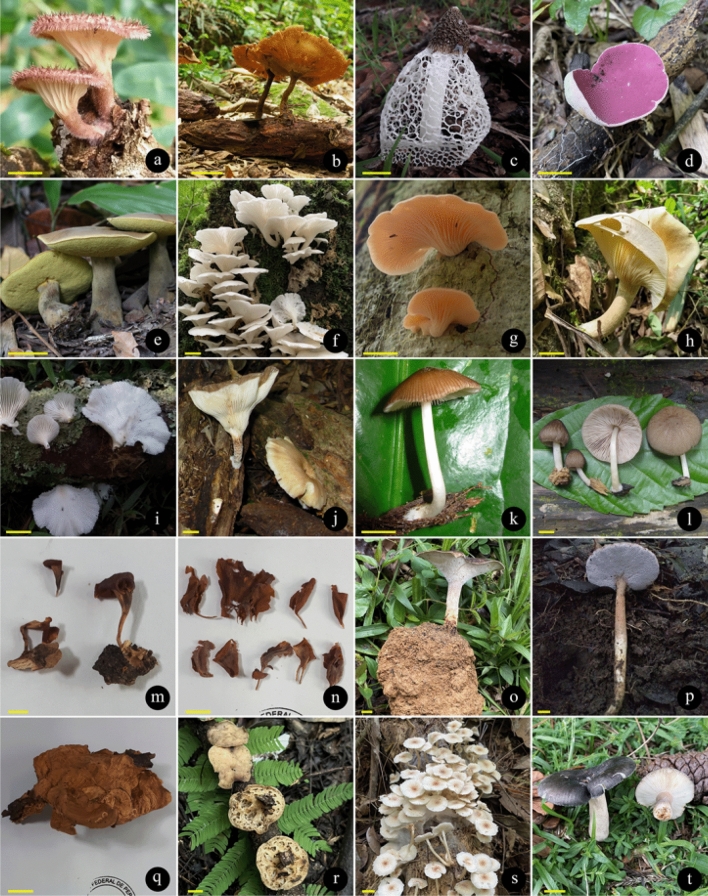
Wild edible mushrooms occuring in Brazil and classified as BEM1. **a** *Panus strigellus*** b** *Panus velutinus*. **c** *Phallus indusiatus*. **d** *Phillipsia domingensis*. **e** *Phlebopus beniensis*. **f** *Pleurotus albidus*. **g** *Pleurotus djamor*. **h** *Pleurotus magnificus*. **i** *Pleurotus pulmonarius*. **j** *Pleurotus rickii*. **k** *Pluteus harrisii*. **l** *Pluteus longistriatus*. **m** *Podoscypha brasiliensis*. **n** *Podoscypha nitidula*. **o** *Polyporus sapurema*. **p** *Pseudofistulina radicata*. **q** *Rigidoporus amazonicus*. **r** *Rickiella edulis*. **s** *Ripartitella brasiliensis*. **T** *Russula parazurea*. Scale bars a, d, f–t = 1 cm, b, c, e = 3 cm. Photo courtesy of: (a,d,h,i,p,r,s) Mariana Drewinski; (b,g) Denis Zabin; (c) Larissa Trierveiler-Pereira; (e) Maria Alice Neves; (f,o,t) Nelson Menolli Jr.; (j,k) Fernanda Karstedt; (l) Marina Capelari; (m,n,q) Tatiana Gibertoni

**Fig. 7:**
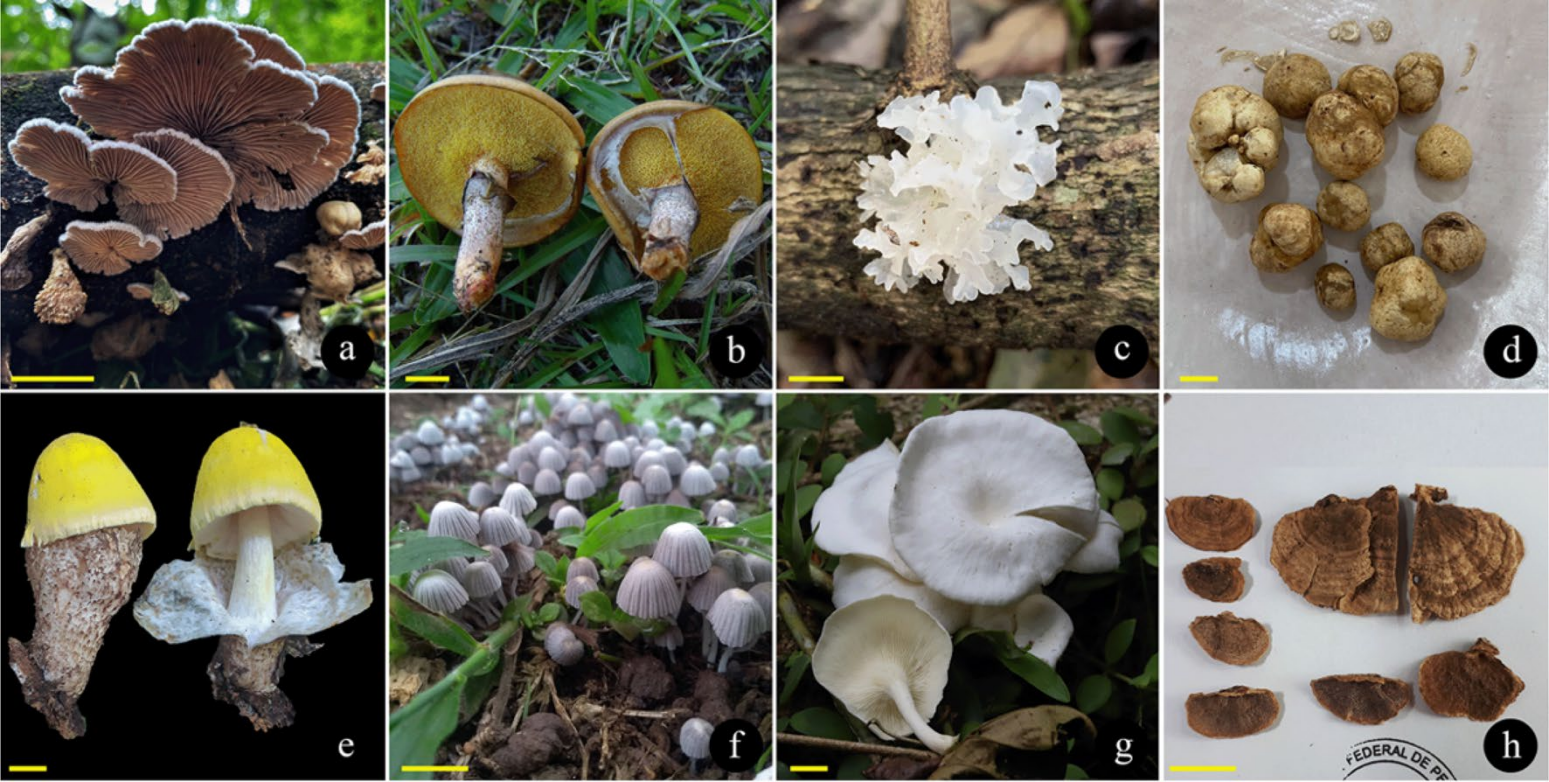
Wild edible mushrooms occuring in Brazil and classified as BEM1 (a–e) and BEM2 (f–h). **a** *Schizophyllum commune*. **b** *Suillus cothurnatus*** c** *Tremella fuciformis*. **d** *Tuber floridanum*. **e** *Volvariella bombycina*. **f** *Coprinellus disseminatus*. **g** *Lentinus concavus*. **h** *Trametes versicolor*. Scale bar = 1 cm. Photo courtesy of: (a) Nelson Menolli Jr.; (b) Altielys Magnago; (c,d,f) Mariana Drewinski; (e) Cristiano C. Nascimento; (g) Marina Corrêa-Santos; (h) Tatiana Gibertoni

A total of 41 WEM species were classified as BEM3 (30 species) and BEM4 (11 species), which represent edible species consumed in Brazil but further studies are need to confirm identity and occurrence of the respective mushrooms in Brazil. Most of the species were classified within BEM5 (238 species), which comprises edible species not clearly consumed in Brazil and that their occurrences were recorded based only on morphological characters. Other 150 species represent taxa with unclear or missing evidence for consumption (BEM7 and BEM8) or unconfirmed edibility status (BEM9 and BEM10), of which 19 (BEM7 + BEM9) clearly occur in Brazil and 131 (BEM8 + BEM10) require further studies to confirm their identity and occurrence in the country.

Finally, 14 species represent poisonous taxa, including one species that clearly occurs in Brazil (*Pseudomerulius curtisii*), and 13 species that require further studies to confirm their identity and occurrence in Brazil: *Bolbitius titubans*, *Clitocybe rivulosa*, *Conocybe apala*, *Conocybe tenera*, *Deconica merdaria*, *Hebeloma sacchariolens*, *Lepiota cristata*, *Leucoagaricus badhamii*, *Leucocoprinus birnbaumii*, *Lysurus arachnoideus*, *Mutinus caninus*, *Psathyrella corrugis*, and *Tapinella panuoides*.

The 409 WEM species belong to 184 genera in 76 families (classification based on He et al. 2019 for *Basidiomycota* and Wijayawardene et al. 2018 for *Ascomycota*), and most of the species belongs to the phylum *Basidiomycota* (389 species = 95,11%). From these 409 species, the families with the highest number of genera (Fig. 3) are *Polyporaceae* (17 genera and 44 species), *Agaricaceae* (11 genera and 41 species), *Physalacriaceae* (six genera and nine species), *Lycoperdaceae* (five genera and 14 species), *Omphalotaceae* (five genera and 16 species), and *Strophariaceae* (five genera and 12 species). *Agaricus* was the genus with the highest number of recorded edible species (21 species) followed by *Pleurotus* (14 species), *Lentinus* (13 species), *Laccaria* (10 species), *Auricularia* (nine species), and *Macrolepiota* (eight species). Considering only the 86 species classified as BEM1 and BEM2, the genera with the highest number of species that clearly occur in Brazil are: *Pleurotus* (six species), *Lentinus* (five species), *Favolus* (four species), *Auricularia* (four species), *Panus* (four species), and *Cookeina* (three species).

The Brazilian states (Table 4) with the highest number of recorded WEM species are Rio Grande do Sul (260 species), São Paulo (200 species), and Paraná (167 species). The states with the lowest number of WEM species recorded are Sergipe (seven species), Piauí (five species), and Tocantins (one species). Considering only the 86 species classified as BEM1 and BEM2, the Brazilian states with the highest number of species clearly occuring there are: São Paulo (69 species), Rio Grande do Sul (60 species), and Paraná (53 species). The species with the highest number of records were *Pycnoporus sanguineus* (41 records in 16 states), *Lentinus tricholoma* (38 records in 17 states), and *Lentinus crinitus* (44 records in 20 states).

**Table 4:** Distribution of the 409 species of wild edible mushrooms recorded from Brazil

Brazilian states	BEM1	BEM2	BEM3	BEM4	BEM5	BEM6	Total
AC (Acre)	8	1	3	–	2	–	14
AL (Alagoas)	5	–	4	–	4	–	13
AM (Amazonas)	26	1	13	–	23	1	64
AP (Amapá)	4	–	2	–	8	–	14
BA (Bahia)	14	1	6	–	11	1	33
CE (Ceará)	4	–	1	1	5	1	12
DF (Distrito Federal)	10	1	4	–	4	–	19
ES (Espírito Santo)	8	–	1	–	4	1	14
GO (Goiás)	7	–	1	–	5	–	13
MA (Maranhão)	6	–	2	–	5	–	13
MG (Minas Gerais)	14	–	2	–	9	–	25
MS (Mato Grosso do Sul)	8	2	1	–	11	3	25
MT (Mato Grosso)	14	–	3	–	14	–	31
PA (Pará)	18	3	9	–	19	–	49
PB (Paraíba)	11	1	5	–	14	1	32
PE (Pernambuco)	20	1	8	1	28	5	63
PI (Piauí)	1	–	–	1	2	1	5
PR (Paraná)	50	3	12	7	84	11	167
RJ (Rio de Janeiro)	20	1	5	–	19	2	47
RN (Rio Grande do Norte)	7	–	3	–	7	2	19
RO (Rondônia)	13	–	9	1	14	–	37
RR (Roraima)	13	1	5	1	4	–	24
RS (Rio Grande do Sul)	56	4	18	11	137	34	260
SC (Santa Catarina)	20	1	8	3	32	5	69
SE (Sergipe)	2	–	2	–	3	–	7
SP (São Paulo)	65	4	16	8	88	19	200
TO (Tocantins)	1	–	–	–	–	–	1

The description of the codes for the Brazilian Edible Mushroom (BEM) status can be consulted in Table 2 and Fig. 2

**Fig. 8:**
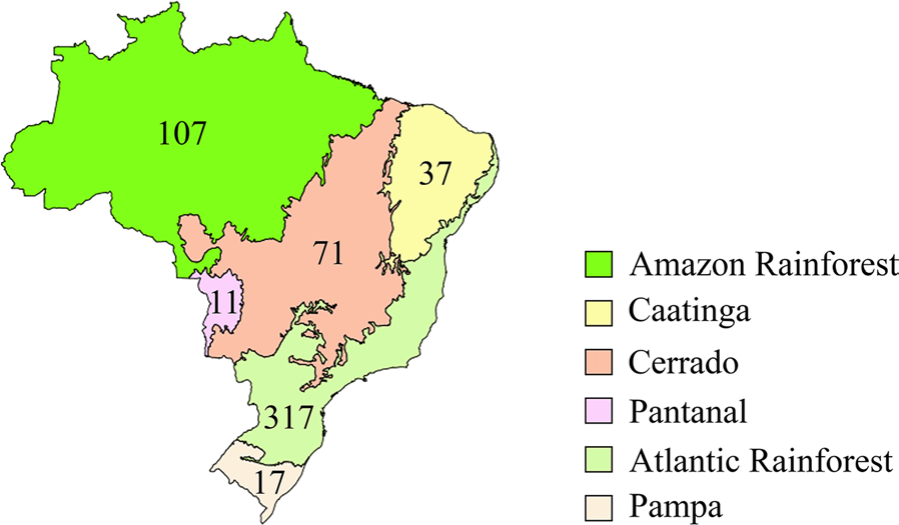
Distribution of the 409 wild edible mushroom species in the Brazilian biomes

Regarding the distribution of species in Brazilian biomes (Fig. 8), most of the 409 WEM species are recorded for the Atlantic Rainforest (317 species) and the Amazon Rainforest (107 species), with 34 species recorded for both biomes. For the Cerrado biome, 71 WEM species were recorded, of which 17 are also recorded for the Atlantic Rainforest and 18 are also found in the Atlantic and Amazon Rainforests. For the Caatinga biome, 37 WEM were recorded, of which 11 species were also recorded for the Atlantic and Amazon Rainforests, and 12 species that also occur in Cerrado and the Atlantic and Amazon Rainforest biomes. The Pantanal and Pampa biomes registered the lowest number of edible species, 11 and 17, respectively. Other 32 species were recorded in exotic forests (mainly *Pinus* spp. and *Eucalyptus* spp. plantations). These species form ectomycorrhizal associations and belong to the genera *Amanita*, *Boletus*, *Chalciporus*, *Clavulina*, *Laccaria*, *Lactarius*, *Pisolithus*, *Ramaria*, *Rhizopogon*, *Russula*, *Suillus*, and *Tuber* (Rinaldi et al. 2008) with non-native species and were introduced with the symbiotic trees. Considering only the 86 species classified as BEM1 and BEM2, 75 species were recorded for the Atlantic Rainforest and 40 species for the Amazon Rainforest.

A total of 32 WEM species have a nomenclatural type associated with specimens collected in Brazil and were classified as BEM1 (Table 3). Other ten species with Brazilian holotypes were classified as BEM7 due to their FES as E3 (Li et al. 2021). Among the species with a Brazilian holotype and classified as BEM1, *Amauroderma omphalodes*, despite not knowing its consumption in Brazil, was one of the most widely distributed taxa in the country, being reported in 22 references for 13 states; followed by *Panus velutinus* that was reported in 32 references for 12 states; *Favolus brasiliensis*, reported in 24 references for 12 states; and *Irpex rosettiformis*, reported in 36 references for 11 states. Data on the distribution of species in Brazilian states and biomes (Supplementary Information 1) were based only on information that was explicitly indicated in the literature consulted or from newly samples from known biomes, and thus the interpretation of this distribution must be done with caution because not all consulted references specified the Brazilian state and biome where the specimens were collected.

## MOLECULAR STUDIES

We generated 143 new sequences (136 ITS and seven LSU) representing 40 species within 29 genera (information on the GenBank accession numbers and phylogenetic trees are available in the Supplementary Information 3). For 18 species we provide the first ITS sequences from specimens collected in Brazil: *Boletus edulis*, *Clavulinopsis laeticolor*, *Collybia sordida*, *Cookeina colensoi*, *Cookeina tricholoma*, *Cookeina venezuelae*, *Cymatoderma dendriticum*, *Laccaria lateritia*, *Lactarius hepaticus*, *Lentinus concavus*, *Macrocybe titans*, *Oudemansiella cubensis*, *Pseudofistulina radicata*, *Rickiella edulis*, *Ripartitella brasiliensis*, *Russula parazurea*, *Tremella fuciformis*, and *Tricholomopsis aurea*.

Based on newly generated sequences, we report for the first time the occurrence of *Lactarius hepaticus* and *Russula parazurea* in Brazil. Additionally, 15 WEM species are new records for the following Brazilian states: Maranhão (*Auricularia tremellosa*), Tocantins (*Pleurotus djamor*), Mato Grosso do Sul (*P. djamor*), Espírito Santo (*Laetiporus gilbertsonii* and *Oudemansiella platensis*), Rio de Janeiro (*Collybia sordida*, *Phillipsia dominguensis*, and *Tremella fuciformis*), Rio Grande do Norte (*Macrocybe titans*), São Paulo (*Boletus edulis*, *Cantharellus guyanensis*, *Cookeina venezuelae*, *Cookeina tricholoma*, *Lactarius hepaticus*, *Lentinus concavus*, *Oudemansiella platensis*, and *Russula parazurea*), and Paraná (*O. platensis*).

We recovered 313 sequences previously available in Genbank that are related to 76 names of species collected in Brazil. The ML analyses confirmed the specific identity of 287 sequences representing 63 species (Supplementary Information 3), but 26 sequences of 13 species are unconfirmed (Table 5) as discussed in Supplementary Information 4.

**Table 5:** Unconfirmed sequences available in Genbank of supposed wild edible mushrooms collected in Brazil

Taxa	GenBank access	References	Identity
*Bjerkandera adusta*	KJ832002	Martin et al. (2015)	Misidentified
*Bolbitius demangei*	KX246930	Melo et al. (2016)	Unconfirmed
*Coriolopsis rigida*	KR812261	Reis et al. (2015)	Misidentified
	MN991225	Unpublished	Misidentified
*Daldinia concentrica*	JX944137	Sia et al. (2013)	Misidentified
*Laccaria lateritia*	KY081710, KY081711	Sulzbacher et al. (2018)	Misidentified
*Mycena chlorophos*	KJ831841	Martin et al. (2015)	Misidentified
*Ophiocordyceps sobolifera*	AY754003, AY746002, AY745997	Rubini et al. (2005)	Misidentified
*Oudemansiella canarii*	HQ534101, HQ377277	Vieira et al. (2012)	Misidentified
	AY216474	Unpublished	Misidentified
	KJ620018	Unpublished	Misidentified
*Panus similis*	MT669126, MT669127, MT669128	Unpublished	Misidentified
*Panus tephroleucus*	MN602052	Unpublished	Unconfirmed
*Phanerochaete sordida*	HQ377285, HM997134	Vieira et al. (2012)	Misidentified
	KR812274	Reis et al. (2015)	Misidentified
	JX944113	Sia et al. (2013)	Misidentified
*Rigidoporus lineatus*	KP859302	Unpublished	Misidentified
*Rigidoporus microporus*	KP859298, KP859300	Unpublished	Misidentified

## DISCUSSION

The number of 409 species recovered as the WEM occurring in Brazil must be interpreted with caution because it includes taxa that need further taxonomic investigation, although this number is a starting point for future studies on WEM from Brazil. The implementation of sequencing procedures has revealed misidentifications even with species of high economic and cultural importance around the world. Wu et al. (2014) performed morphological and phylogenetic analyses and concluded that the most important cultivated species of *Auricularia* in China, viz. *Auricularia heimuer*, has been misidentified for years as *Auricularia auricula-judae*, a species originally described from and probably restricted to Europe (Wu et al. 2021). A similar example also is found in Brazil, where Silva-Filho et al. (2020), based on molecular and morphological identification approaches, recently confirmed that some specimens commercialized in Brazil as *Lactarius deliciosus* actually represent *Lactarius quieticolor*.

In addition to assisting in the delimitation and certification of species identity, DNA-barcoding also can be a powerful tool for a reliable identification and quality control of food products (Ángeles-Argáiz and Garibay-Orijel 2020). Dentinger and Suz (2014) used DNA-sequencing to analyze a commercial packet of dried porcini and found three undescribed species of mushrooms inside it. In the same way, Cutler II et al. (2021) also used molecular analysis to study 16 food products labeled as containing wild mushrooms and verified that only five products contained the species described on the label and, more alarmingly, that some products contained species of dubious edibility or potentially toxic (Cutler II et al. 2021). Misidentification and potentially intentional mislabeling in other food products, including endangered species, such as shark meat (Almerón-Souza et al. 2018), seafood (Minoudi et al. 2020; Giusti et al. 2023), and other fish meat (Liu et al. 2022) have also been found with the aid of molecular techniques.

Considering the importance of molecular and distribution studies for an accurate identification of species, 86 WEM species were considered to have a robust occurrence record in Brazil. These species (within BEM1 and BEM2 categories) are those that have DNA sequences available from Brazilian specimens or those that were originally described from Brazil. Other 323 species need taxonomic studies to confirm their identity and occurrence in the country (species categorized in BEM3–BEM6) because many have been mentioned only in lists (69 species) or based on short and incomplete morphological descriptions (108 species). A total of 41 species (BEM3 + BEM4) needs to be studied more urgently because they are species consumed by part of the Brazilian population. The species categorized in BEM5 and BEM6 add up to 282 taxa that are not consumed in Brazil but can represent new food resources for the country after their identity and occurrence in Brazil are confirmed. Among the 323 species that require additional studies on their identification, we highlight some taxa that most likely do not occur in the country or that involve taxonomic issues to be better investigated.

*Auricularia delicata* is a species commonly reported to Brazil for more than 120 years (Hennings 1900; Teixeira 1945; Batista et al. 1966; Fidalgo 1968; Lowy 1971; Capelari and Maziero 1988; Goés-Neto 1996; Drechesler-Santos et al. 2008; Alvarenga and Xavier-Santos 2015; Santos 2017; Couceiro et al. 2019; Cavalcante et al. 2021; Nascimento et al. 2021) but that represents a species complex, with probably a different taxon restricted to the country. Wu et al. (2021) accepted *A. tremellosa* as an independent species within the *A. delicata* complex based on morphological and phylogenetic analyses. They studied six Brazilian specimens, and the characters studied fit in *A. tremellosa*, a species originally described from Mexico. *Auricularia delicata* was originally described from Western Africa (Fries 1830) and may have a more restricted distribution (Wu et al. 2021). Regarding the geographical distribution of *Auricularia* species, Wu et al. (2021) concluded that most species are restricted to a unique continent, whereas few species are widely distributed, e.g., *Auricularia cornea*.

The genus *Agaricus*, although containing the largest number of WEM species recorded here (21 species), still needs to be better investigated in the country since only two species, *Agaricus meijeri* and *Agaricus subrufescens*, were categorized in BEM1. The identification of *Agaricus* species can be challenging since the species have a limited number of morphological characteristics that can change due to environmental factors and intraspecific variability (Zhao et al. 2011). Phylogenetic studies have shown that tropical and non-tropical species of *Agaricus* are generally grouped in distinct clades, and new tropical species have been identified and described (Zhao et al. 2011, 2016; Chen et al. 2017; Ortiz-Santana et al. 2021; Medel-Ortiz et al. 2022).

*Favolus tenuiculus* is another example of species that probably does not occur in Brazil but remains under many records in the country for more than 80 years (Torrend 1938; Singer 1961; Bononi et al. 1981; Rajchenberg and Meijer 1990; Loguercio-Leite 1990, 1992; Loguercio-Leite and Wright 1991; Bononi 1992; Gugliotta and Capelari 1995; Gerber 1996; Góes-Neto 1999; Gonçalves and Loguercio-Leite 2001; Groposo and Loguercio-Leite 2002, 2005; Gibertoni and Cavalcanti 2003; Góes-Neto et al. 2003; Gibertoni et al. 2004, 2007; Meijer 2006, 2008; Silveira 2006; Louza and Gugliotta 2007; Abrahão et al. 2012; Neves et al. 2013; Santos 2017; Timm 2018; Couceiro et al. 2019). Although *Favolus brasiliensis* has been treated as synonymous of *Favolus tenuiculus* (= *Polyporus tenuiculus*), the latter is considered a dubious name (Palacio et al. 2021) originally described from Nigerian material (Palisot-Beauvois 1804) and most likely is not the correct name to be applied for Brazil's specimens. *Favolus brasiliensis* is the type species of the genus *Favolus* and was described from Brazil (Fries 1828). Based on molecular investigations, Palacio et al. (2021) and Zabin et al. (2024) studied *Favolus* from the Neotropics and concluded that *F. brasiliensis* is an appropriate name for Neotropical samples better than *F. tenuiculus*. Despite this information, as not all specimens recorded in the consulted bibliographies as *F. tenuiculus* were studied and re-identified, the record of *F. tenuiculus* remains on the list as BEM3, requiring further taxonomic investigations.

Some worldwide cultivated species have been reported from Brazil and here classified as BEM3, such as *Lentinula edodes* (Timm 2018, 2021) and *Pleurotus ostreatus* (Rick 1938, 1961; Singer 1953; Batista and Bezerra 1960; Pereira 1988; Meijer 2001, 2006, 2008; Lyra et al. 2009; Couceiro et al. 2019; Putzke and Putzke 2019; Cavalcante et al. 2021). However, recent molecular studies with samples from Brazil and/or other countries from the Neotropics showed that these species most likely do not occur in Brazil (Menolli et al. 2014a, 2022).

These are just a few examples of the importance of accurate taxonomic studies with WEM species. In addition to systematic investigations, it is also important to carry out ethnomycological studies in Brazil to better investigate the relationships of people with fungi and the possible occurrence of currently unknown edible species or to confirm the edibility of already known species. Studies from the last two decades, mostly based on morphological and molecular data, have described new species of fungi from Brazil in genera that are known to contain edible species, such as *Agaricus* (Drewinski et al. 2017), *Amanita* (Nascimento et al. 2018), *Armillaria* (Lima et al. 2008), *Auricularia* (Wu et al. 2021), *Cantharellus* (Wartchow et al. 2012), *Favolaschia* (Capelari et al. 2013), *Favolus* (Palacio et al. 2021), *Gymnopus* (Coimbra et al. 2015), *Lactarius* (Silva-Filho et al. 2018), *Macrolepiota* (Perez et al. 2018; Freitas and Menolli 2019; Souza et al. 2022), *Marasmius* (Oliveira et al. 2014, 2020a, 2020b, 2022, 2024a, b), *Panus* (Sousa-Guimarães et al. 2024), *Pluteus* (Menolli et al. 2014b, 2015), *Pseudohydnum* (Coelho-Nascimento et al. 2024), *Tuber* (Grupe II et al. 2018), and *Volvariella* (Menolli and Capelari 2008).

Despite the benefits of eating wild mushrooms, there is also a concern related to toxic species. In Brazil, few cases of poisoning by wild mushrooms have been reported in the scientific literature (Meijer 2001; Meijer et al. 2007), although some other cases have been spread in popular media. Meijer et al. (2007) described in detail the poisoning of four people of the same family by consumption of *Chlorophyllum molybdites* from the state of Paraná, Southern Brazil. *Chlorophyllum molybdites* is similar to edible species of the genus *Macrolepiota* and can be easily confused by untrained people. An accurate identification and use of the correct scientific name are the most useful way to search if a species is edible, medicinal, or poisonous (Boa 2004). The 14 species classified here as poisonous represent only the taxa that were listed by Li et al. (2021) but not all toxic species that may occur in Brazil. Therefore, the consumption of wild mushrooms must be done responsibly, mainly when it comes from genera with both edible and poisonous species, such as *Agaricus* and *Amanita*. The edible *Amanita craseoderma* and the lethal *Amanita phalloides*, both occurring in Brazil (Bas 1978; Scheibler 2019), are good examples of this matter.

Although there is an incredible biodiversity of WEM, just five genera accounting for 85% of the world’s mushroom supply (Royse et al. 2017): *Lentinula* (22%), *Pleurotus* (19%), *Auricularia* (18%), *Agaricus* (15%), and *Flammulina* (11%). While tropical regions have the potential to be a valuable source of cultivable species of mushrooms (Thawthong et al. 2014), most of the strains commonly used for commercial purposes come from species that occur in temperate climate areas, but the techniques that are used to cultivate one species may be applied for cultivating another one, adapting the substrate or altering the growing environment (Stamets 2000). People around the world enjoy eating mushrooms and there is a huge potential for introducing new domesticated tropical mushrooms to the regional and global market (Thawthong et al. 2014). There are a lot of species with potential for cultivation that could contribute to food self-sufficiency, creation of local jobs, and poverty mitigation, improving the food security and food sovereignty scenario (Pérez-Moreno et al. 2021b).

Mushroom cultivation is an expanding activity in Brazil (Gomes et al. 2016) but it is still restricted to commercial strains of species from temperate climate areas, being the Brazilian production dominated by *Pleurotus ostreatus*, *Lentinula edodes*, and *Agaricus bisporus* (Gomes et al. 2016; Ishikawa et al. 2017). Previous papers focused on the cultivation of WEM from Brazil dates back to the last two decades, with species of the genera *Oudemansiella* (Ruegger et al. 2001) and *Macrolepiota* (Maki and Paccola-Meirelles 2002). Lately, Drewinski et al. (2024) published a study on the cultivation of a wild strain of *Auricularia cornea* from Brazil.

The recent record (Grupe II et al. 2018) of a “true truffle” of the genus *Tuber* in pecan (*Carya illinoinensis*) plantations in Brazil has intensified the study of truffle cultivation in the country (Sulzbacher et al. 2019; Freiberg et al. 2021). Allied to the growing attention in commercial mushrooms is the interest in WEM for both production and consumption. Some mycological tourism initiatives focusing on WEM have already started in the North, Northeast, Southeast, and South regions of Brazil, and responsible information about edible mushroom species on social media has shown to be very important to increase knowledge in countries with no tradition of foraging wild mushrooms such as Brazil. The increasing interest in foraging and the commercial importance of WEM emphasize the need for reliable information about species to avoid misidentification and poisoning (Li et al. 2021).

Fungi represent one of the greatest untapped resources of nutritious food in the world (Wani et al. 2010). Boa (2004) summarizes the importance of WEM in three main reasons: i) as a source of food and health benefits; ii) as a source of income, especially for rural people; and iii) to maintain the health of the forests, as fungi constitute fundamental components of the ecosystems. Fungi are not immune to the threats that put animal and plant species at risk (Mueller et al. 2014). Efforts have been made to evaluate fungal species into the red-listing system of the International Union for Conservation of Nature (IUCN) and to emphasize the importance of the conservation of fungi (Mueller et al. 2014, 2022).

Currently, there are 597 species of fungi published in the Red List, with 133 of them being used for human food (Mueller et al. 2022). Considering the 409 WEM recorded to Brazil, only 22 species have been already evaluated on the Global Fungal Red List Initiative (Mueller et al. 2022): *Agaricus arvensis*, *Agaricus campestris*, *Agaricus sylvaticus*, *Boletus edulis*, *Calocybe gambosa*, *Cantharellus cinnabarinus*, *Cantharellus guyanensis*, *Coprinus comatus*, *Lycoperdon perlatum*, *Suillus granulatus*, and *Suillus luteus* were assessed as Least Concern; *Pleurotus rickii*, and *Polyporus sapurema* were classified as Near Threatened; *Clavaria zollingeri* was categorized as Vulnerable; and *Rickiella edulis* as Endangered. Focusing on access the global conservation status of the species listed in this work, the BEM Conservation Initiative (https://redlist.info/iucn/species_list/event/26) was recently created, and as part of our first results seven species have already been assessed: *Favolus tesselatus*, *Lentinula raphanica*, and *Lentinus scleropus* as Least Concern; *Lentinus concavus*, and *Pleurotus albidus* as Near Threatened, *Bresadolia paradoxa* as Vulnerable, and *Pleurotus magnificus* as Endangered.

The 2030 Agenda for Sustainable Development is a plan of action for people, planet, and prosperity adopted by United Nation Members in 2015, including Brazil. The 17 Sustainable Development Goals summarize the areas of critical importance for humanity and planet (United Nation 2015). Pérez-Moreno et al. (2021a) linked edible mycorrhizal fungi strategies to achieve 11 of the 17 goals. According to them, edible mushrooms may promote forest sustainability, biodiversity conservation, food supply, nutrition and health, biocultural conservation, women empowerment, and economic development (Pérez-Moreno et al. 2021a). Thus, it is extremely relevant to develop strategies to preserve WEM genetic resources for food security (Pérez-Moreno et al. 2021b). Edible mushrooms are an important non-wood forest product and the knowledge about them can add value to the forests, increasing the incentive to protect natural areas.

## CONCLUSION

We summarized the information about the records of 409 wild edible mushrooms in Brazil, of which 350 species can be consumed safely and 59 species that need some preparation to be safely consumed. Consistent occurrence records were found for 86 species, reinforcing the need for further studies to confirm the specific identity of at least other 323 edible mushrooms reported to Brazil. A total of 41 species needs some urgency in these studies because they represent species consumed by part of the Brazilian population, whereas the other 282 taxa are not consumed in Brazil but can represent new food resources for the country after further studies to confirm their identities. The edible mushrooms may be used not just as an excellent nutritional and functional food but also in industrial applications and in research and development of drugs. Wild edible mushrooms are a valuable natural resource still underutilized and represent a non-timber forest product with important ecological, socio-cultural, medicinal, and economic relevance.

## Supplementary Information

Additional file 1

Additional file 2

Additional file 3

Additional file 4

## Data Availability

The sequencing data is available from a public database and the information is provided in the Supplementary Information 1. Other data will be made available on request.

## Abbreviations

BEM: Final status to the Brazilian Edible Mushrooms
BLAST: Basic Local Alignment Search Tool
CTAB: Cetyltrimethylammonium bromide
DCB: Documentation of Consumption in Brazil
DNA: Deoxyribonucleic Acid
ITS: Internal Transcribed Spacer
LSU: Large Subunit Ribosomal Ribonucleic Acid
ML: Maximum Likelihood
PCR: Polymerase Chain Reaction
PDA: Potato Dextrose Agar
ROB: Record of Occurrence in Brazil
WEM: Wild Edible Mushrooms
FES: Final Edibility Status

## Footnotes

1 Although macrofungi of different forms have distinctive popular names (mushrooms, brackets, puffballs, truffles, false-truffles, cup fungi etc.), we will refer to wild edible macrofungi as wild edible mushrooms (WEM) throughout the text.

## Declarations

### Adherence to national and international regulations

This study is according to the Brazilian legislation on access to genetic biodiversity heritage and is registered in the ‘Sistema Nacional de Gestão do Patrimônio Genético e do Conhecimento Tradicional Associado’ (SisGen #A1886D5).
