# Supplementary material for: Over 400 food resources from Brazil: evidence-based records of wild edible mushrooms
Source: IMA Fungus. 2024 Dec 13;15:40. doi: 10.1186/s43008-024-00171-8 (PMC11639120; doi:10.1186/s43008-024-00171-8)
Supplement: Supplementary file 1 — Additional file 1 [file 43008_2024_171_MOESM1_ESM.docx]

**Supplementary Information 2**

List of mushroom taxa, placed in categories (E1, E2, E3, P) and indicating the Final edibility status (FES) as proposed by Li et al. (2021)

E1: Edible, confirmed; E2: Edible, confirmed but with conditions; E3: Edible, unconfirmed; P: Poisonous; U: Unconfirmed status.

| **Taxon** | **E1** | **E2** | **E3** | **P** | **Total reports** | **FES** | **References** |
| --- | --- | --- | --- | --- | --- | --- | --- |
| *Amanita dulciodora* | 0 | 0 | 1 | 0 | 1 | E3 | Nascimento, C.C. (pers. obs.) |
| *Auricularia brasiliana* | 1 | 0 | 1 | 0 | 2 | E1 | Santos et al. 2023; Drewinski, M.P. (pers. obs.) |
| *Auricularia tremellosa* | 3 | 0 | 0 | 0 | 3 | E1 | Ríos-García et al. 2022; Vargas-Isla et al. 2024; Drewinski, M.P. (pers. obs.) |
| *Chlorophyllum molybdites** | 5 | 0 | 1 | 21 | 27 | U | Lincoff and Mitchel 1977; Pegler and Piearce 1980; Villarreal and Perez-Moreno 1989; Arora 1991; Rammeloo and Walleyn 1993; Hongo and Izawa 1994; Walleyn and Rammeloo 1994; Chang and Mao 1995; Laessoe and Del-Conte 1996; Mao 2000; Härkönen et al. 2003; Meijer et al. 2007; Yamada et al. 2012; Chen et al. 2014; Bijeesh et al. 2017 |
| *Cookiena speciosa* | 3 | 0 | 0 | 0 | 3 | E1 | Milenge Kamalebo et al. 2018; Ruan-Soto et al. 2021; De León 2022 |
| *Filoboletus gracilis* | 2 | 0 | 0 | 0 | 2 | E1 | Albert and Milliken 2009; Trierveiler-Pereira 2024 |
| *Gyroporus austrobrasiliensis* | 1 | 0 | 0 | 0 | 1 | E1 | Magnago, A.C (pers. obs.) |
| *Inonotus obliquus** | 4 | 0 | 1 | 0 | 4 | E1 | Saar 1991; Hongo and Izawa 1994; Perevedentseva 2013; Zhang et al. 2015; Stojkovic et al. 2019 |
| *Lactarius hepaticus* | 2 | 0 | 0 | 0 | 2 | E1 | Benbrahim et al. 2006; Nascimento, C.C. (pers. obs.) |
| *Lactarius taedae** | 0 | 0 | 1 | 0 | 1 | E3 | Sulzbacher, M.A. (pers. comm.) |
| *Marasmiellus cubensis* | 1 | 0 | 0 | 0 | 1 | E1 | Albert and Milliken 2009 |
| *Panus tephroleucus* | 1 | 0 | 0 | 0 | 1 | E1 | Albert and Milliken 2009 |
| *Phlebopus beniensis* | 3 | 0 | 0 | 0 | 3 | E1 | Prado-Elias et al. 2022; Campi et al. 2023; Trierveiler-Pereira 2024 |
| *Pleurotus magnificus* | 2 | 0 | 0 | 0 | 2 | E1 | Timm 2021; Bittencourt et al. 2024 |
| *Polyporus pes-simiae** | 0 | 0 | 1 | 0 | 1 | E3 | Fidalgo 1968 |
| *Pseudohydnum viridimontanum* | 1 | 0 | 0 | 0 | 1 | E1 | Coelho-Nascimento et al. 2024 |
| *Stropharia coronilla** | 4 | 0 | 1 | 4 | 9 | U | Lincoff and Mitchel 1977; Aroche et al. 1984; Villarreal and Perez-Moreno 1989; Gerhardt 1994; Chang and Mao 1995; Laessoe and Del-Conte 1996; Mao 2000; Gerhardt 2001; Montoya-Esquivel et al. 2001 |
| *Trechispora thelephora* | 0 | 0 | 1 | 0 | 1 | E3 | Bononi 1984 |

* FES different from that proposed by Li et al. (2021)

**References**

Albert B, Milliken W (2009) *Urihi A: a terra-floresta Yanomami*. São Paulo, SP/ Brazil: Instituto Socioambiental.

Aroche RM, Cifuentes J, Lorea F, Fuentes P, Bonavides J, Galicia H, Menéndez E, Aguilar O, Valenzuela V (1984) Poisonous and edible macromycetes in a communal region of the Valle de Mexico, I. Boletin de la Sociedad Mexicana de Micología 19: 291–318.

Arora D (1991) *All that the rain promises and more. A hip pocket guide to Western mushrooms.* Berkeley, CA: Ten Speed Press.

Benbrahim M, Denaix L, Thomas A-L, Balet J, Carnus J-M (2006) Metal concentrations in edible mushrooms following municipal sludge application on forest land. Environmental Pollution 144: 847–854. <https://doi.org/10.1016/j.envpol.2006.02.014>

Bijeesh C, Vrinda K, Pradeep C (2017) Mushroom poisoning by *Chlorophyllum molybdites* in Kerala. Journal of Mycopathological Research 54: 477–483.

Bittencourt F, Drewinski MP, Menolli Jr N, Drechsler-Santos ER (2024) Rare *Pleurotus* species with veiled basidiomata from the Neotropics: neotypification of *Pleurotus magnificus* and epityfication of *Pleurotus rickii*. Phytotaxa (in press).

Bononi VL (1984) Basidiomicetos do Parque Estadual da Ilha do Cardoso. IV. Adições às famílias Hymenochaetaceae, Stereaceae e Thelephoraceae. Rickia 11: 43–52.

Campi M, Mancuello C, Maubet Y, Cristaldo E, Veloso B, Ferreira F, … Robledo G (2023) Biochemical, nutritional, and toxicological properties of the edible species *Phlebopus beniensis* with ethnomycological notes from Paraguay. Brazilian Journal of Food Technology 26: e2022126. <https://doi.org/10.1590/1981-6723.12622>

Chang S-T, Mao XL (1995) *Hong Kong mushrooms*. Hong Kong: The Chinese University of Hong Kong Press.

Chen Z, Ping Z, Zhang Z (2014) Investigation and analysis of 102 mushroom poisoning cases in Southern China from 1994 to 2012. Fungal Diversity 64: 123–131. <https://doi.org/10.1007/s13225-013-0260-7>

Coelho-Nascimento C, Zabin DA, Silva-Filho AGDS, Drewinski MP, Alves-Silva G, Kossmann T, Titton M, Drechsler-Santos ER, Menolli Jr N (2024) Unroughing the cat’s tongue mushrooms: Four new species of *Pseudohydnum* from Brazil based on morphological and molecular phylogenetic evidence. Mycologia 116(4): 1–29. <https://doi.org/10.1080/00275514.2024.2363141>

De León JA (2022) ¿Etnomicología en Panamá? Algunos usos tradicionales de los hongos en nuestro país. In Vega et al. Biodiversidad, Sostenibilidad, desarrollo Económico y Social de la Región Occidental de Panamá, David, Panamá, pp. 79–86.

Fidalgo MEPK (1968) Contribution to the fungi of Mato Grosso, Brazil. Rickia 3: 171–219.

Gerhardt E (1994) *Przewodnik Grzyby*. Warsaw, Poland: Multico.

Gerhardt E (2001) *Der grosse BLV Pilzführer für unterwegs*. Munich: BLV.

Härkönen M, Niemelä T, Mwasumbi L (2003) *Tanzanian mushrooms. Edible, harmful and other fungi*. Helsinki: Botanical Museum, Finnish Museum of Natural History.

Hongo T, Izawa M (1994) *Kinoko*. Yama-Kei field books.Vol.10. Tokyo: Yamakei Publishers.

Laessoe T, Del-Conte A (1996) *The mushroom book*. London: Dorling Kindersley.

Li H, Tian Y, Menolli Jr. N, Ye L, Karunarathna SC, Pérez-Moreno J, … Mortimer PE (2021) Reviewing the world's edible mushroom species: A new evidence‐based classification system. Comprehensive Reviews in Food Science and Food Safety 20(2): 1982–2014. <https://doi.org/10.1111/1541-4337.12708>

Lincoff G, Mitchel DH (1977) *Toxic and hallucinogenic mushroom poisoning. A handbook for physicians and mushroom hunters*. New York: Van Nostrand Reinhold Company.

Mao XL (2000) *The Macrofungi of China*. Zhengzhou, Henan: Henan Science and Technology Press.

Meijer AAR, Amazonas MALA, Rubio GBG, Curial RM (2007) Incidences of poisonings due to *Chlorophyllum molybdites* in the state of Paraná, Brazil. Brazilian Archives of Biology and Technology 50: 479–488. <https://doi.org/10.1590/S1516-89132007000300014>

Milenge Kamalebo H, Nshimba Seya Wa Malale H, Masumbuko Ndabaga C, Degreef J, De Kesel A (2018) Uses and importance of wild fungi: traditional knowledge from the Tshopo province in the Democratic Republic of the Congo. Journal of Ethnobiology Ethnomedicine 14: 1–12. <https://doi.org/10.1186/s13002-017-0203-6>

Montoya-Esquivel A, Estrada-Torres A, Kong A, Juarez-Sanchez L (2001) Commercialization of wild mushrooms during market days of Tlaxcala, Mexico. Micologia Aplicada International 13: 31–40.

Pegler DN, Piearce GD (1980) The edible mushrooms of Zambia. Kew Bulletin 35: 475–491. <https://doi.org/10.2307/4110017>

Perevedentseva L (2013) Use of wild-growing mushrooms for therapeutic purposes in the Perm Territory, Russia. Journal of Environmental Science and Engineering A 2: 236–242.

Prado-Elias A, Almeida NS, Ruan-Soto F, Baltazar JM, Trierveiler-Pereira L (2022) *Phlebopus beniensis* (Singer & Digilo) Heinem. & Rammeloo (Boletinellaceae, Basidiomycota, Fungi): novo registro para o Estado de São Paulo, Brasil e notas etnomicológicas. Hoehnea 49: e532021. <https://doi.org/10.1590/2236-8906-53/2021>

Rammeloo J, Walleyn R (1993) The edible fungi of Africa south of the Sahara: A literature survey. Scripta Botanica Belgica 5: 1–62.

Ríos-Garcia U, Martínez-Reyes M, Carrera-Martínez A, Hernández-Santiago F, Díaz-Aguilar I, Leyva-López JC, Cházarez-Vargas F, Pérez-Moreno J (2022) Nomenclatura y vocabulario del recurso micológico con importancia biocultural de la cultura mazateca em Oaxaca, México. Scientia Fungorum 53: e1424. <https://doi.org/10.33885/sf.2022.53.1424>

Ruan-Soto F, Domínguez-Gutiérrez M, Pérez-Ramírez L, Cifuentes J (2021) Etnomicología de los lacandones de Nahá, Metzabok y Lacanjá-Chansayab, Chiapas, México. Ciencias Sociales y Humanidades 8(1): 24–42. <https://doi.org/10.36829/63CHS.v8i1.1112>

Saar M (1991) Fungi in Khanty folk medicine. Journal of Ethnopharmacology 31(2): 175–179. <https://doi.org/10.1016/0378-8741(91)90003-V>

Santos DC, Oliveira AM, Alvarenga RLM, Gibertoni TB (2023) How climate will change the potential distribution of two wood-decaying fungi? Acta Botanica Brasilica 37: e20230021. <https://doi.org/10.1590/1677-941X-ABB-2023-0021>

Stojkovic D, Smiljkovic M, Ciric A, Glamoclija J, Van Griensven L, Ferreira IC, Sokovic M (2019) An insight into antidiabetic properties of six medicinal and edible mushrooms: Inhibition of α-amylase and α-glucosidase linked to type-2 diabetes. South African Journal of Botany 120: 100–103. <https://doi.org/10.1016/j.sajb.2018.01.007>

Timm JM (2021) *Primavera Fungi. Guia de Fungos do Sul do Brasil.* 2 ed. Via Sapiens, Porto Alegre. 384p.

Trierveiler-Pereira L (2024) *FANCS de Angatuba. Fungos alimentícios não convencionais de Angatuba e região.* Edição Impressa. Angatuba, SP.

Vargas-Isla R, Leite L, Cabral T, Oliveira J, Pereira R, Ishikawa N (2024) *Série Mycelia: álbum de cogumelos para micoturismo: volume 3*. Manaus, AM.

Villarreal L, Perez-Moreno J (1989) Los hongos comestibles silvestres de Mexico, un enfoque integral. Micologia Neotropica Aplicada 2: 77–114.

Walleyn R, Rammeloo J (1994) The poisonous and useful fungi of Africa south of the Sahara: A literature survey. Scripta Botanica Belgica 6: 1–56.

Yamada M, Tokumitsu N, Saikawa Y, Nakata M, Asano J, Miyairi K, Okuno T, Konno K, Hashimoto K (2012) Molybdophyllysin, a toxic metalloendopeptidase from the tropical toadstool, *Chlorophyllum* *molybdites*. Bioorganic & Medicinal Chemistry 20: 6583–6588. <https://doi.org/10.1016/j.bmc.2012.09.036>

Zhang N, Chen H, Zhang Y, Xing L, Li S, Wang X, Sun Z (2015) Chemical composition and antioxidant properties of five edible Hymenomycetes mushrooms. International Journal of Food Science & Technology 50(2): 465–471. <https://doi.org/10.1111/ijfs.12642>
