# Supplementary material for: Over 400 food resources from Brazil: evidence-based records of wild edible mushrooms
Source: IMA Fungus. 2024 Dec 13;15:40. doi: 10.1186/s43008-024-00171-8 (PMC11639120; doi:10.1186/s43008-024-00171-8)
Supplement: Supplementary file 2 — Additional file 2 [file 43008_2024_171_MOESM2_ESM.docx]

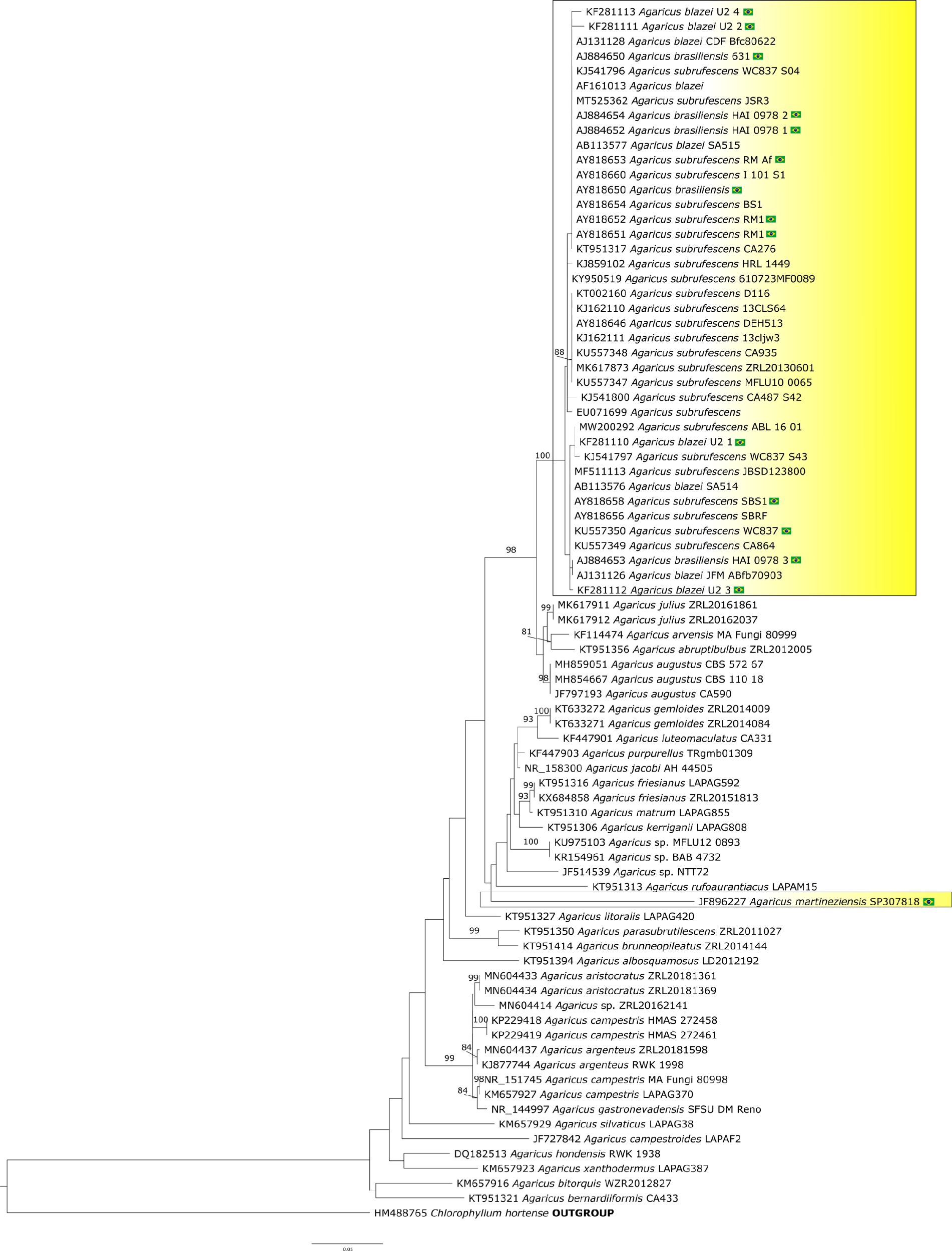


Figure S1. Maximum Likelihood (ML) tree of *Agaricus* based on ITS data. Branches are labeled with ML bootstrap higher than 80%. The highlight in yellow represents the clade of species *Agaricus subrufescens* and *Agaricus martineziensis*.


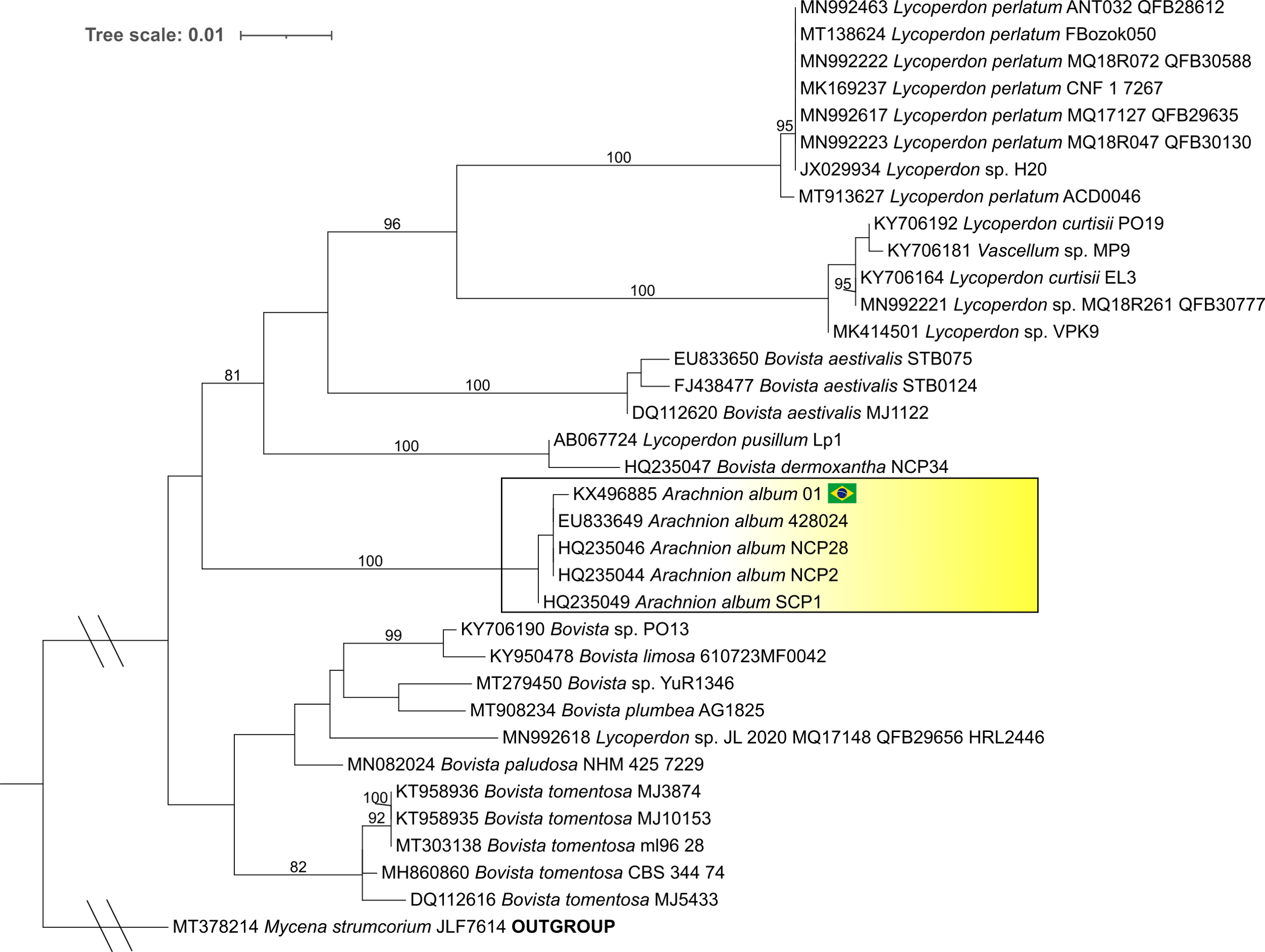


Figure S2. Maximum Likelihood (ML) tree of *Arachnion* and related genera based on ITS data. Branches are labeled with ML bootstrap higher than 80%. The highlight in yellow represents the clade of species *Arachnion album*.


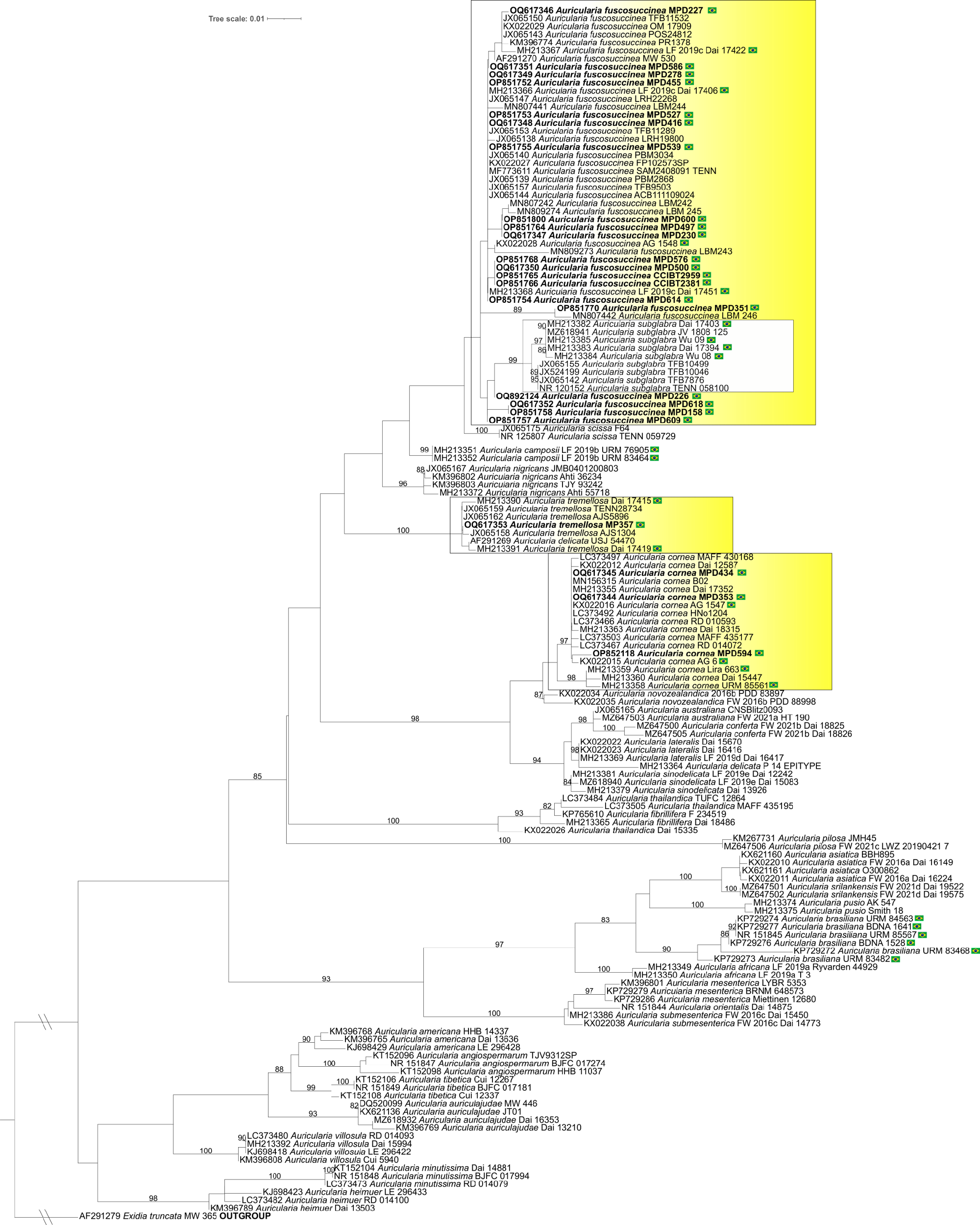


Figure S3. Maximum Likelihood (ML) tree of *Auricularia* based on ITS data. Branches are labeled with ML bootstrap higher than 80%. The highlight in yellow represents the clade of species *Auricularia cornea*, *Auricularia fuscosuccinea* complex and *Auricularia tremellosa*. The sequences in bold were generated in this work.


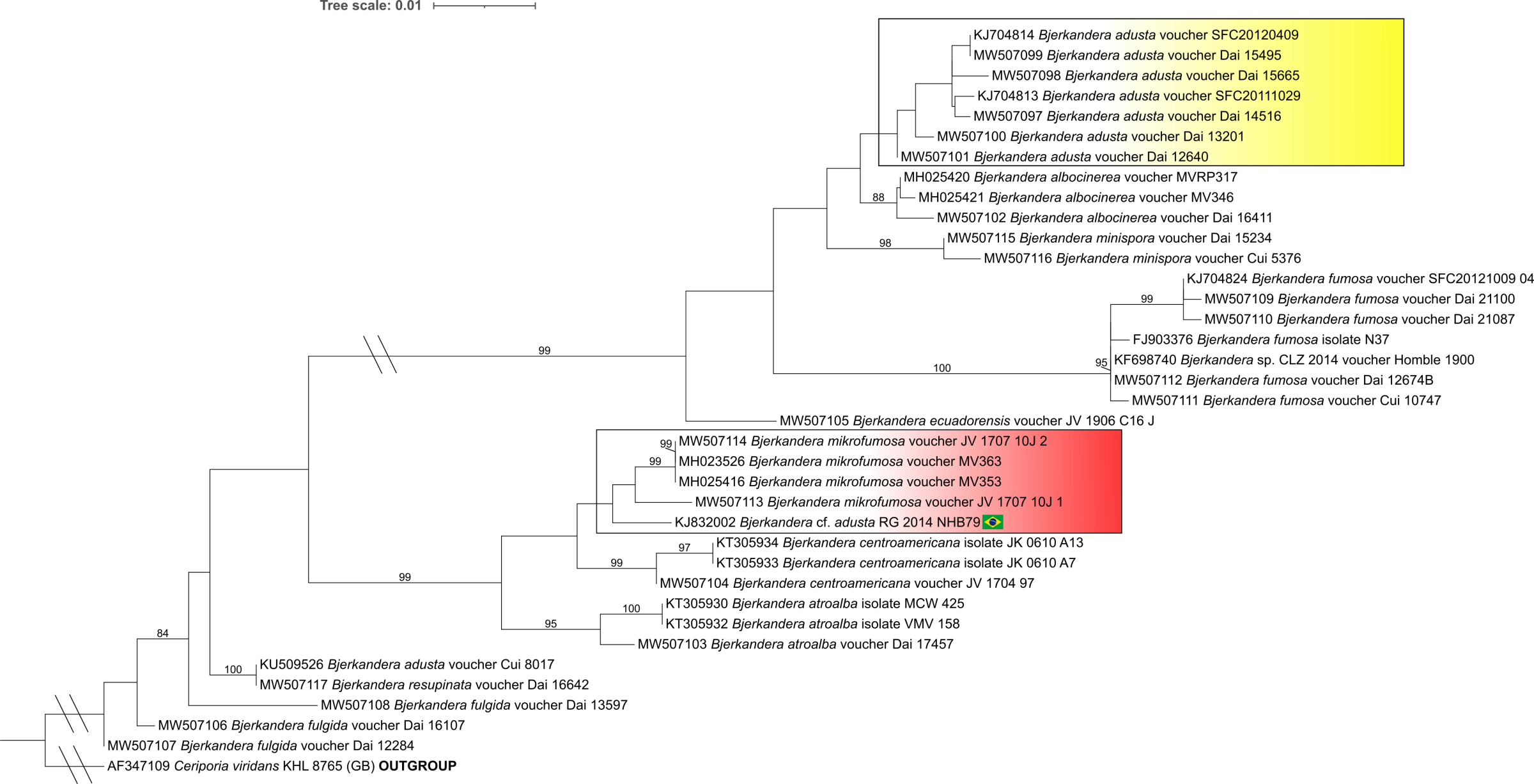


Figure S4. Maximum Likelihood (ML) tree of *Bjerkandera* based on ITS data. Branches are labeled with ML bootstrap higher than 80%. The highlight in yellow represents the clade of species *Bjerkandera adusta*. The red highlight represents the clade with the misidentified sequence.


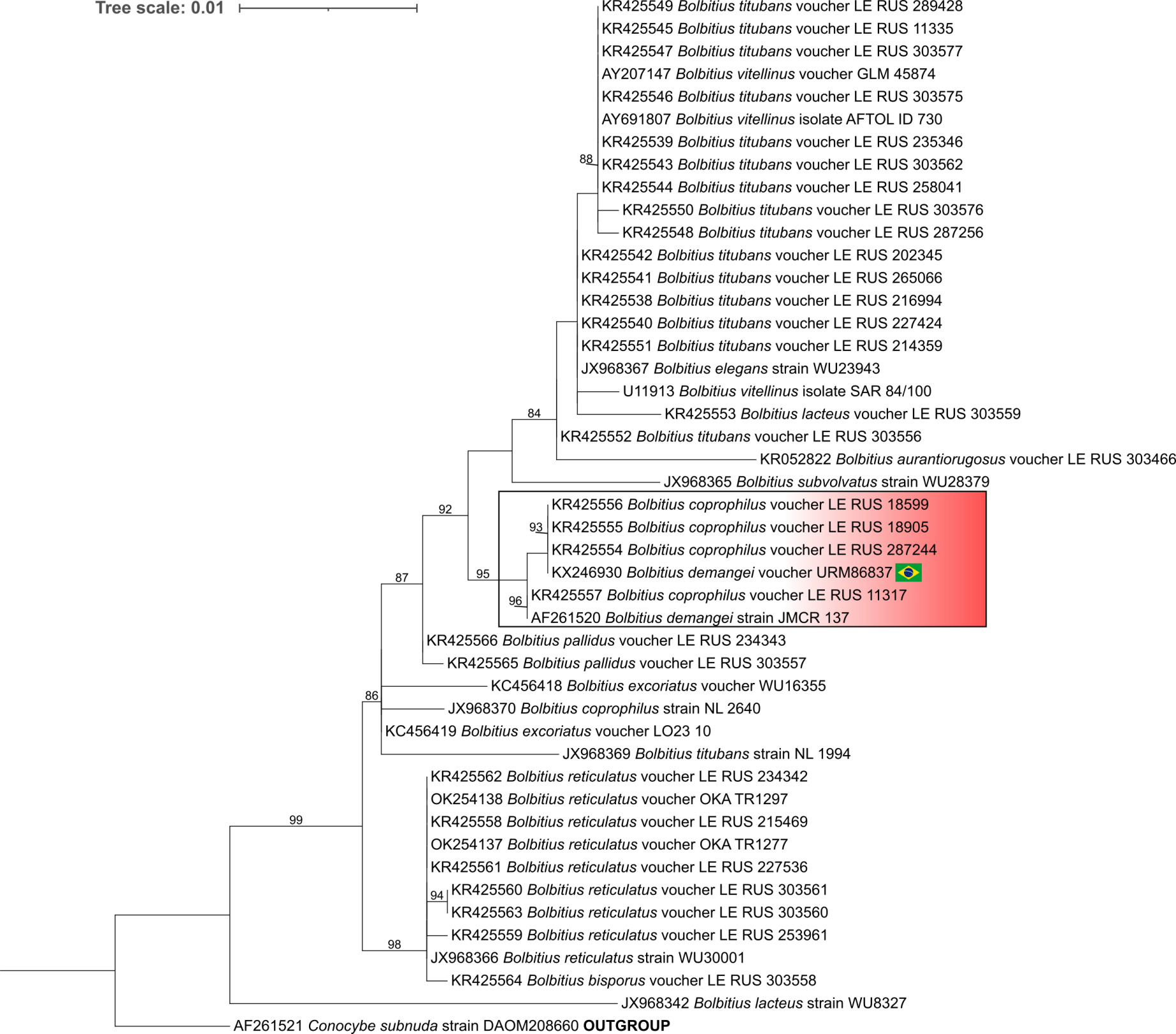


Figure S5. Maximum Likelihood (ML) tree of *Bolbitius* based on LSU data. Branches are labeled with ML bootstrap higher than 80%. The red highlight represents the clade with the unconfirmed sequence.


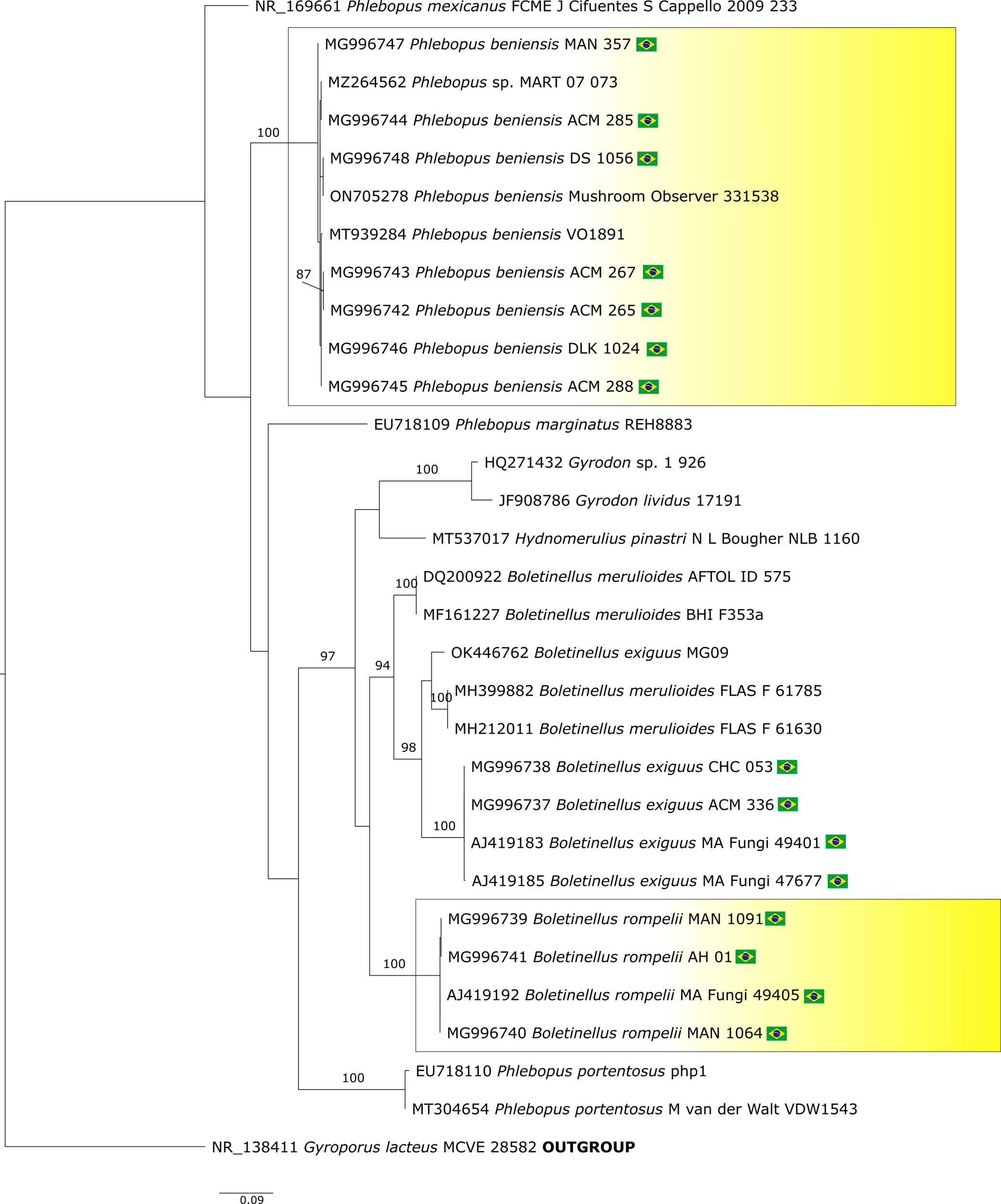


Figure S6. Maximum Likelihood (ML) tree of *Boletinellus* and allied genera based on ITS data. Branches are labeled with ML bootstrap higher than 80%. The highlight in yellow represents the clade of species *Boletinellus rompelli* and *Phlebopus beniensis.*


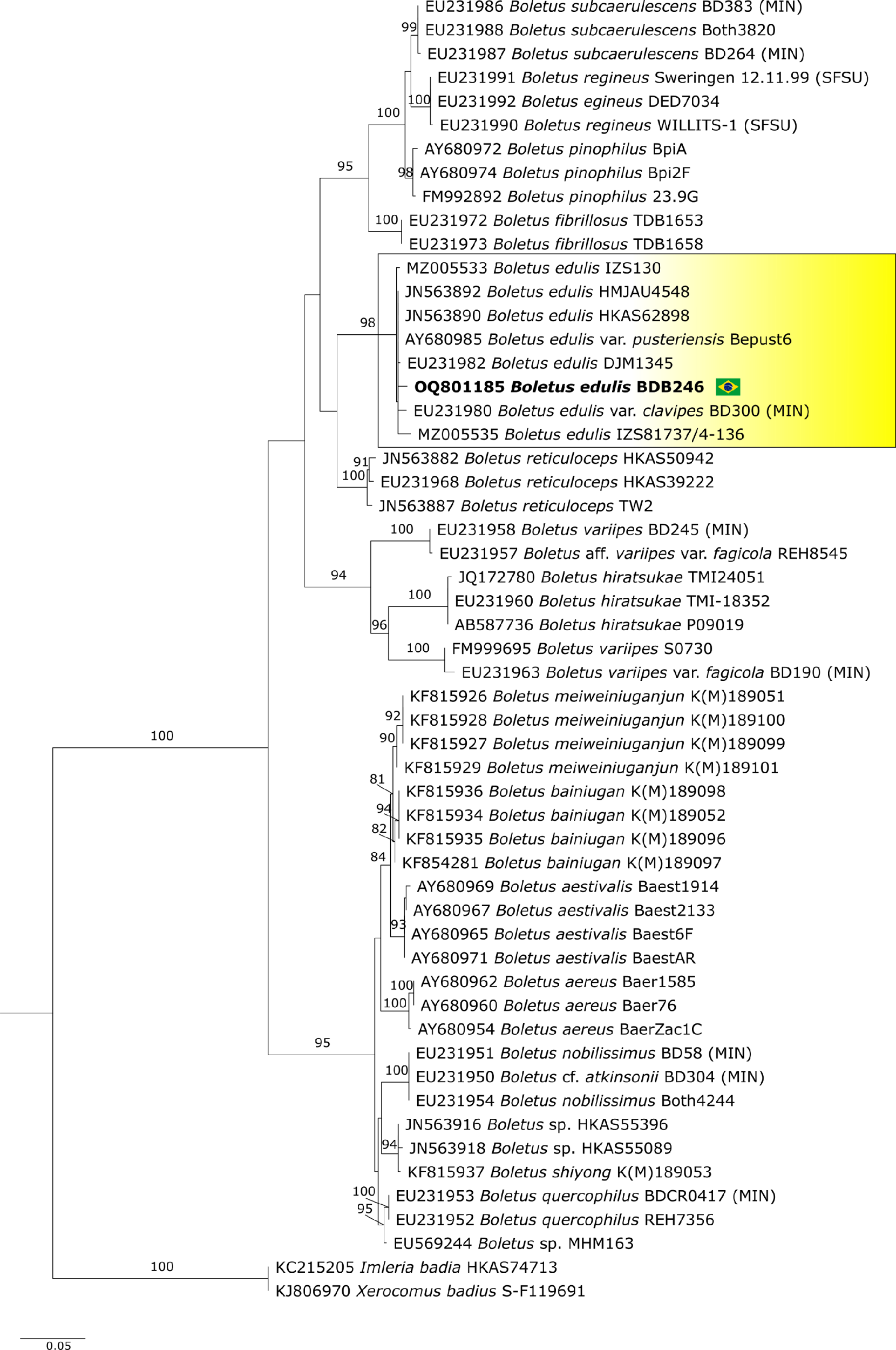


Figure S7. Maximum Likelihood (ML) tree of *Boletus* based on ITS data. Branches are labeled with ML bootstrap higher than 80%. The highlight in yellow represents the clade of species *Boletus edulis*. The sequence in bold was generated in this work.


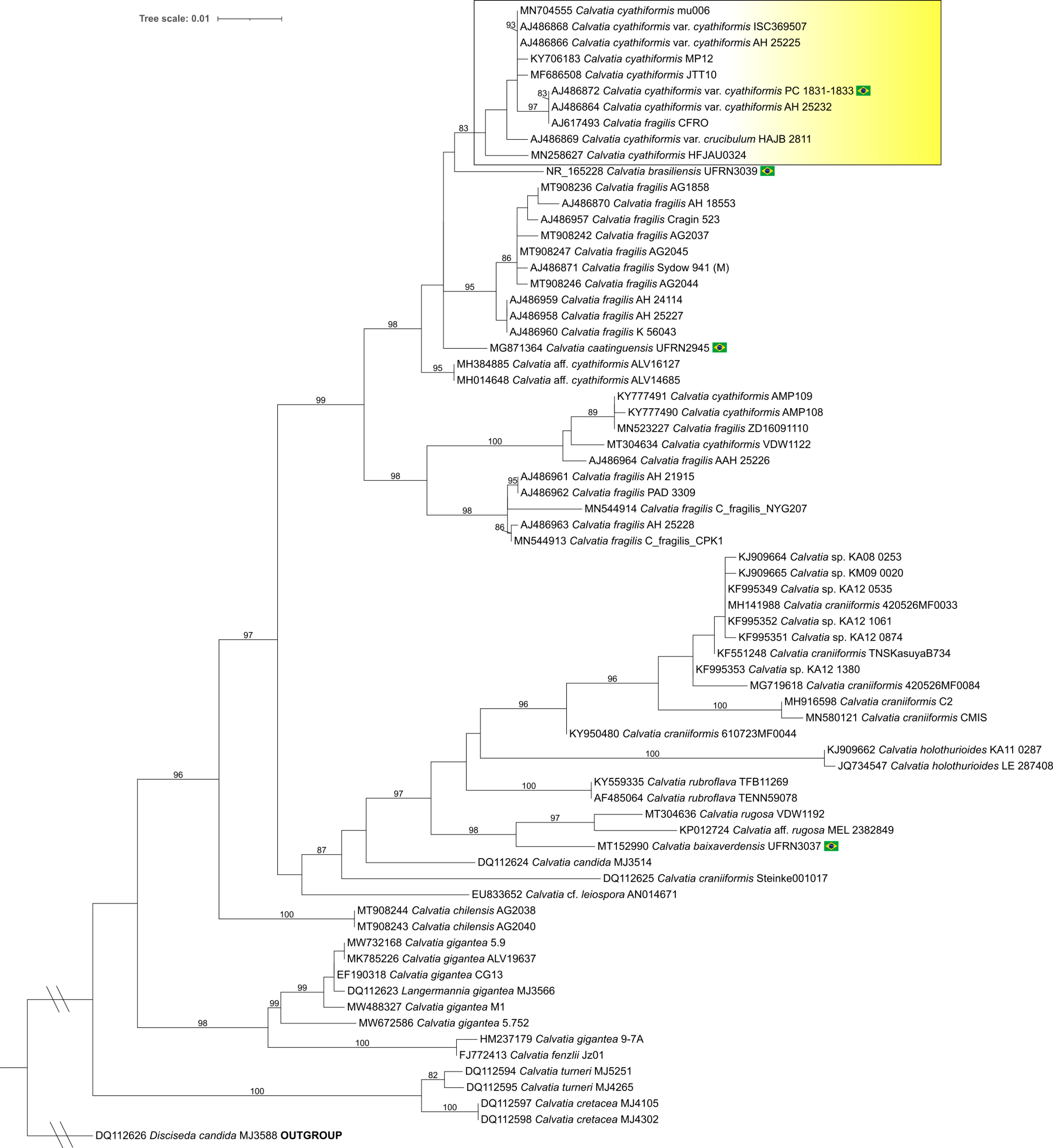


Figure S8. Maximum Likelihood (ML) tree of *Calvatia* based on ITS data. Branches are labeled with ML bootstrap higher than 80%. The highlight in yellow represents the clade of species *Calvatia cyathiformis*.


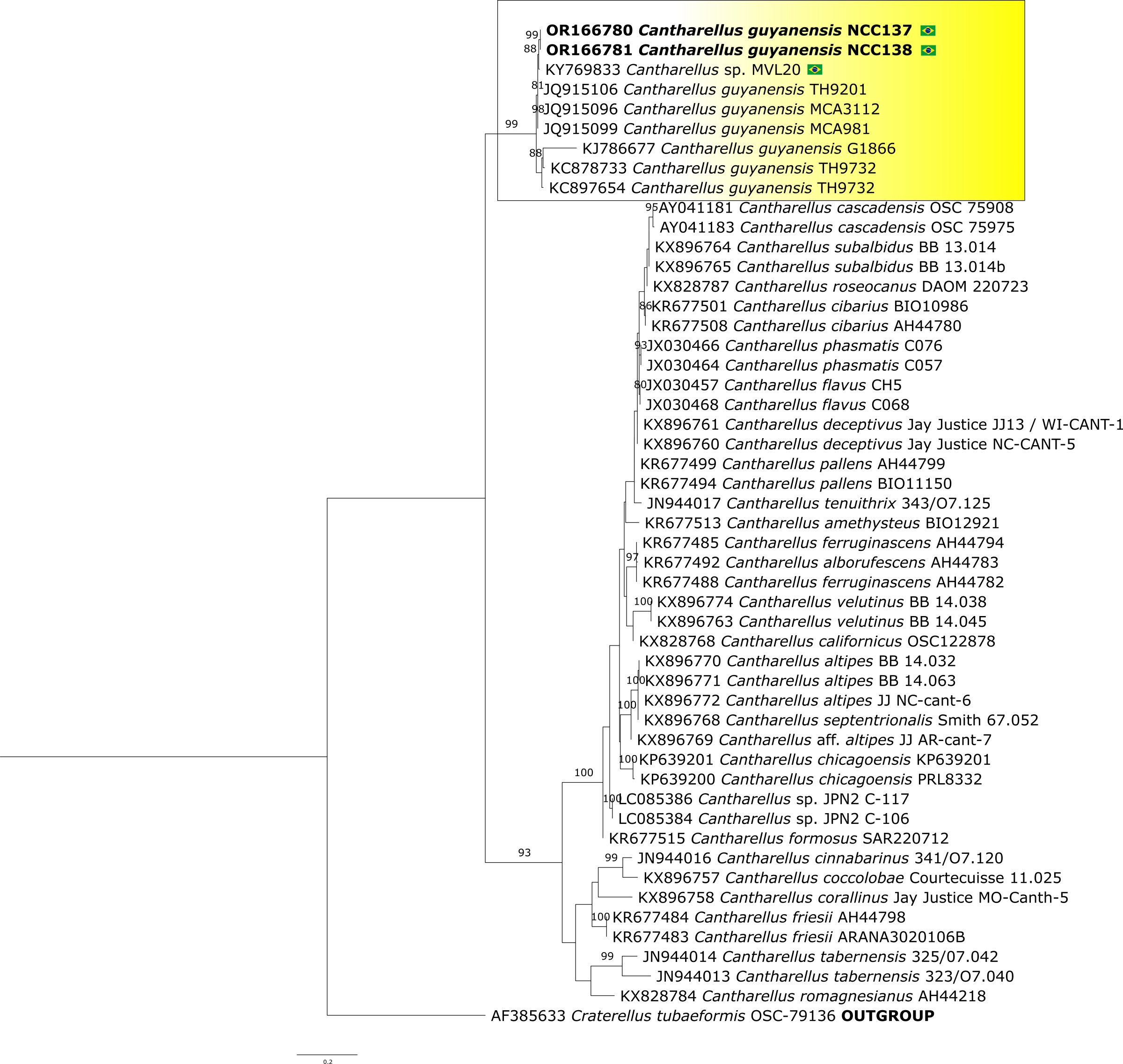


Figure S9. Maximum Likelihood (ML) tree of *Cantharellus* based on ITS data. Branches are labeled with ML bootstrap higher than 80%. The highlight in yellow represents the clade of species *Cantharellus guyanensis*. The sequences in bold were generated in this work.


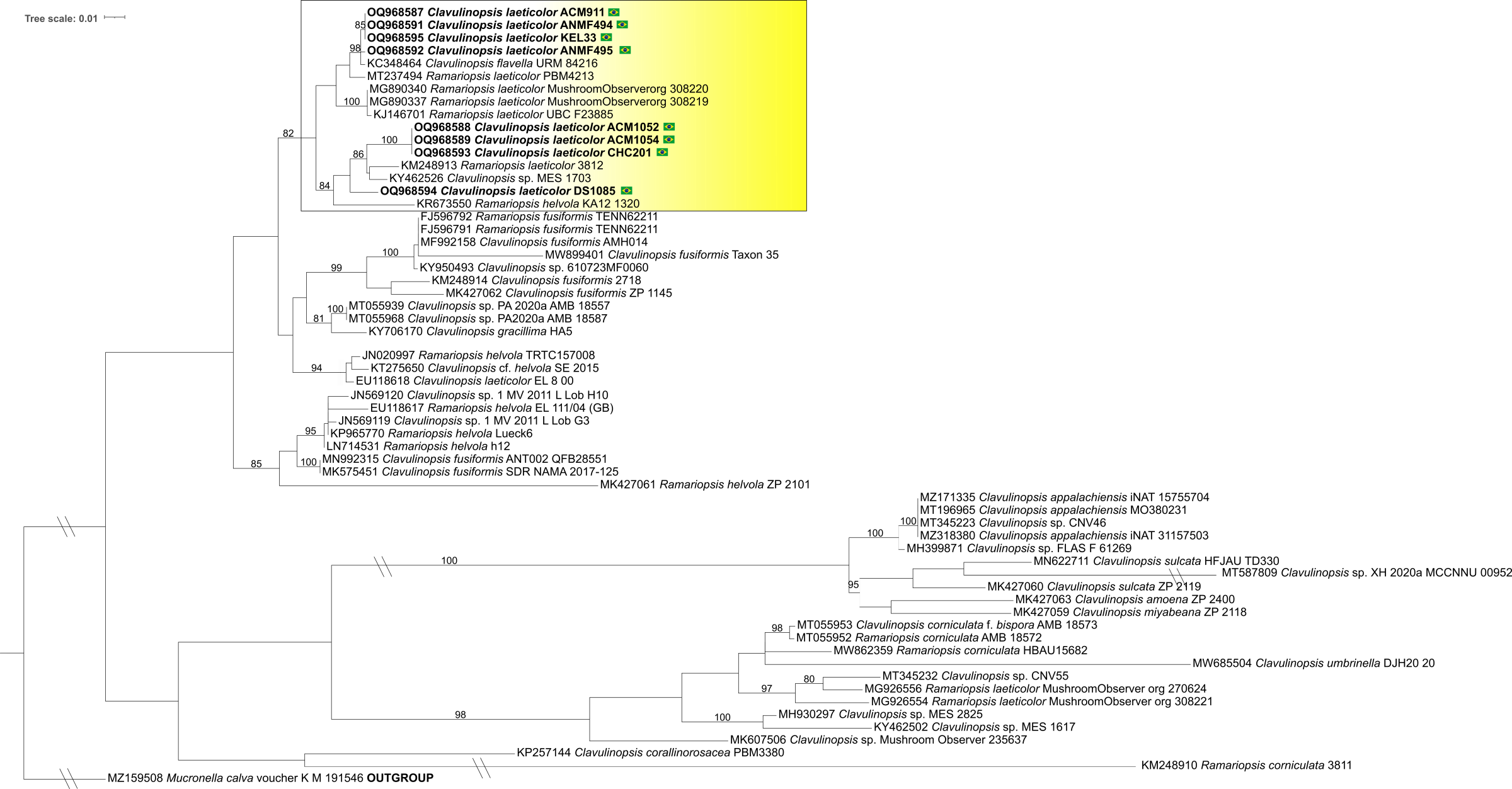


Figure S10. Maximum Likelihood (ML) tree of *Clavulinopsis* based on ITS data. Branches are labeled with ML bootstrap higher than 80%. The highlight in yellow represents the clade of species *Clavulinopsis laeticolor*. The sequences in bold were generated in this work.


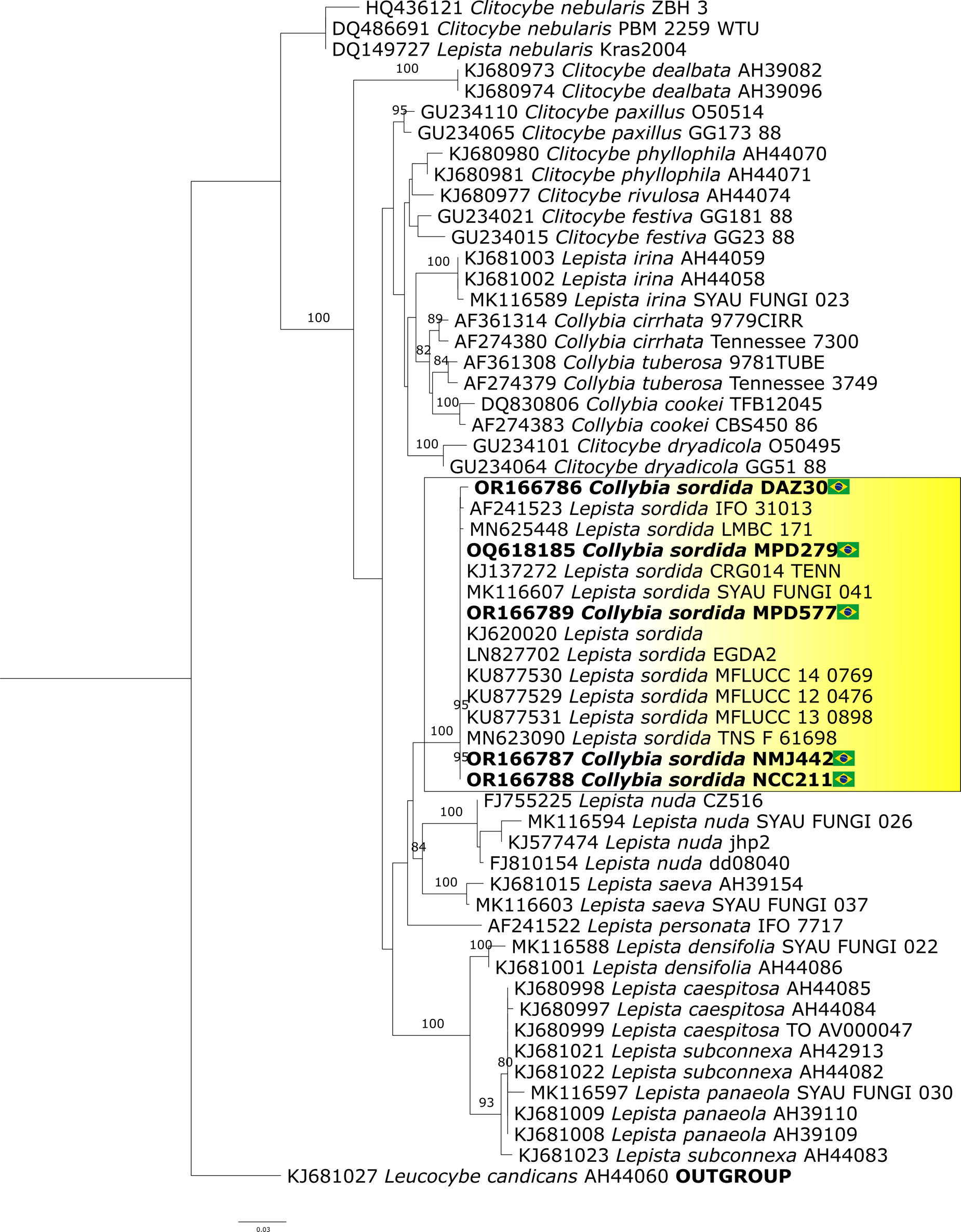


Figure S11. Maximum Likelihood (ML) tree of *Collybia* and allied genera based on ITS data. Branches are labeled with ML bootstrap higher than 80%. The highlight in yellow represents the clade of species *Collybia sordida*. The sequences in bold were generated in this work.


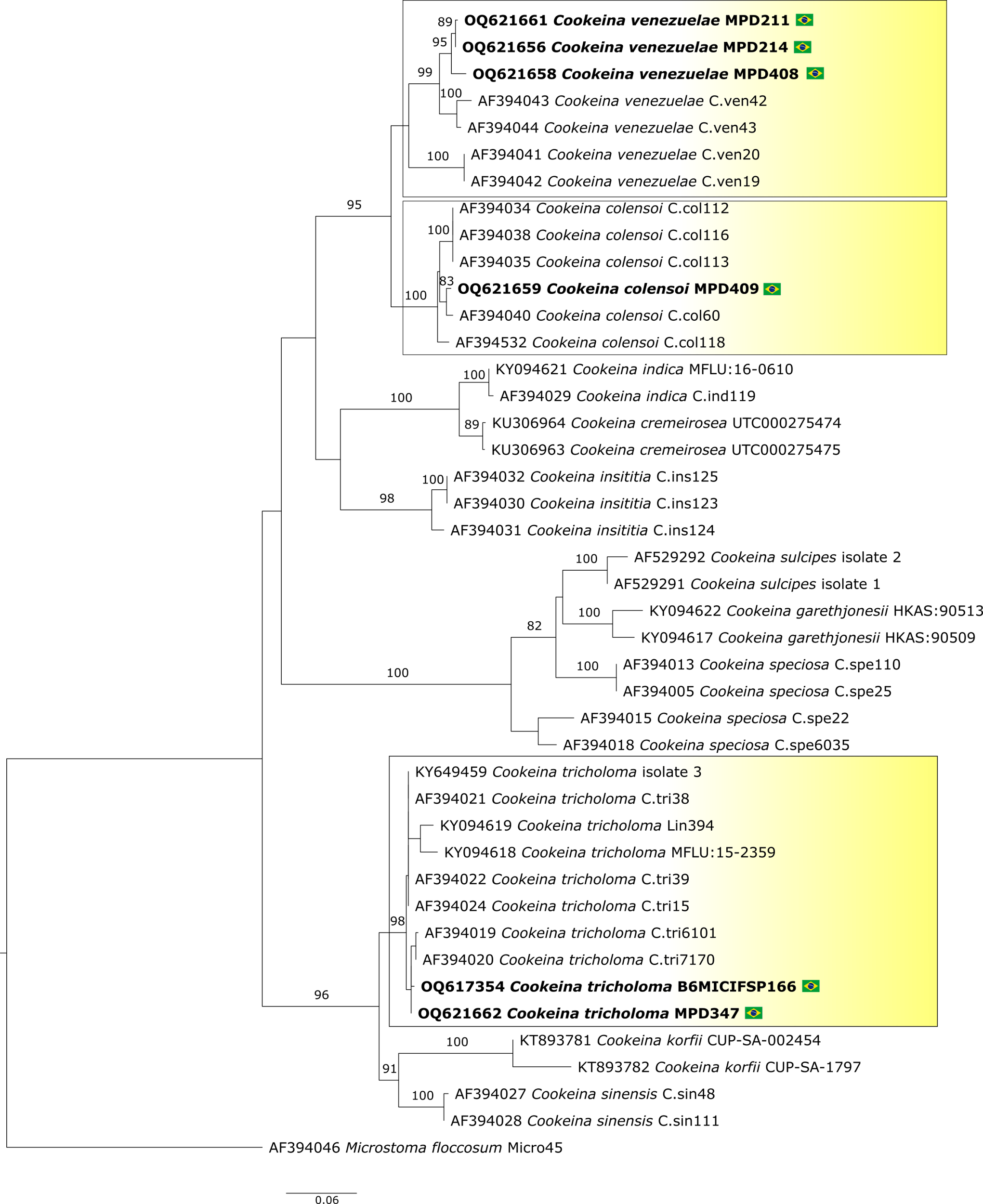


Figure S12. Maximum Likelihood (ML) tree of *Cookeina* based on ITS data. Branches are labeled with ML bootstrap higher than 80%. The highlight in yellow represents the clade of species *Cookeina colensoi*, *Cookeina tricholoma*, and *Cookeina venezuelae*. The sequences in bold were generated in this work.


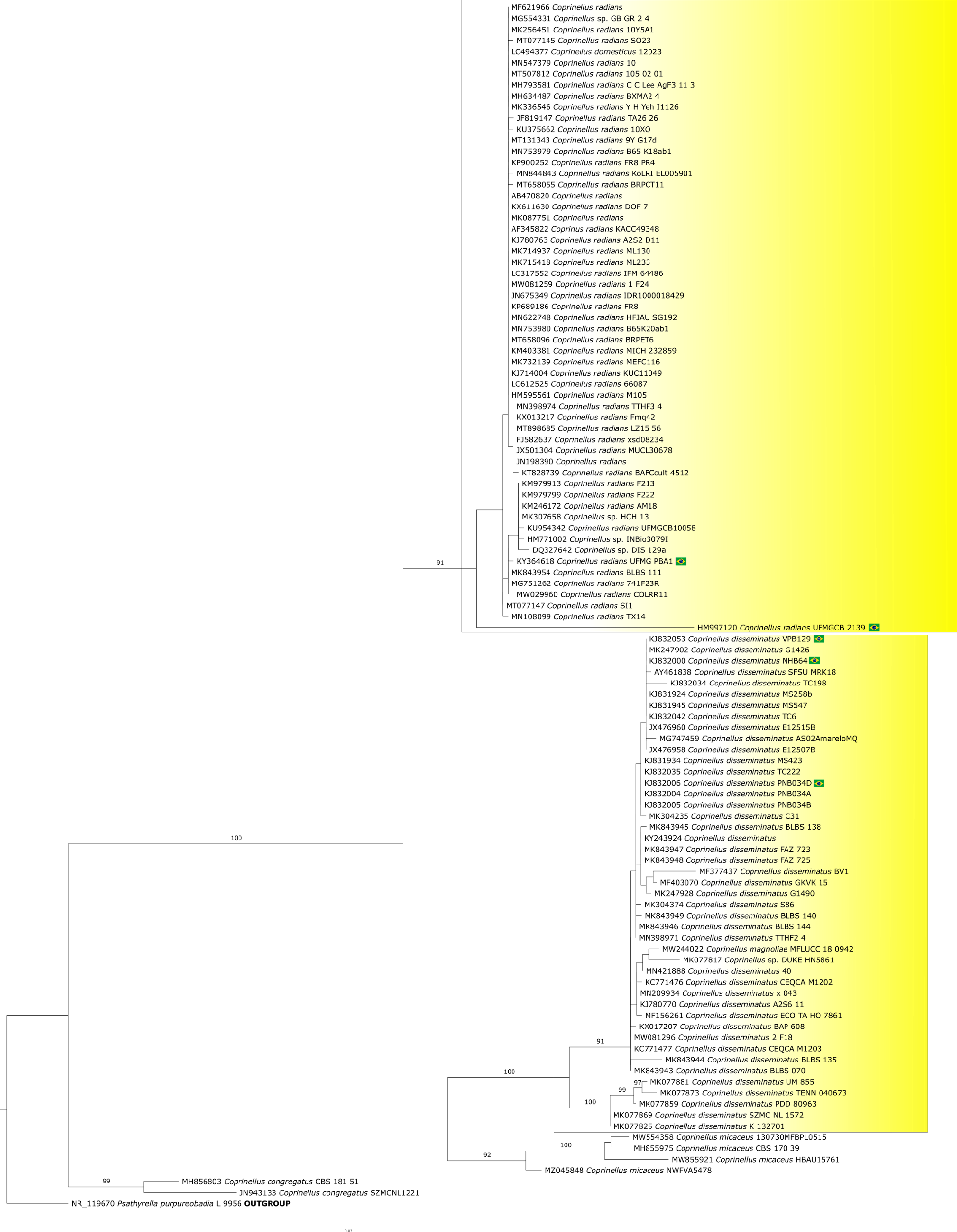


Figure S13. Maximum Likelihood (ML) tree of *Coprinellus* based on ITS data. Branches are labeled with ML bootstrap higher than 80%. The highlight in yellow represents the clade of species *Coprinellus disseminatus* and *Coprinellus radians*.


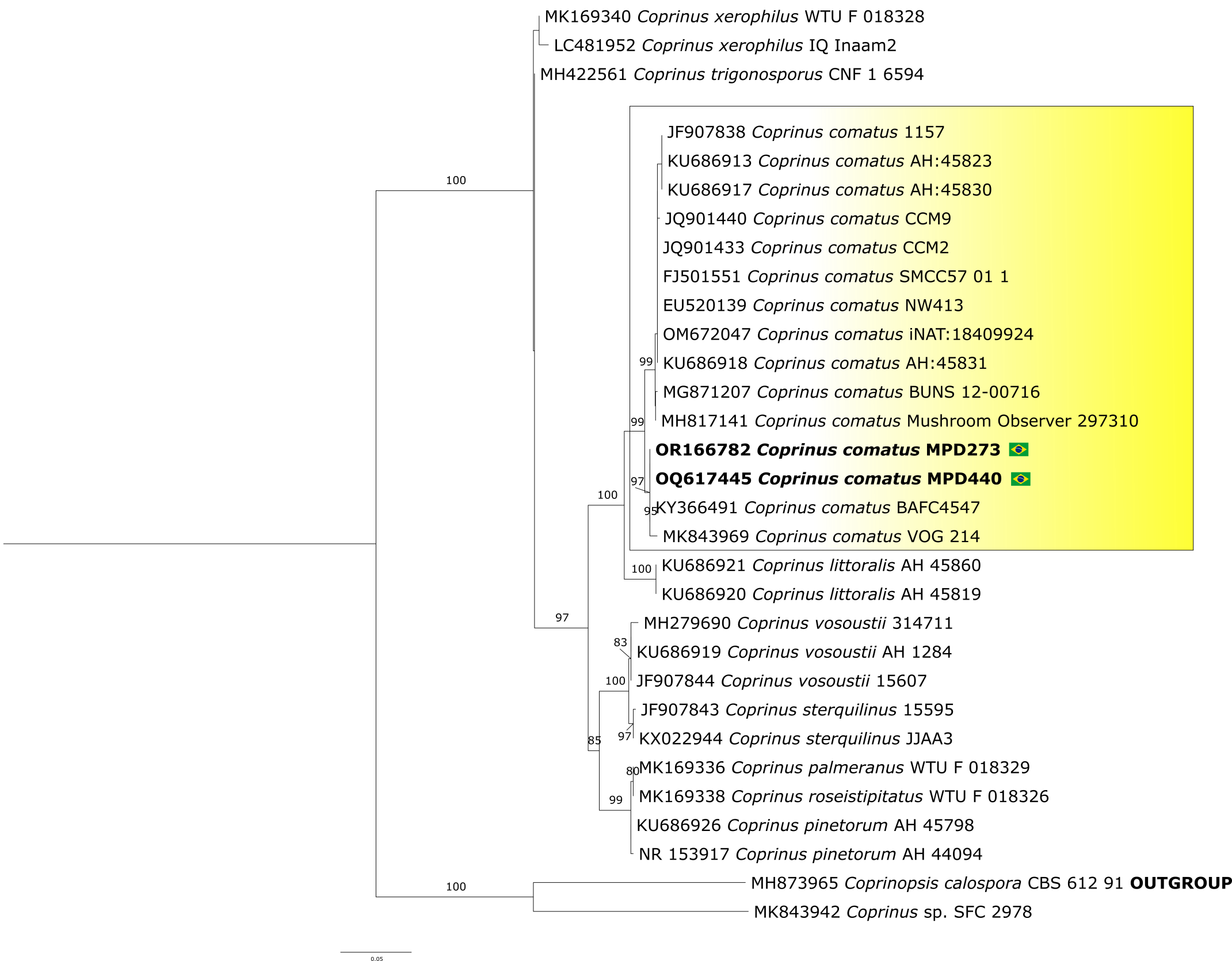


Figure S14. Maximum Likelihood (ML) tree of *Coprinus* based on ITS data. Branches are labeled with ML bootstrap higher than 80%. The highlight in yellow represents the clade of species *Coprinus comatus*. The sequences in bold were generated in this work.


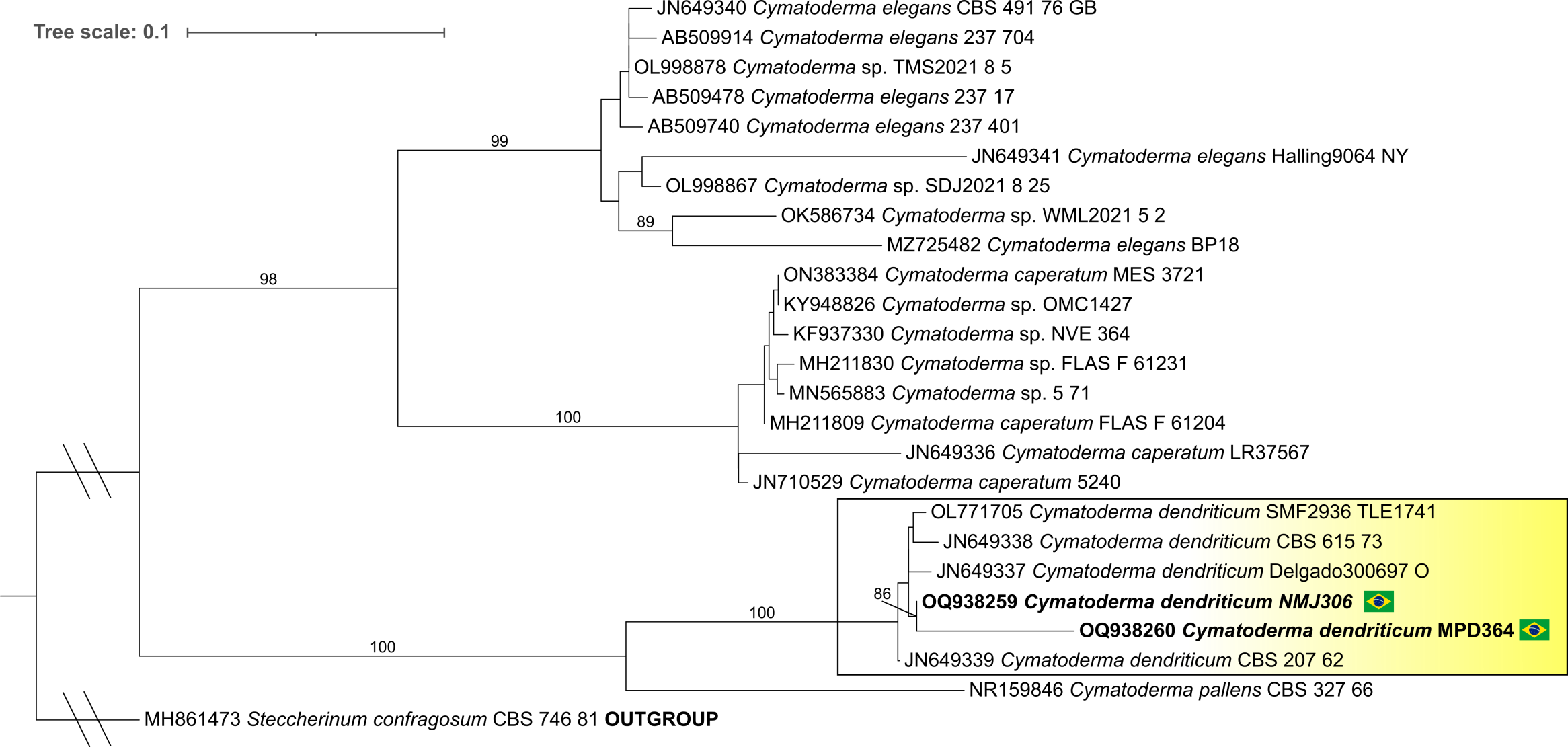


Figure S15. Maximum Likelihood (ML) tree of *Cymatoderma* based on ITS data. Branches are labeled with ML bootstrap higher than 80%. The highlight in yellow represents the clade of species *Cymatoderma dendriticum*. The sequences in bold were generated in this work.


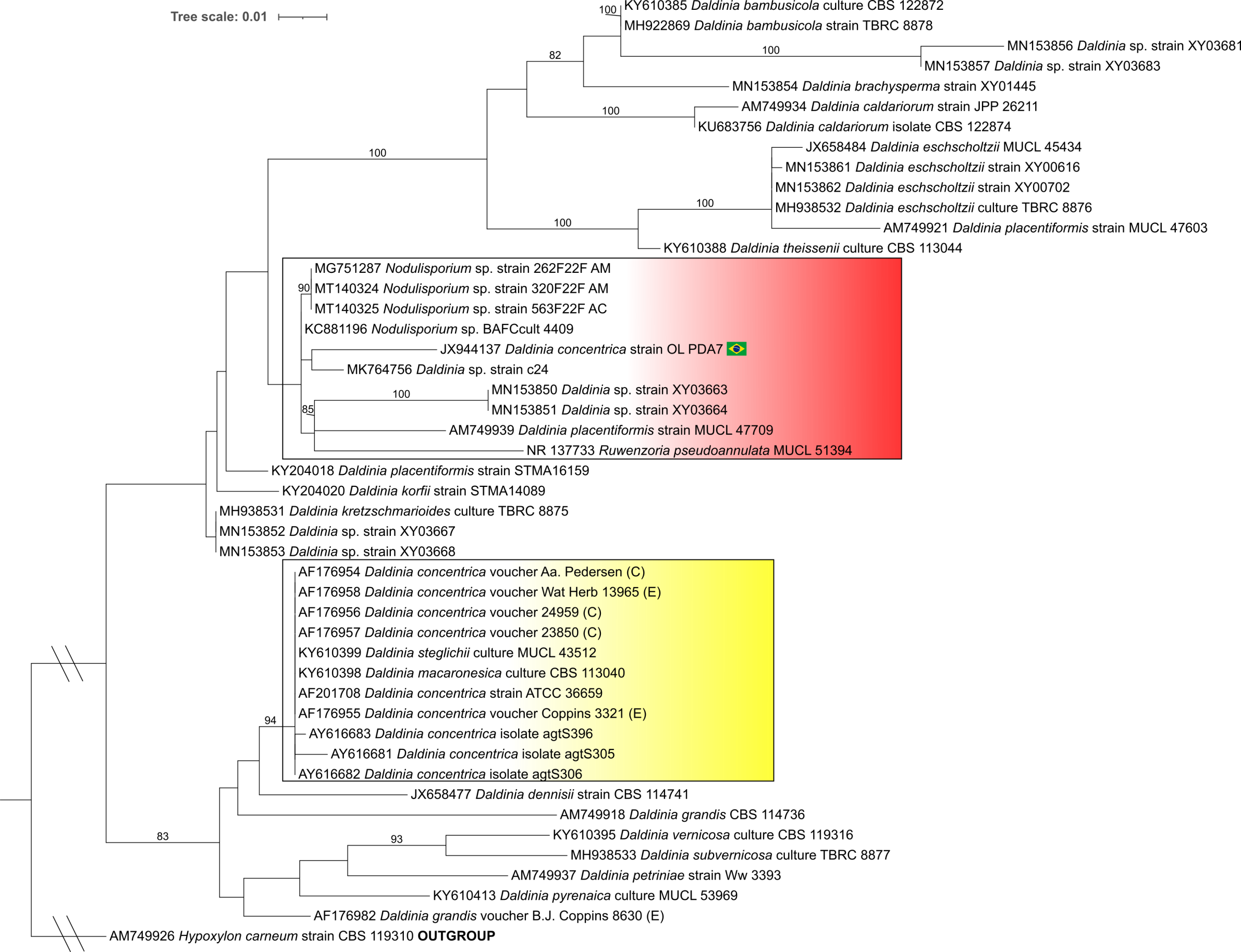


Figure S16. Maximum Likelihood (ML) tree of *Daldinia* based on ITS data. Branches are labeled with ML bootstrap higher than 80%. The highlight in yellow represents the clade of species *Daldinia concentrica*. The red highlight represents the clade with a misidentified sequence.


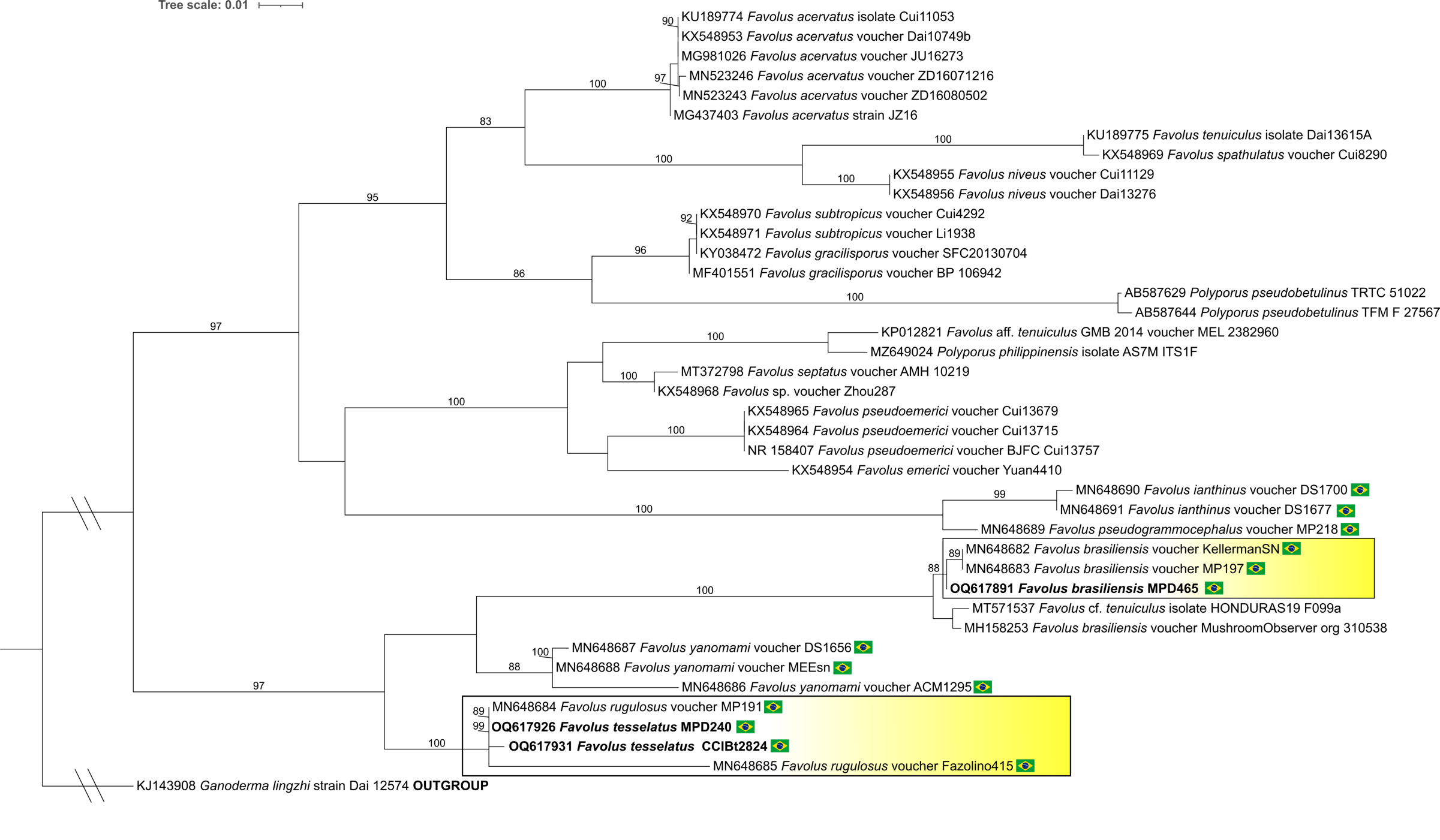


Figure S17. Maximum Likelihood (ML) tree of *Favolus* based on ITS data. Branches are labeled with ML bootstrap higher than 80%. The highlight in yellow represents the clade of species *Favolus brasiliensis* and *Favolus tesselatus*. The sequences in bold were generated in this work.


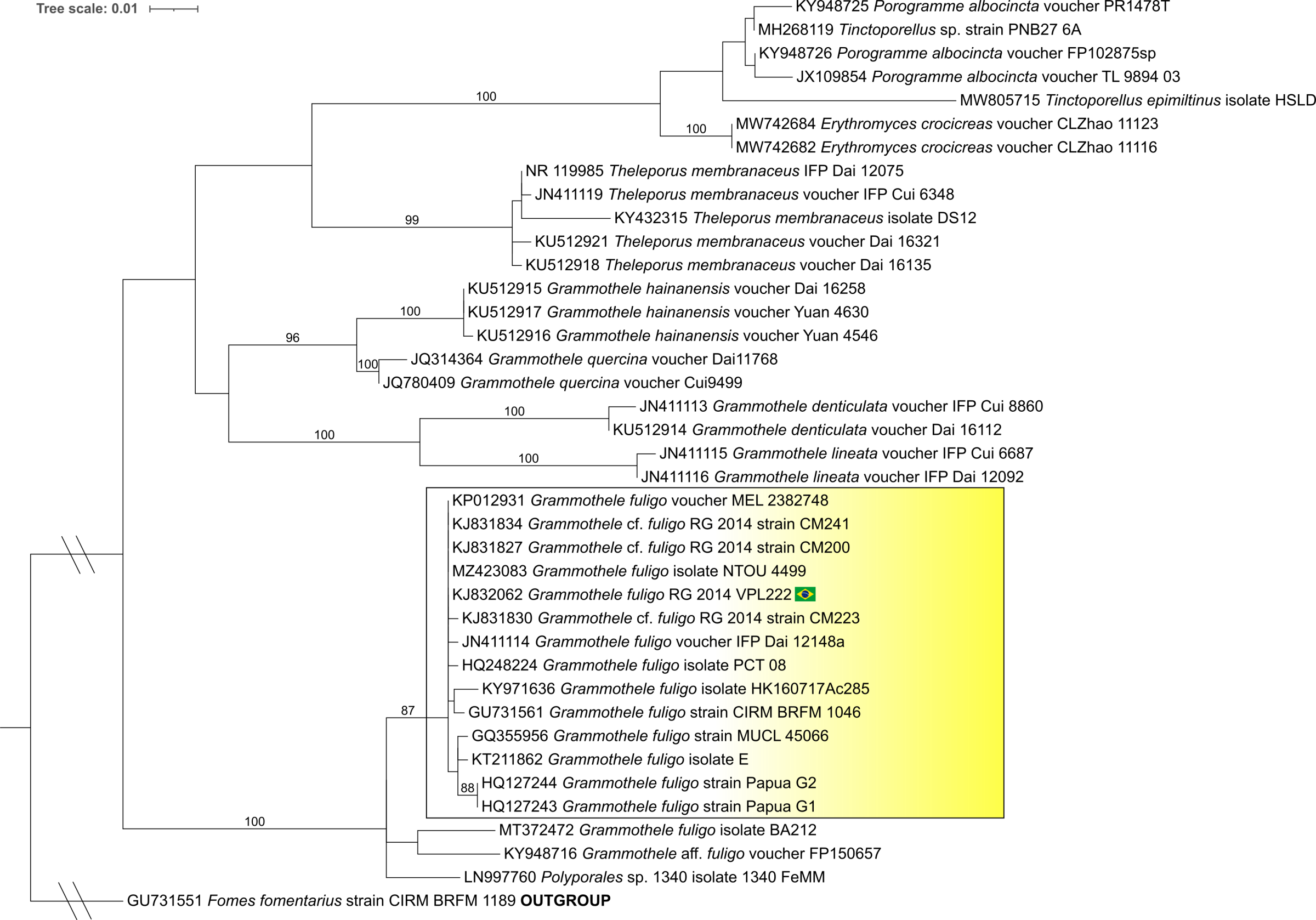


Figure S18. Maximum Likelihood (ML) tree of *Grammothele* based on ITS data. Branches are labeled with ML bootstrap higher than 80%. The highlight in yellow represents the clade of species *Grammothele fuligo*.


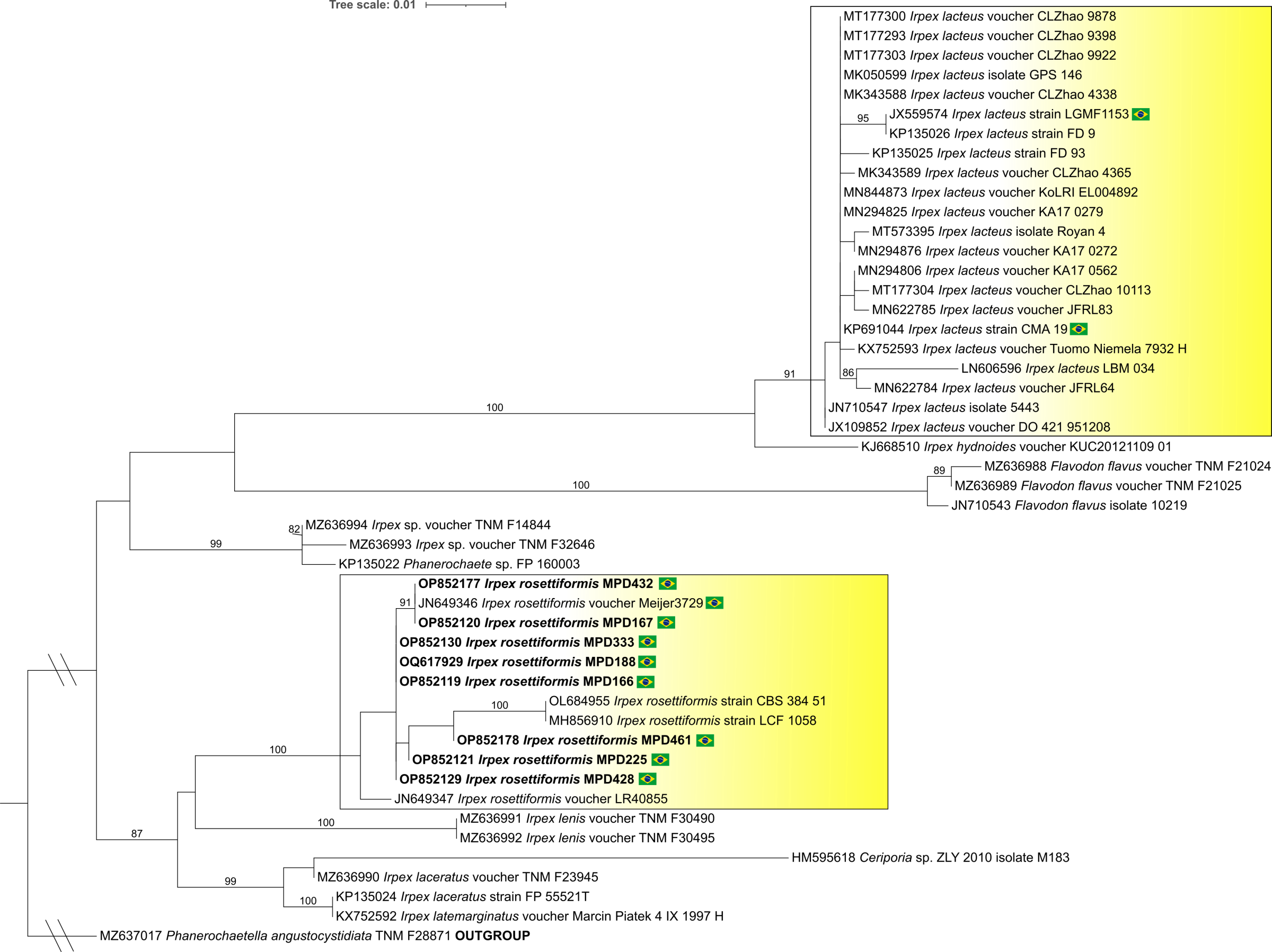


Figure S19. Maximum Likelihood (ML) tree of *Irpex* based on ITS data. Branches are labeled with ML bootstrap higher than 80%. The highlight in yellow represents the clade of species *Irpex lacteus* and *Irpex rosettiformis*. The sequences in bold were generated in this work.


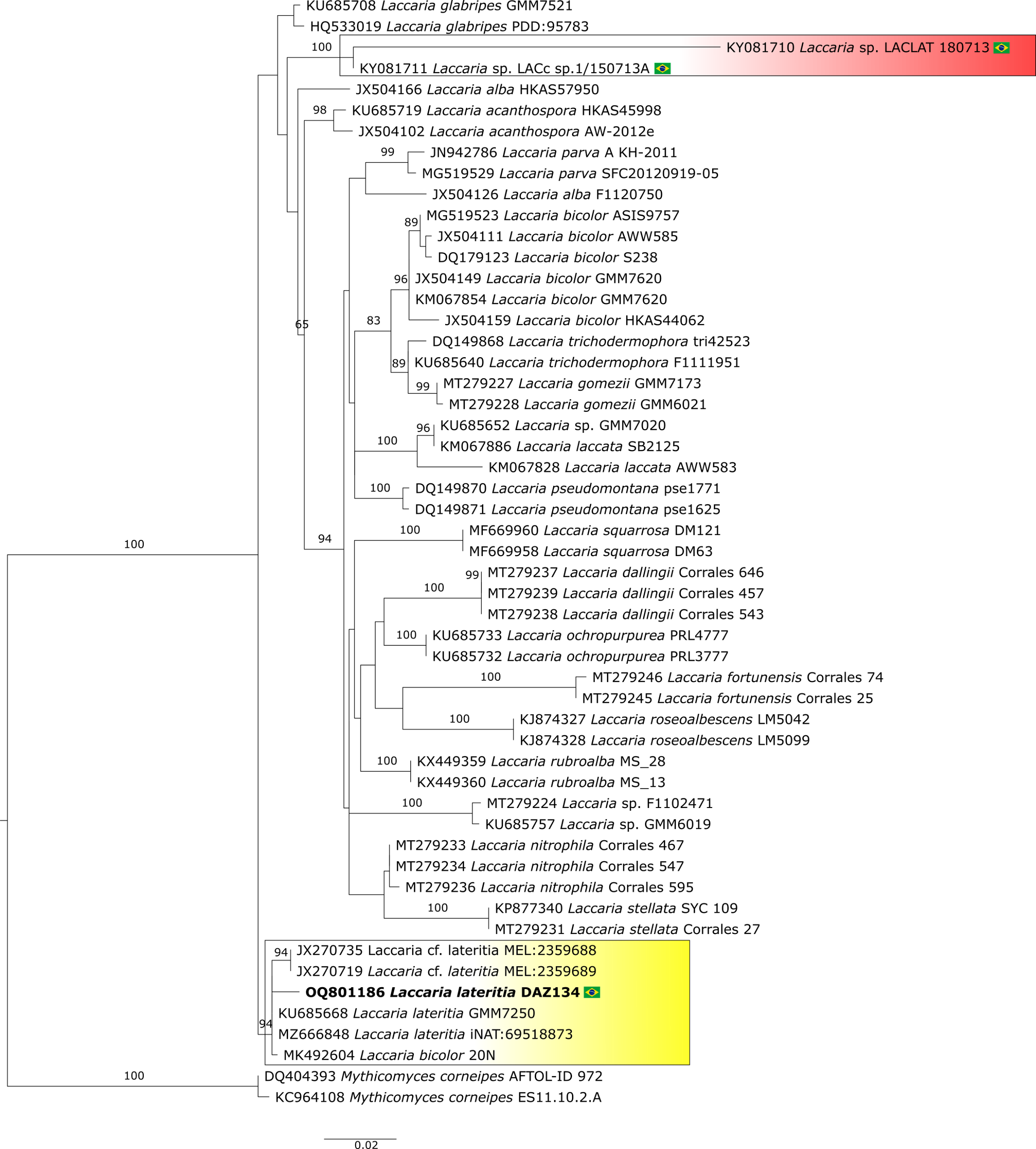


Figure S20. Maximum Likelihood (ML) tree of *Laccaria* based on ITS data. Branches are labeled with ML bootstrap higher than 80%. The highlight in yellow represents the clade of species *Laccaria lateritia*. The red highlight represents the clade with misidentified sequences. The sequence in bold was generated in this work.


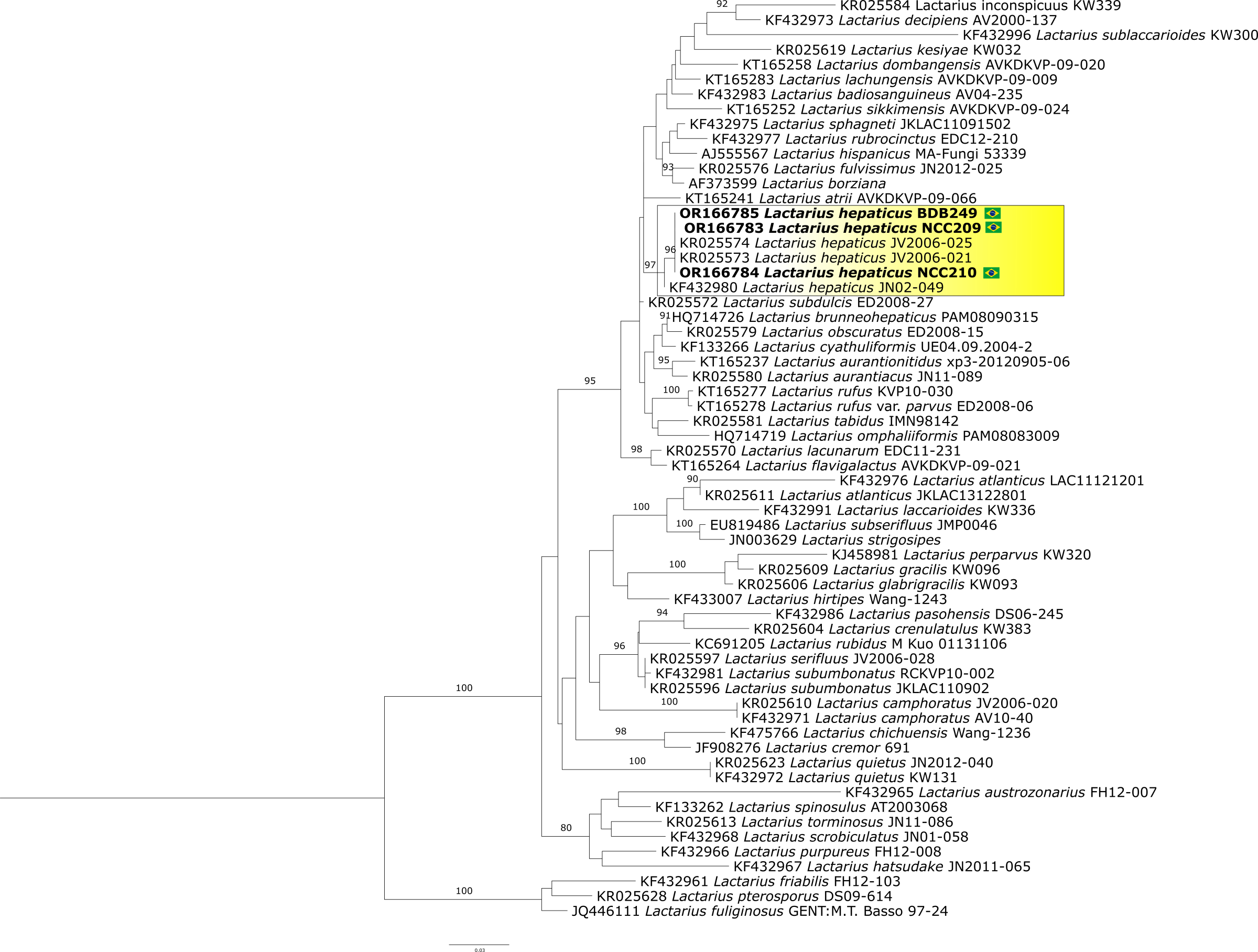


Figure S21. Maximum Likelihood (ML) tree of *Lactarius* based on ITS data. Branches are labeled with ML bootstrap higher than 80%. The highlight in yellow represents the clade of species *Lactarius hepaticus*. The sequences in bold were generated in this work.


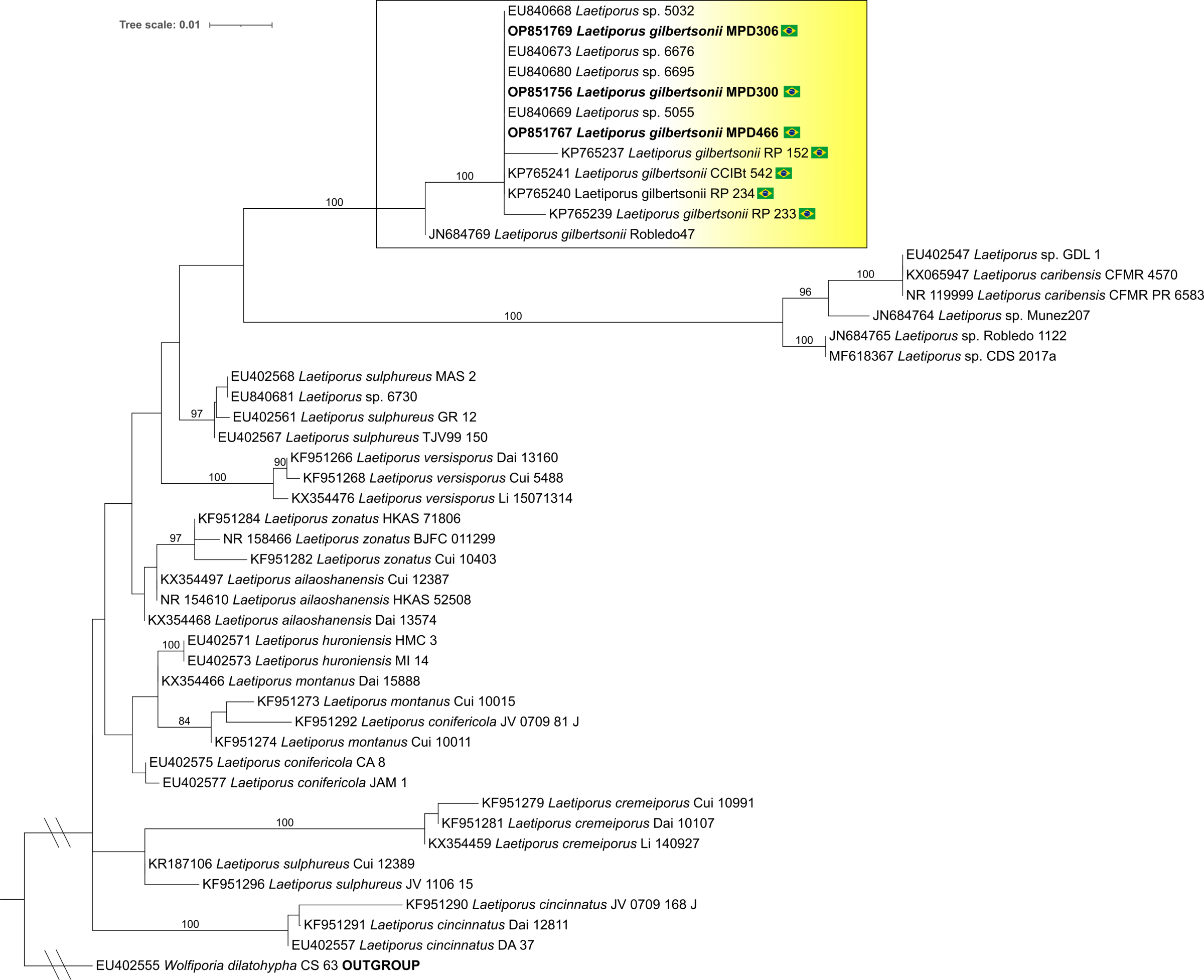


Figure S22. Maximum Likelihood (ML) tree of *Laetiporus* based on ITS data. Branches are labeled with ML bootstrap higher than 80%. The highlight in yellow represents the clade of species *Laetiporus gilbertsonii*. The sequences in bold were generated in this work.


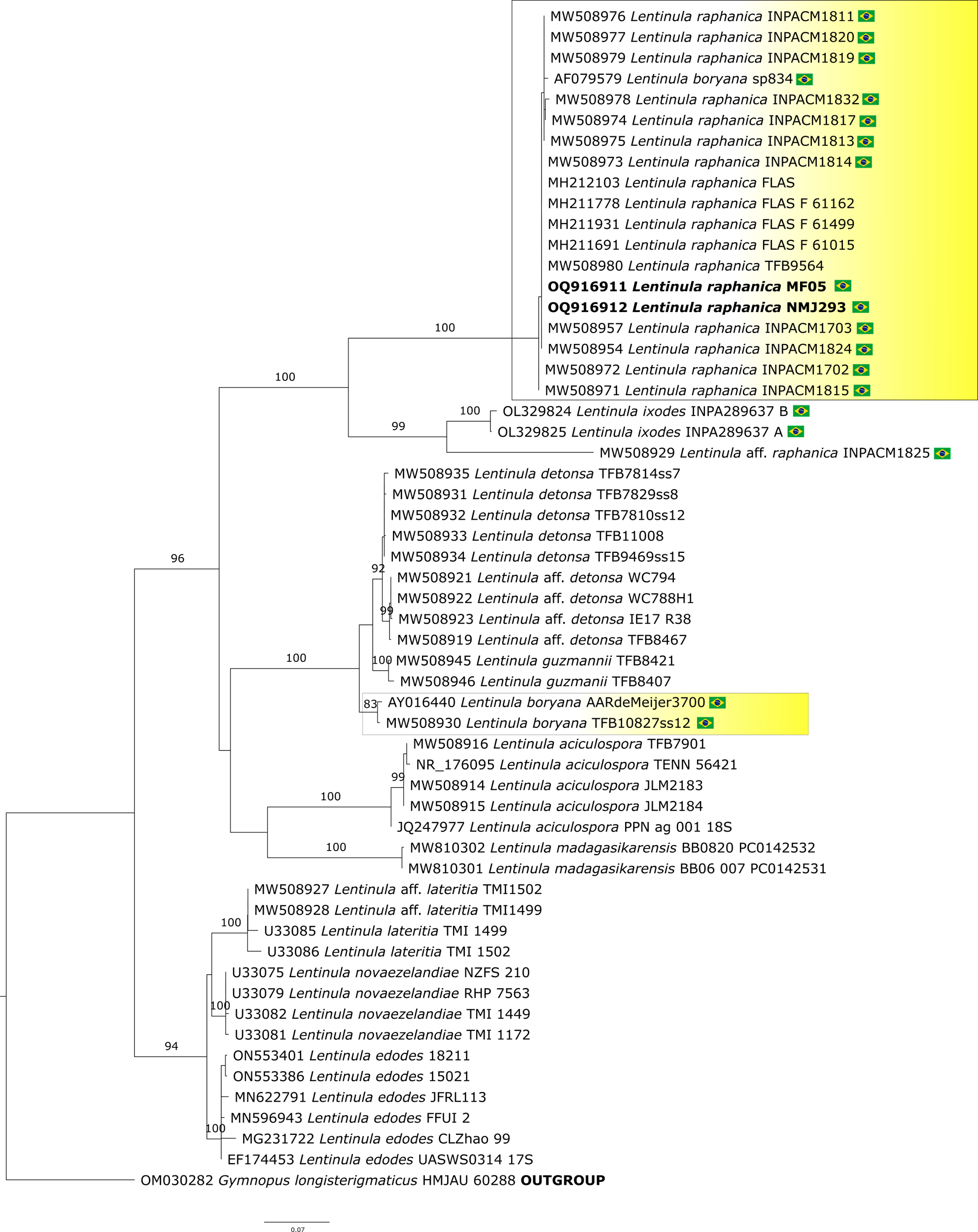


Figure S23. Maximum Likelihood (ML) tree of *Lentinula* based on ITS data. Branches are labeled with ML bootstrap higher than 80%. The highlight in yellow represents the clade of species *Lentinula boryana* and *Lentinula raphanica*. The sequences in bold were generated in this work.


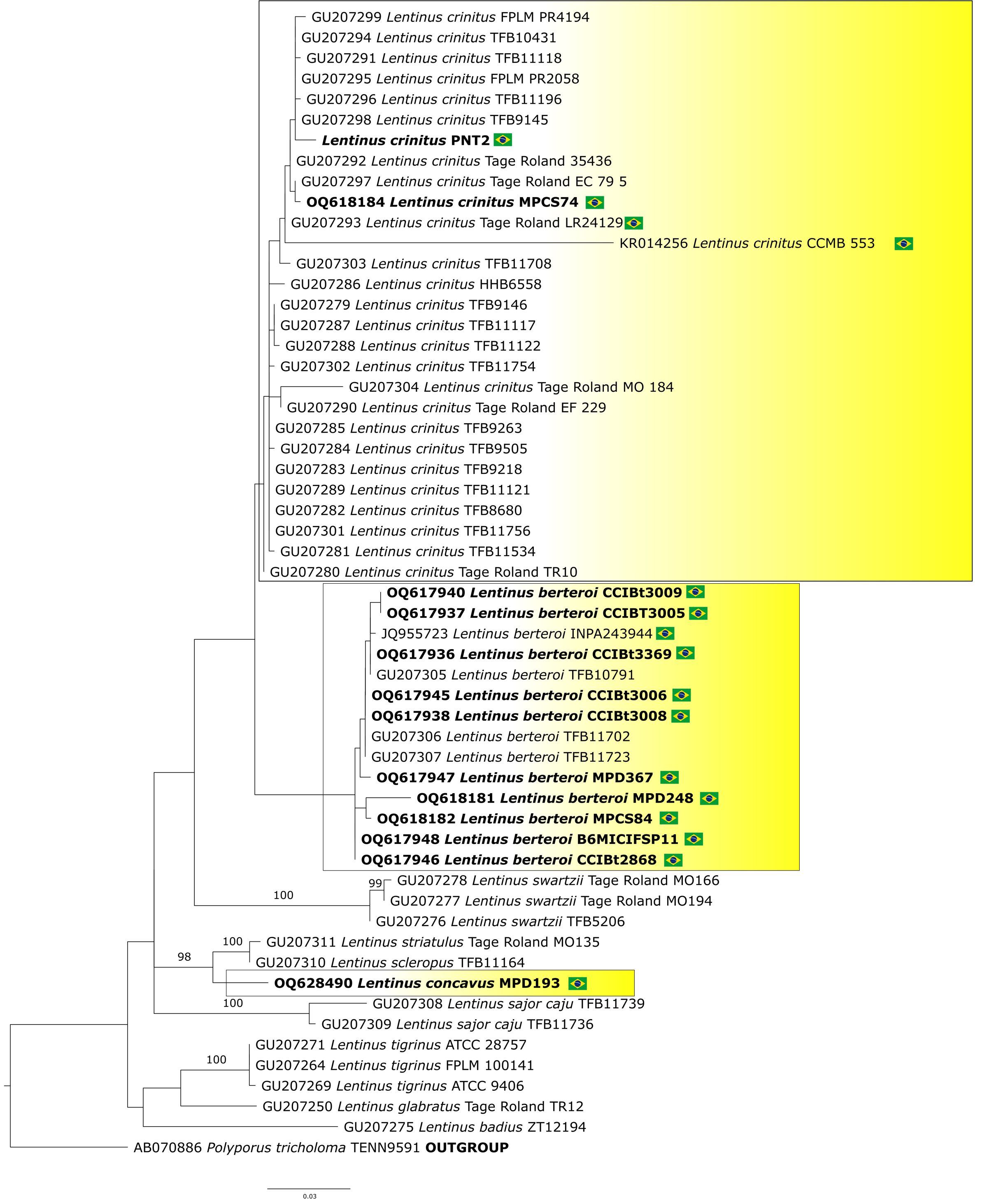


Figure S24. Maximum Likelihood (ML) tree of *Lentinus* based on ITS data. Branches are labeled with ML bootstrap higher than 80%. The highlight in yellow represents the clade of species *Lentinus berteroi*, *Lentinus concavus*, and *Lentinus crinitus*. The sequences in bold were generated in this work.


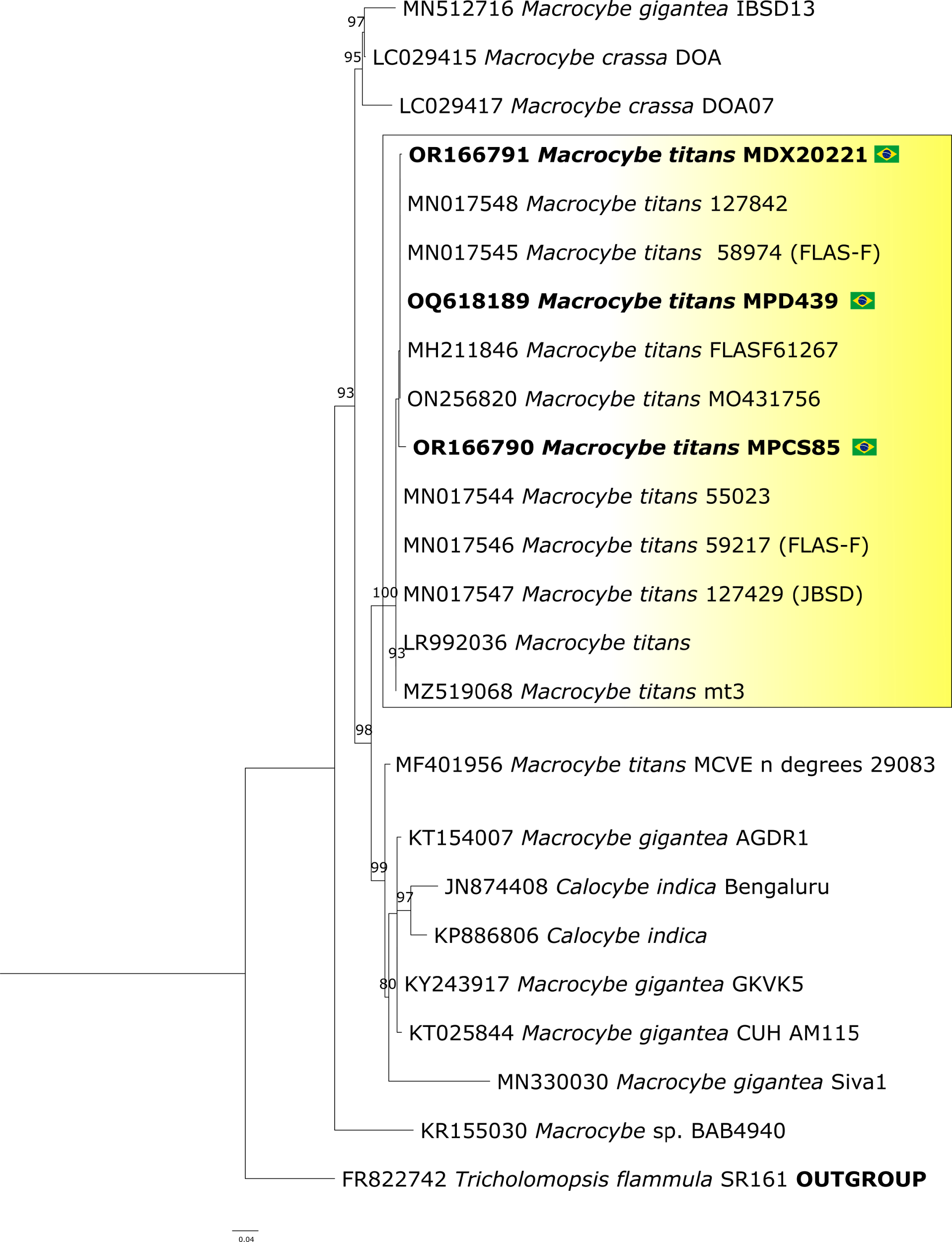


Figure S25. Maximum Likelihood (ML) tree of *Macrocybe* based on ITS data. Branches are labeled with ML bootstrap higher than 80%. The highlight in yellow represents the clade of species *Macrocybe titans*. The sequences in bold were generated in this work.


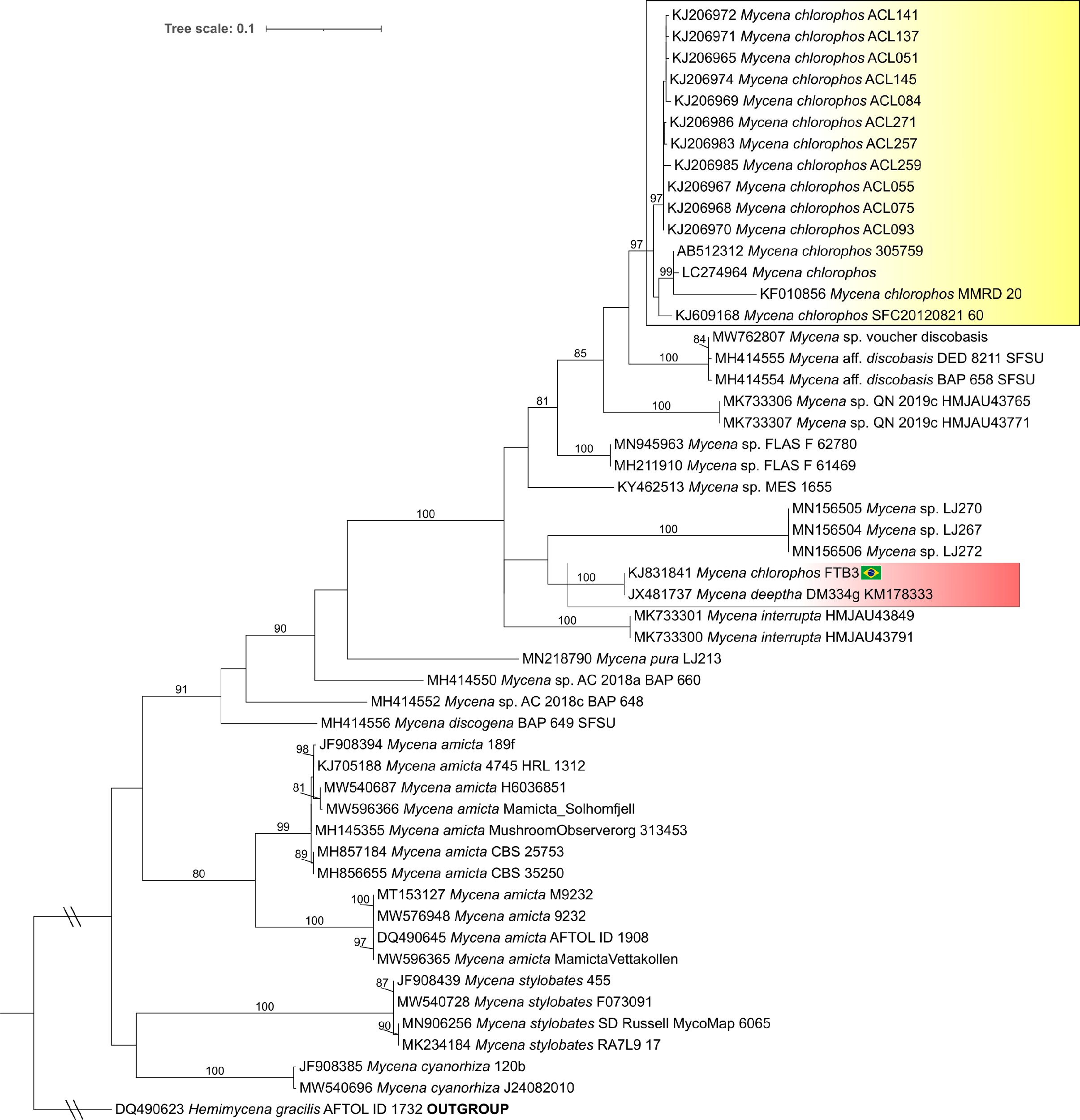


Figure S26. Maximum Likelihood (ML) tree of *Mycena* based on ITS data. Branches are labeled with ML bootstrap higher than 80%. The highlight in yellow represents the clade of species *Mycena chlorophos*. The red highlight represents the clade with the misidentified sequence.


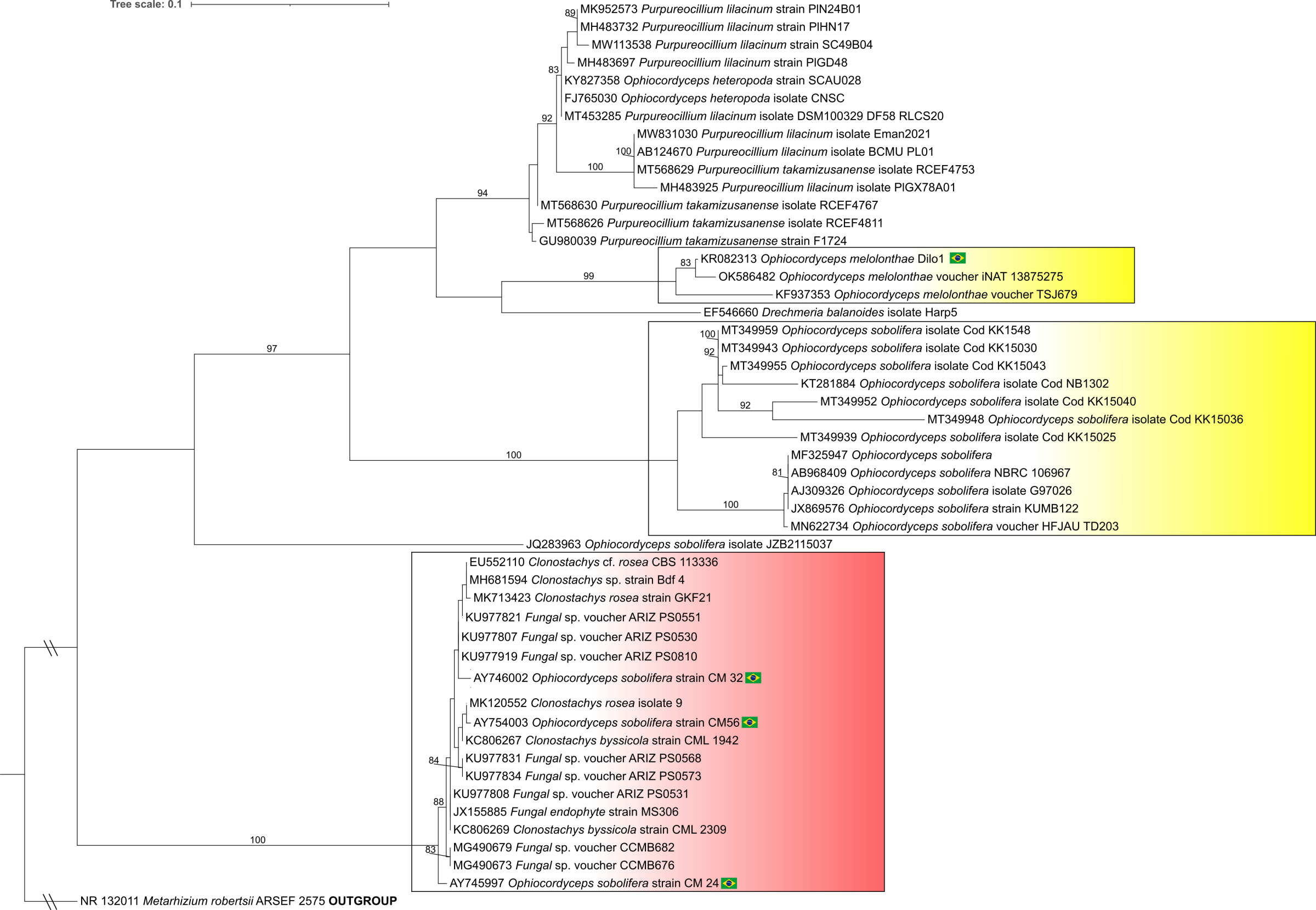


Figure S27. Maximum Likelihood (ML) tree of *Ophiocordyceps* based on ITS data. Branches are labeled with ML bootstrap higher than 80%. The highlight in yellow represents the clade of species *Ophiocordyceps melolonthae* and *Ophiocordyceps sobolifera*. The red highlight represents the clade with the misidentified sequences.


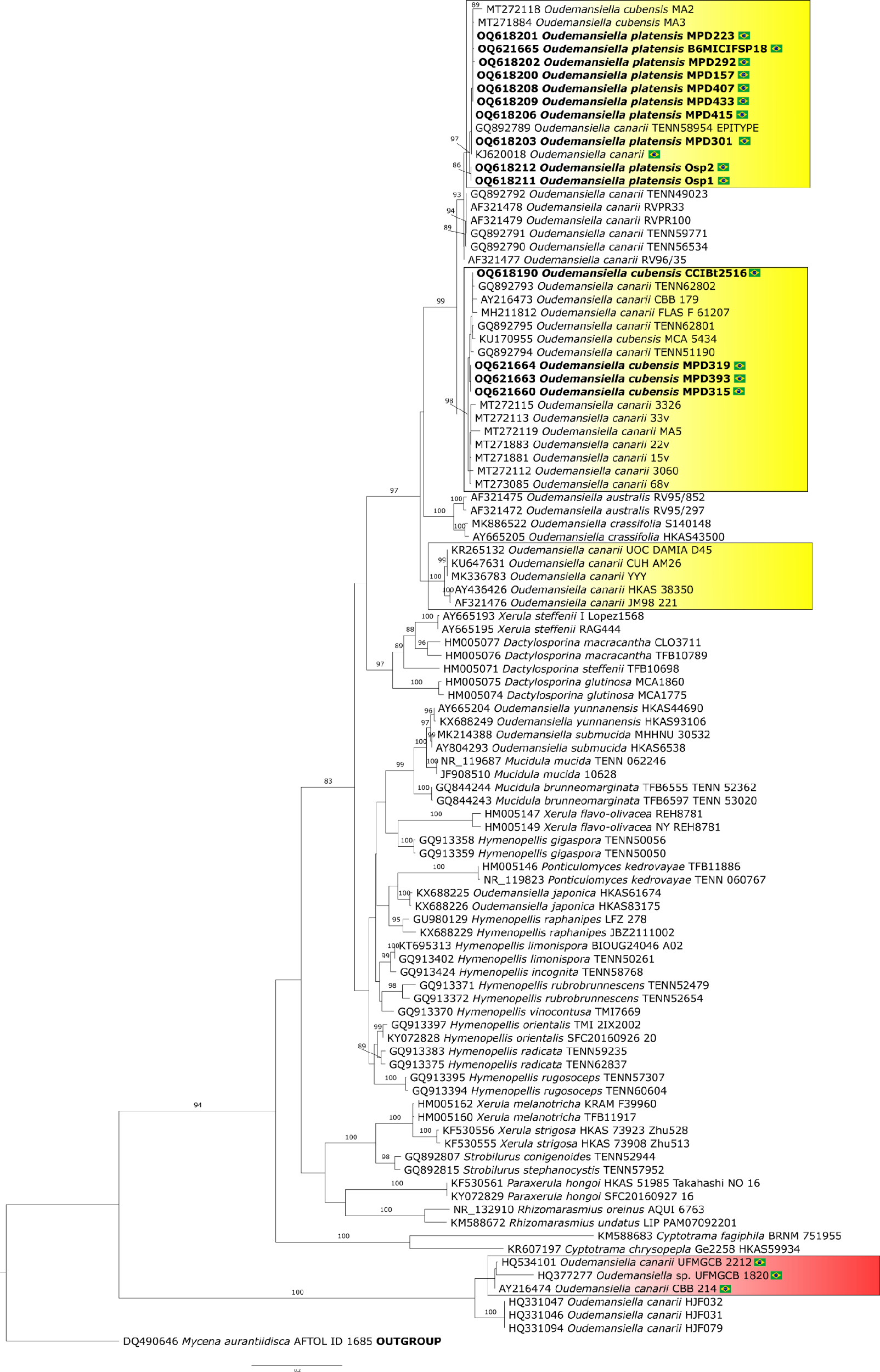


Figure S28. Maximum Likelihood (ML) tree of *Oudemansiella* and allied genera based on ITS data. Branches are labeled with ML bootstrap higher than 80%. The highlight in yellow represents the clade of species *Oudemansiella canarii*, *Oudemansiella cubensis*, and *Oudemansiella platensis*. The red highlight represents the clade with the misidentified sequences. The sequences in bold were generated in this work.


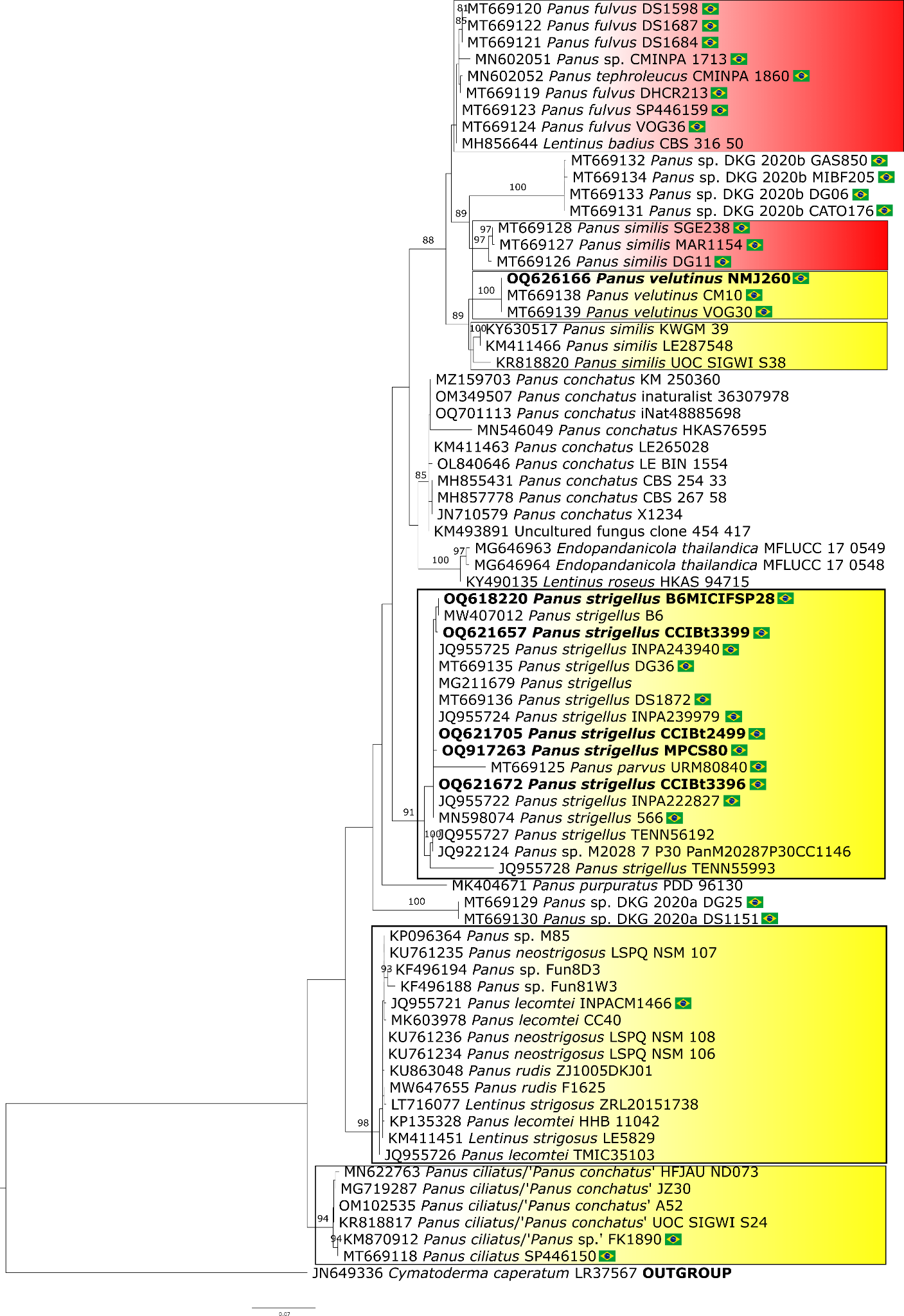


Figure S29. Maximum Likelihood (ML) tree of *Panus* based on ITS data. Branches are labeled with ML bootstrap higher than 80%. The highlight in yellow represents the clade of species *Panus ciliatus*, *Panus neostrigosus*, *Panus similis*, *Panus strigellus*, and *Panus velutinus*. The red highlight represents the clades with the unconfirmed sequences. The sequences in bold were generated in this work.


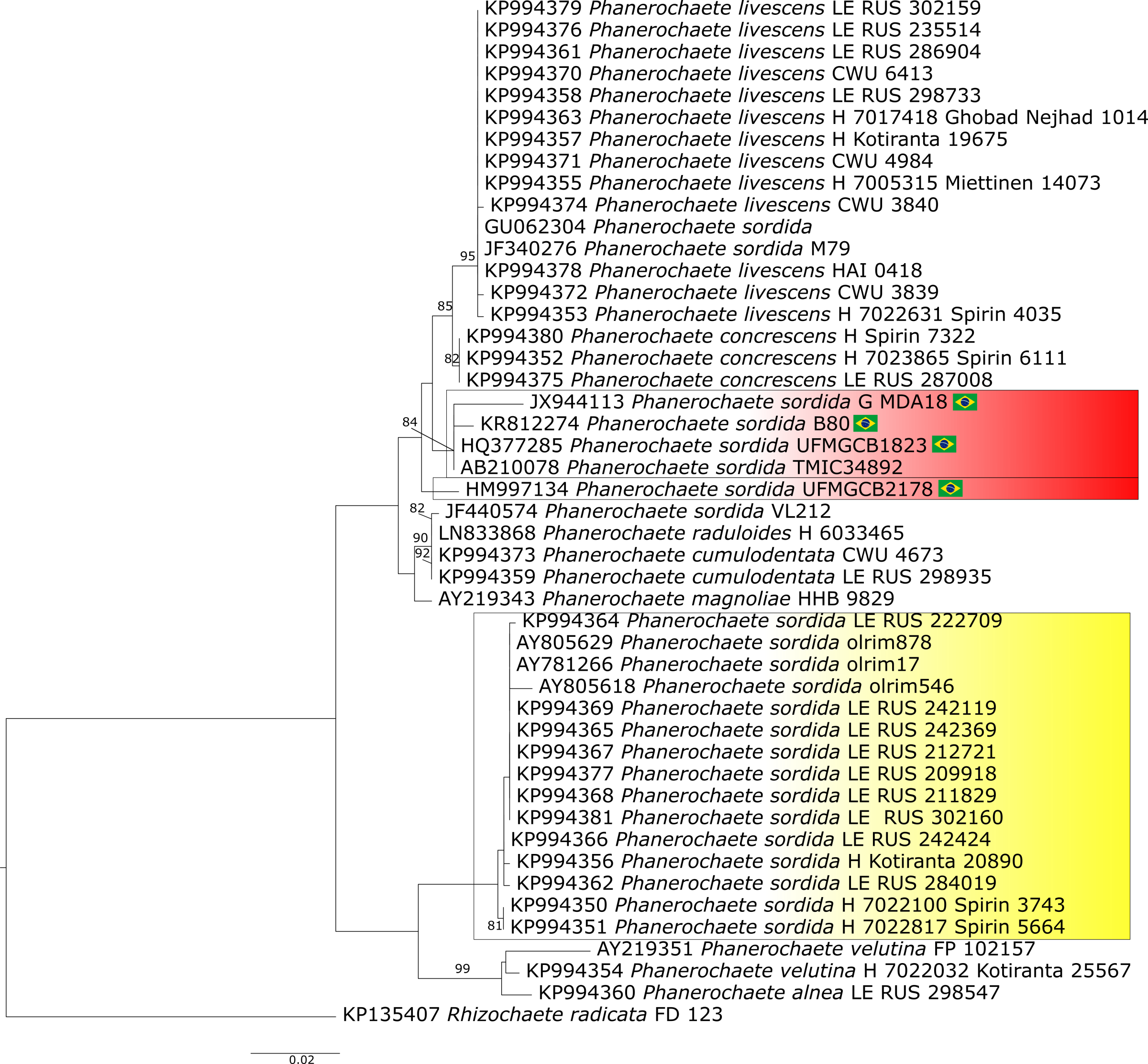


Figure S30. Maximum Likelihood (ML) tree of *Phanerochaete* based on ITS data. Branches are labeled with ML bootstrap higher than 80%. The highlight in yellow represents the clade of species *Phanerochaete sordida*. The red highlight represents the clades with the misidentified sequences.


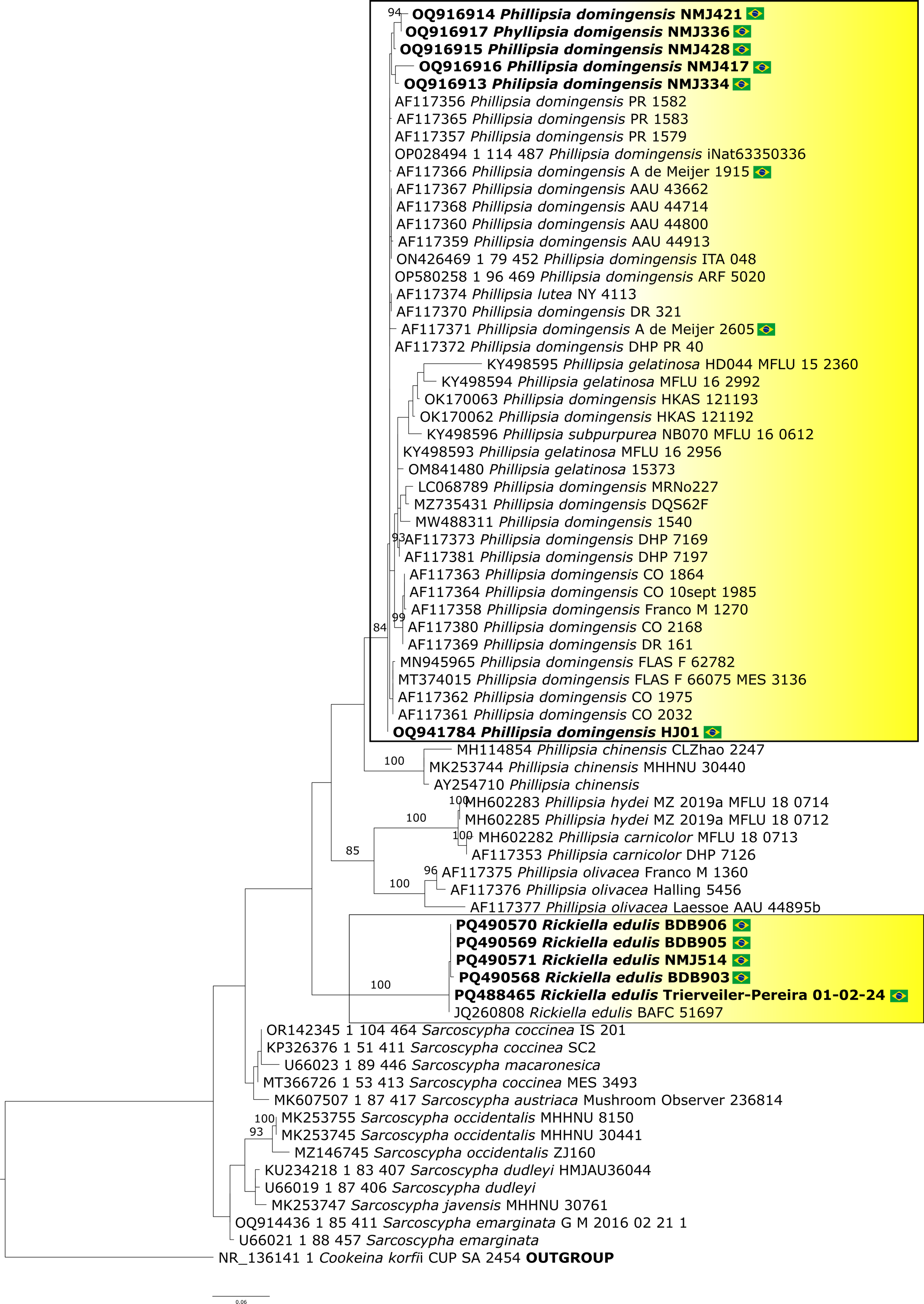


Figure S31. Maximum Likelihood (ML) tree of *Phillipsia* and *Rickiella* based on ITS data. Branches are labeled with ML bootstrap higher than 80%. The highlight in yellow represents the clade of species *Phillipsia dominguensis* and *Rickiella* edulis. The sequences in bold were generated in this work.


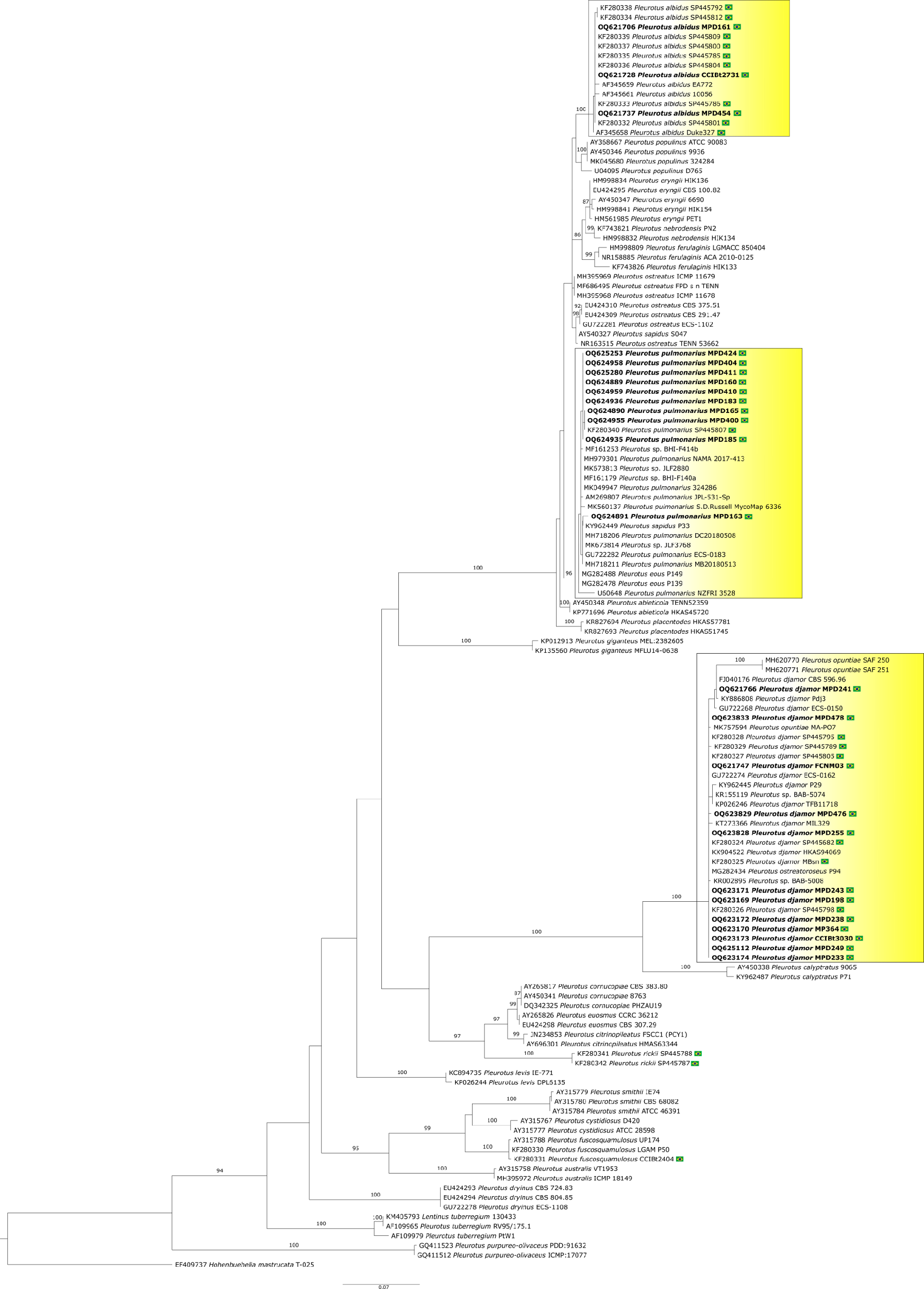


Figure S32. Maximum Likelihood (ML) tree of *Pleurotus* based on ITS data. Branches are labeled with ML bootstrap higher than 80%. The highlight in yellow represents the clade of species *Pleurotus albidus*, *Pleurotus djamor,* and *Pleurotus pulmonarius*. The sequences in bold were generated in this work.


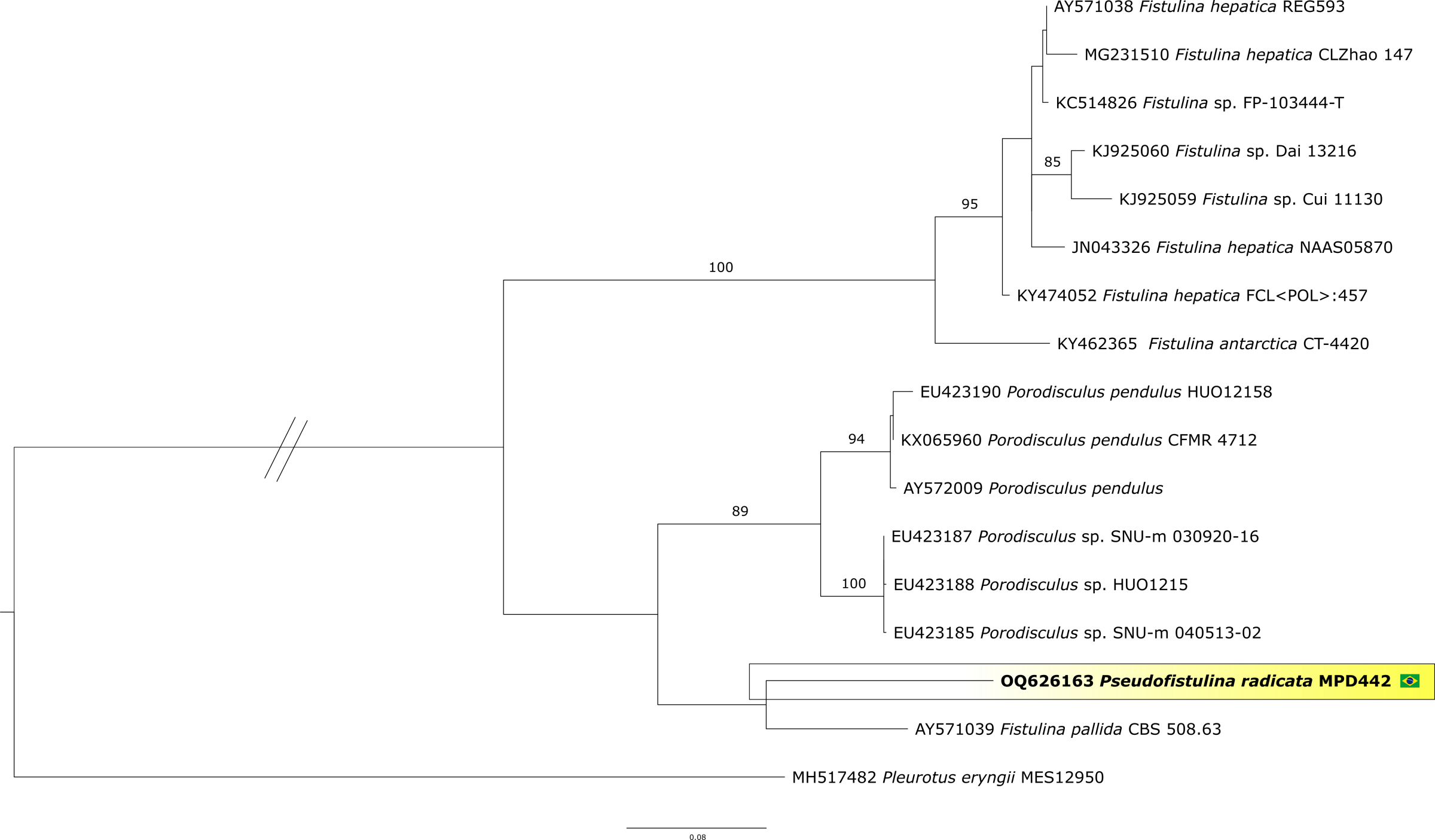


Figure S33. Maximum Likelihood (ML) tree of *Pseudofistulina* and allied genera based on ITS data. Branches are labeled with ML bootstrap higher than 80%. The highlight in yellow represents the clade of species *Pseudofistulina radicata*. The sequence in bold was generated in this work.


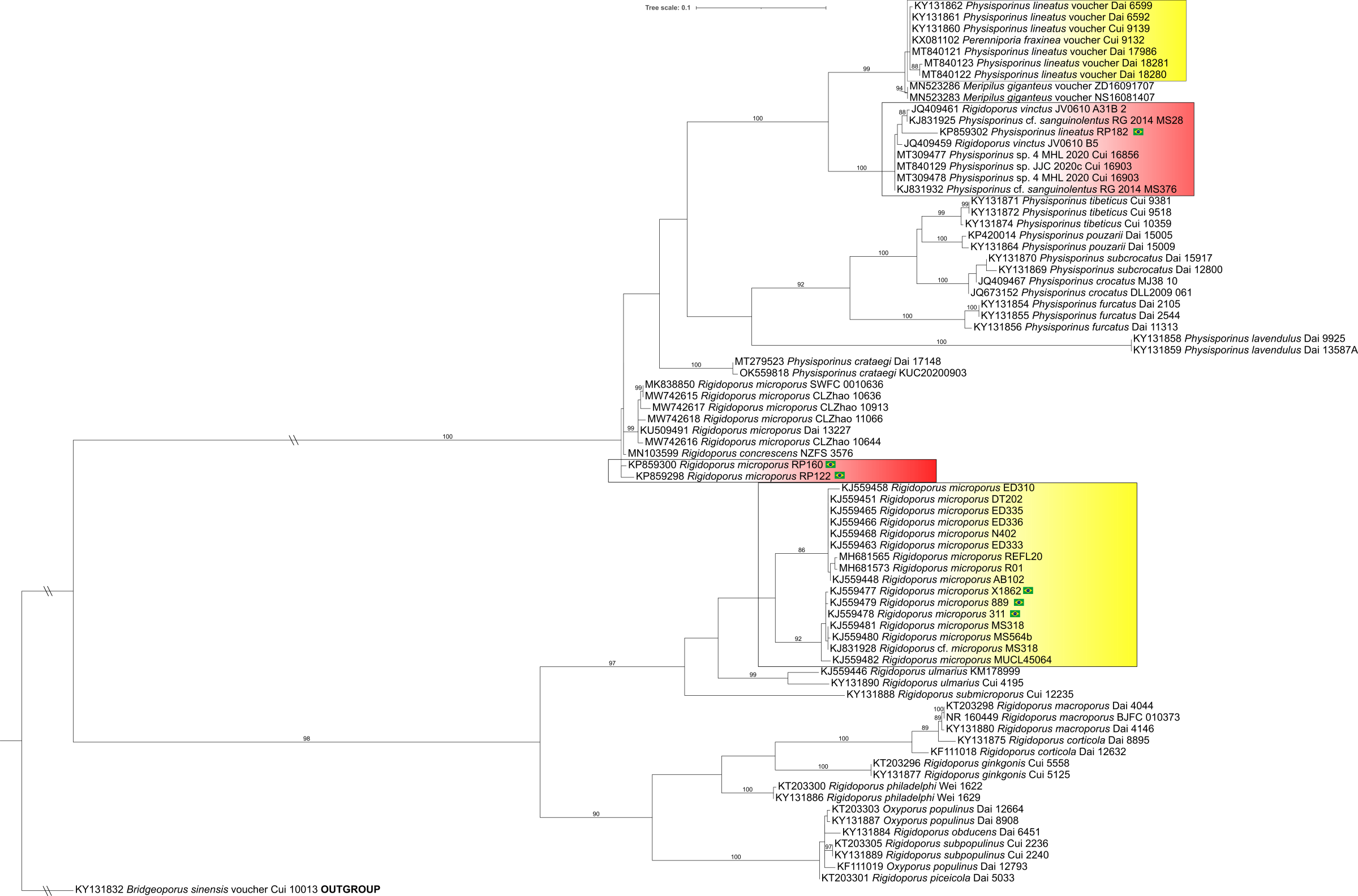


Figure S34. Maximum Likelihood (ML) tree of *Rigidoporus* and allied based on ITS data. Branches are labeled with ML bootstrap higher than 80%. The highlight in yellow represents the clade of species *Rigidoporus microporus* and *Physisporinus lineatus*. The red highlight represents the clades with misidentified sequences.


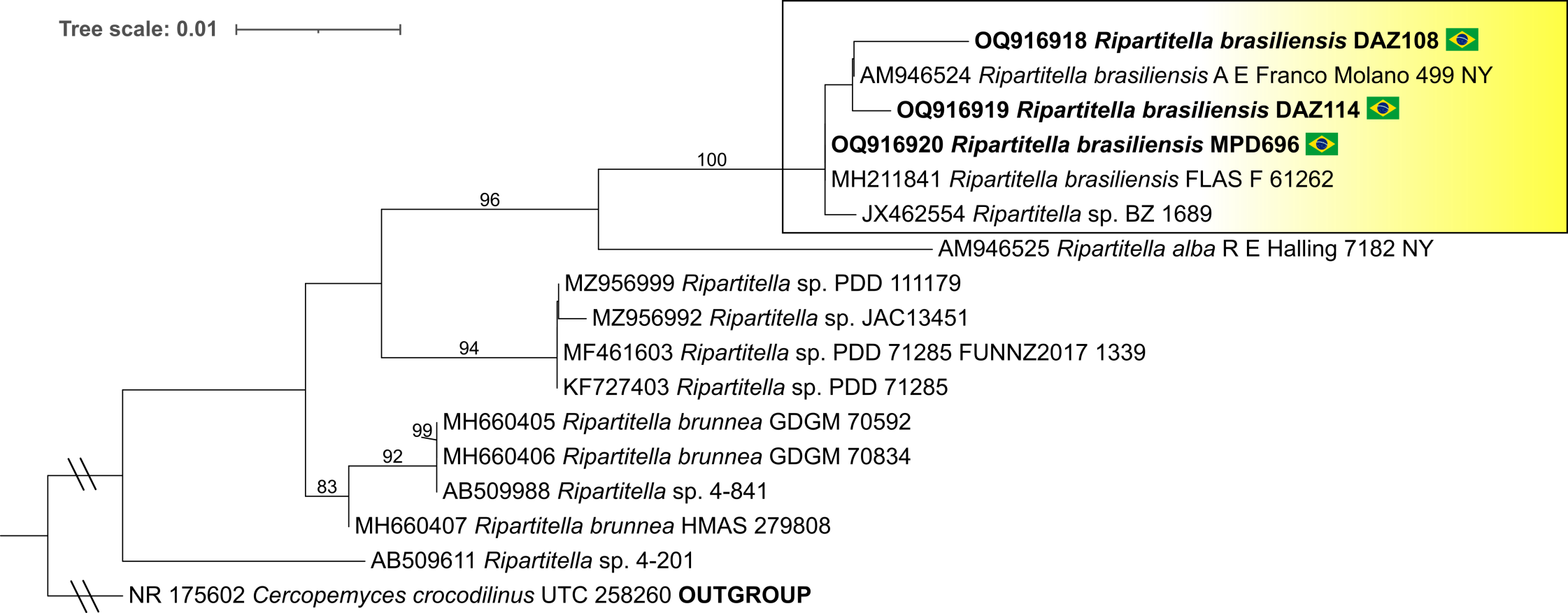


Figure S35. Maximum Likelihood (ML) tree of *Ripartitella* based on ITS data. Branches are labeled with ML bootstrap higher than 80%. The highlight in yellow represents the clade of species *Ripartitella brasiliensis*. The sequences in bold were generated in this study.


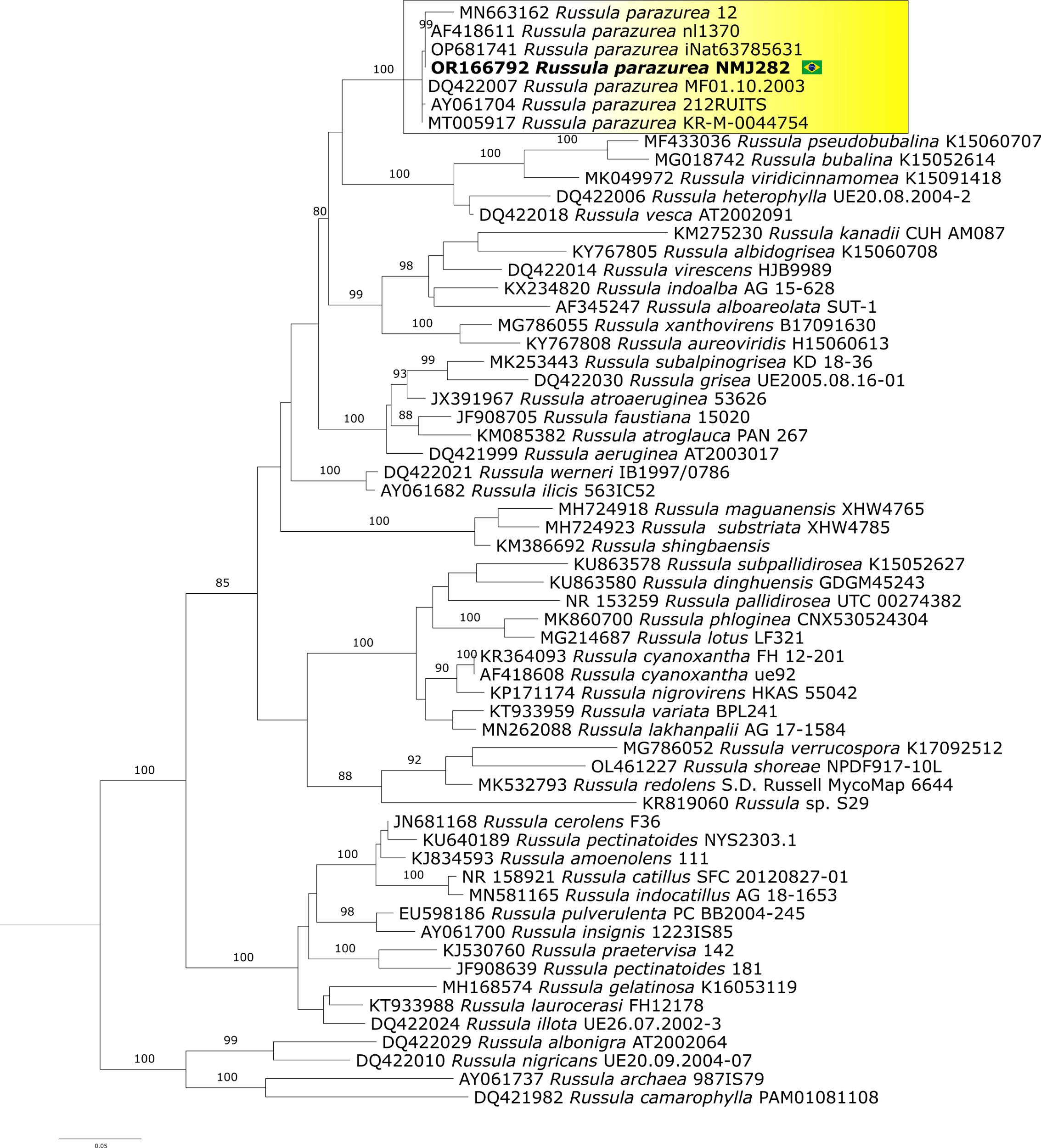


Figure S36. Maximum Likelihood (ML) tree of *Russula* based on ITS data. Branches are labeled with ML bootstrap higher than 80%. The highlight in yellow represents the clade of species *Russula parazurea*. The sequence in bold was generated in this study.


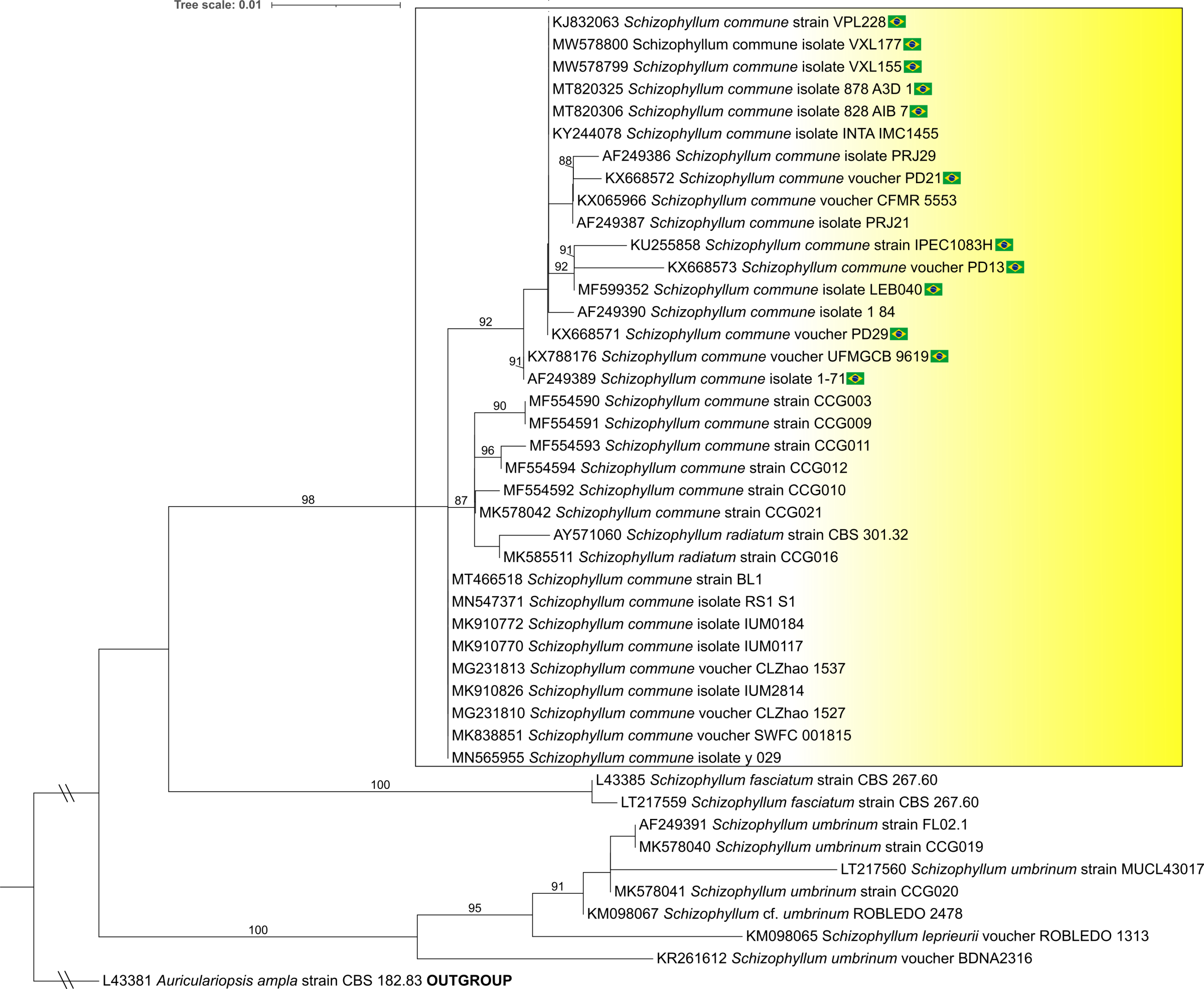


Figure S37. Maximum Likelihood (ML) tree of *Schizophyllum* based on ITS data. Branches are labeled with ML bootstrap higher than 80%. The highlight in yellow represents the clade of species *Schizophyllum commune*.


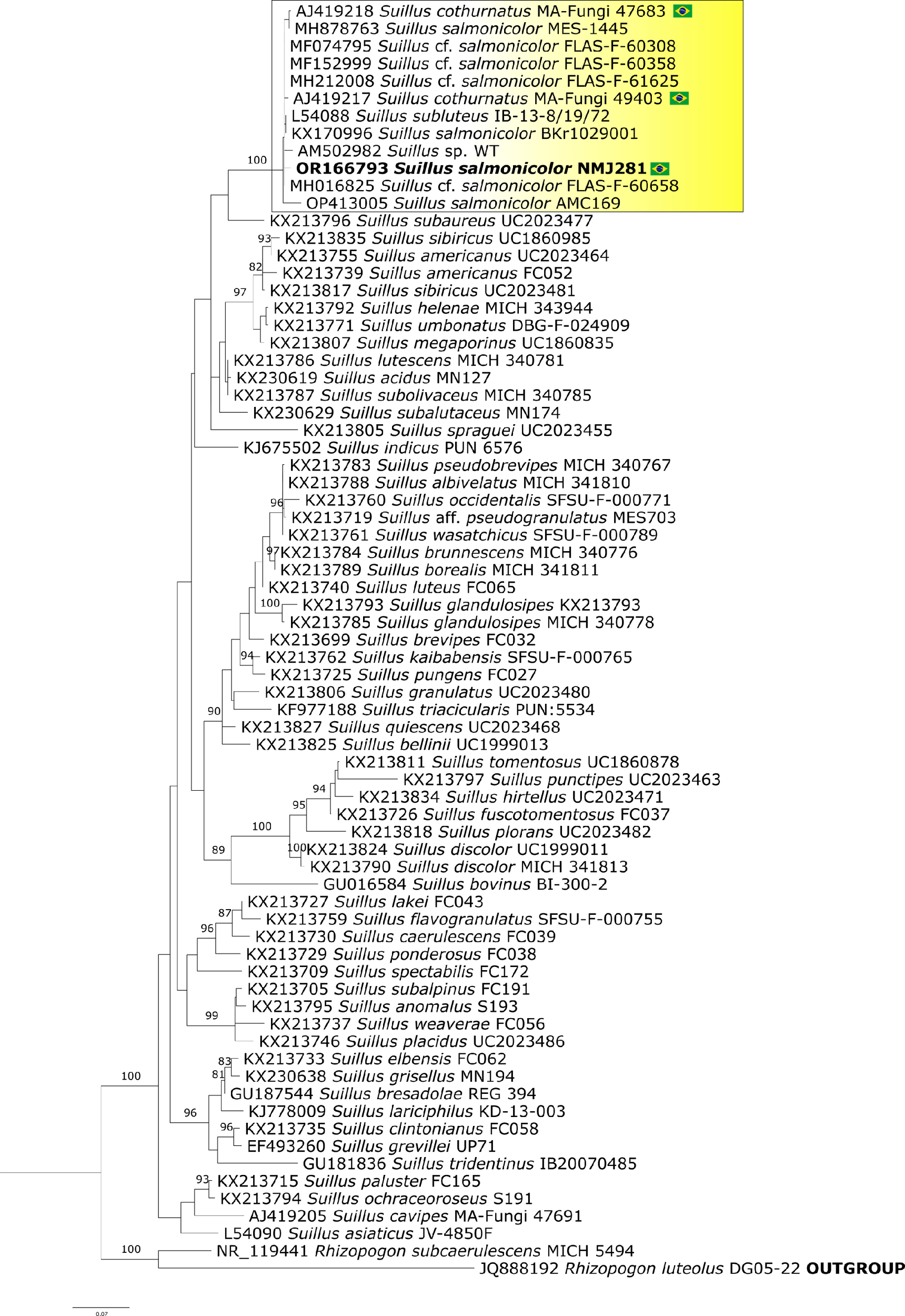


Figure S38. Maximum Likelihood (ML) tree of *Suillus* based on ITS data. Branches are labeled with ML bootstrap higher than 80%. The highlight in yellow represents the clade of species *Suillus salmonicolor*. The sequence in bold was generated in this study.


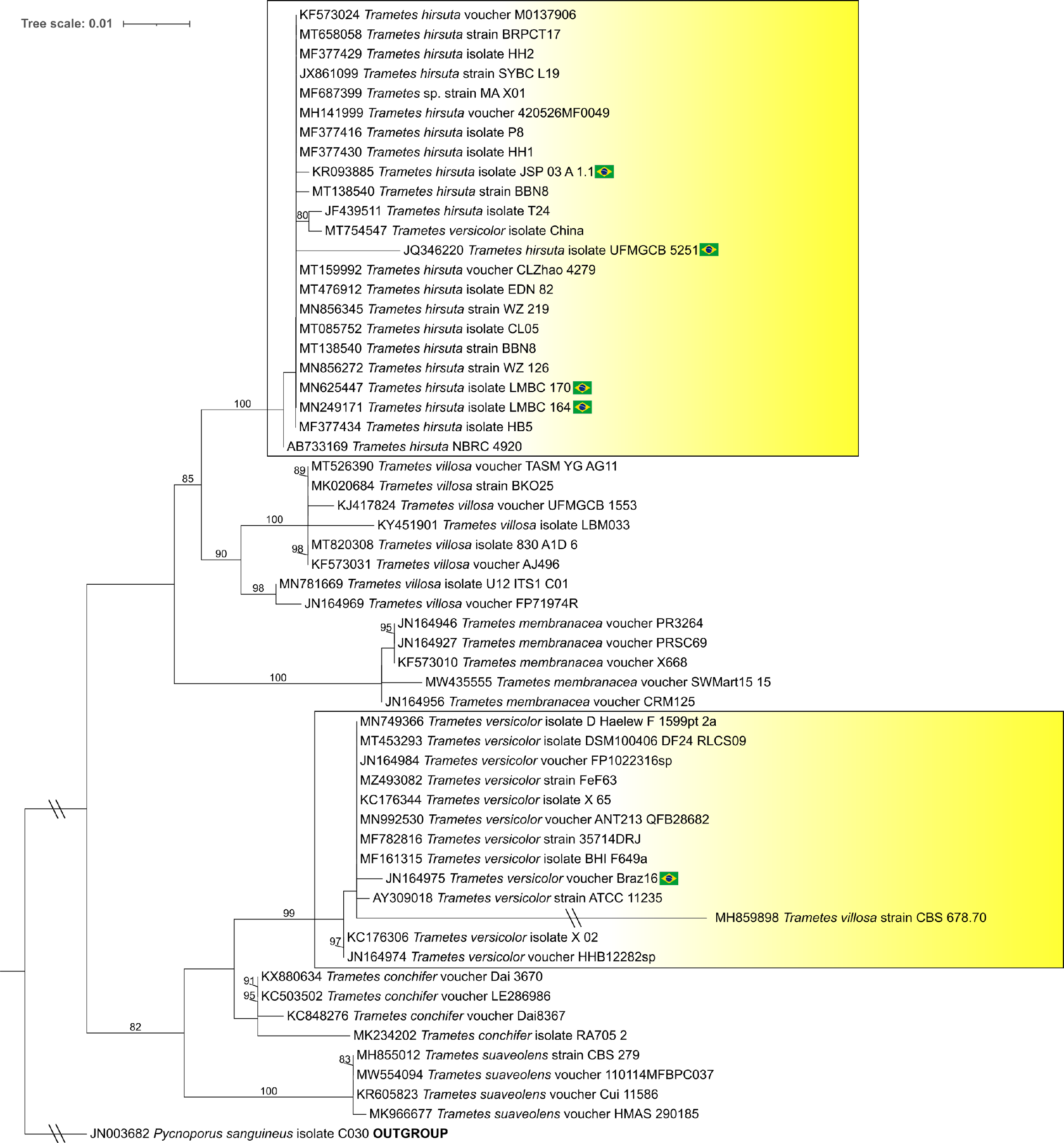


Figure S39. Maximum Likelihood (ML) tree of *Trametes* based on ITS data. Branches are labeled with ML bootstrap higher than 80%. The highlight in yellow represents the clade of species *Trametes hirsuta* and *Trametes versicolor*.


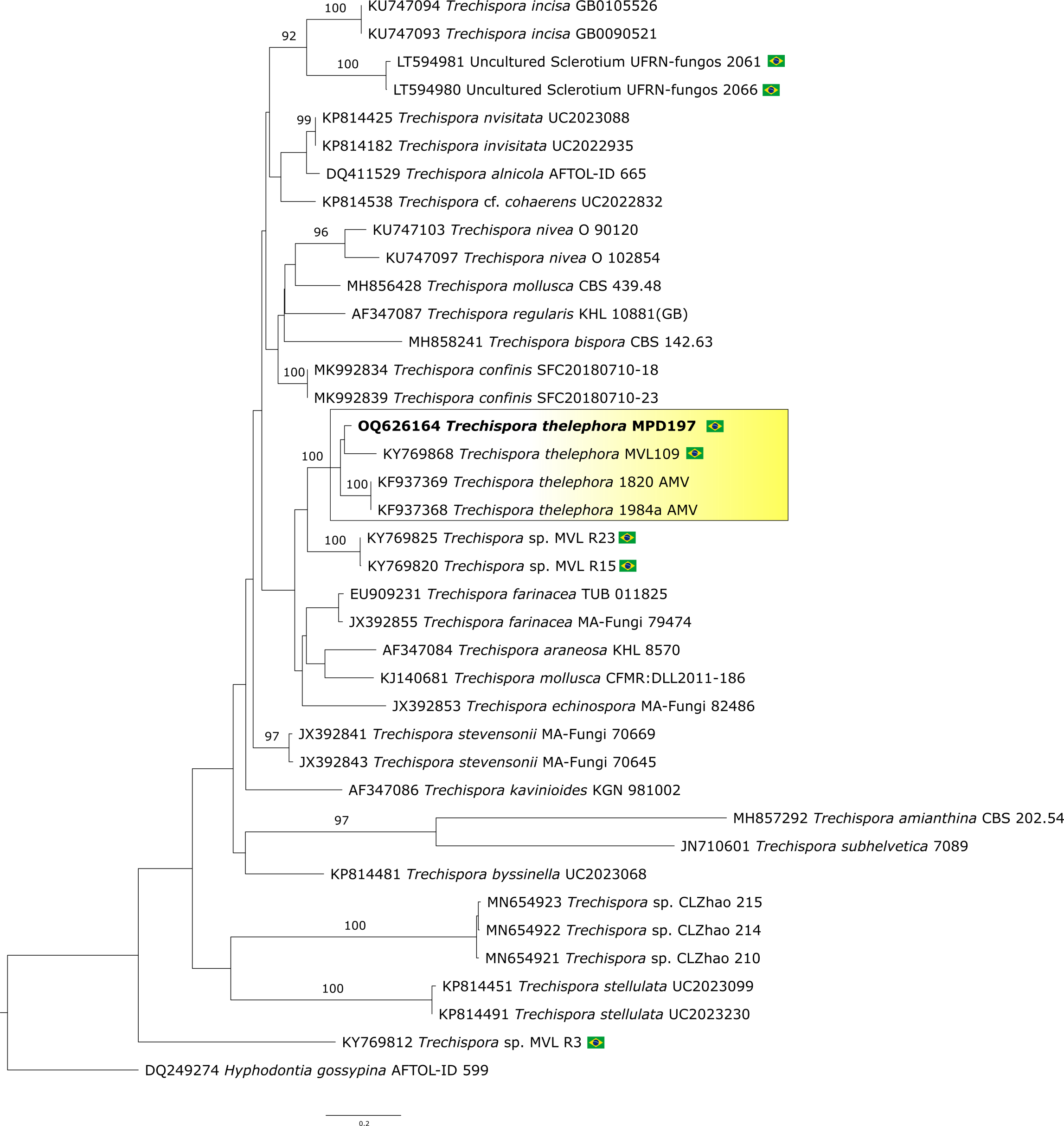


Figure S40. Maximum Likelihood (ML) tree of *Trechispora* based on ITS data. Branches are labeled with ML bootstrap higher than 80%. The highlight in yellow represents the clade of species *Trechispora thelephora*. The sequence in bold was generated in this study.


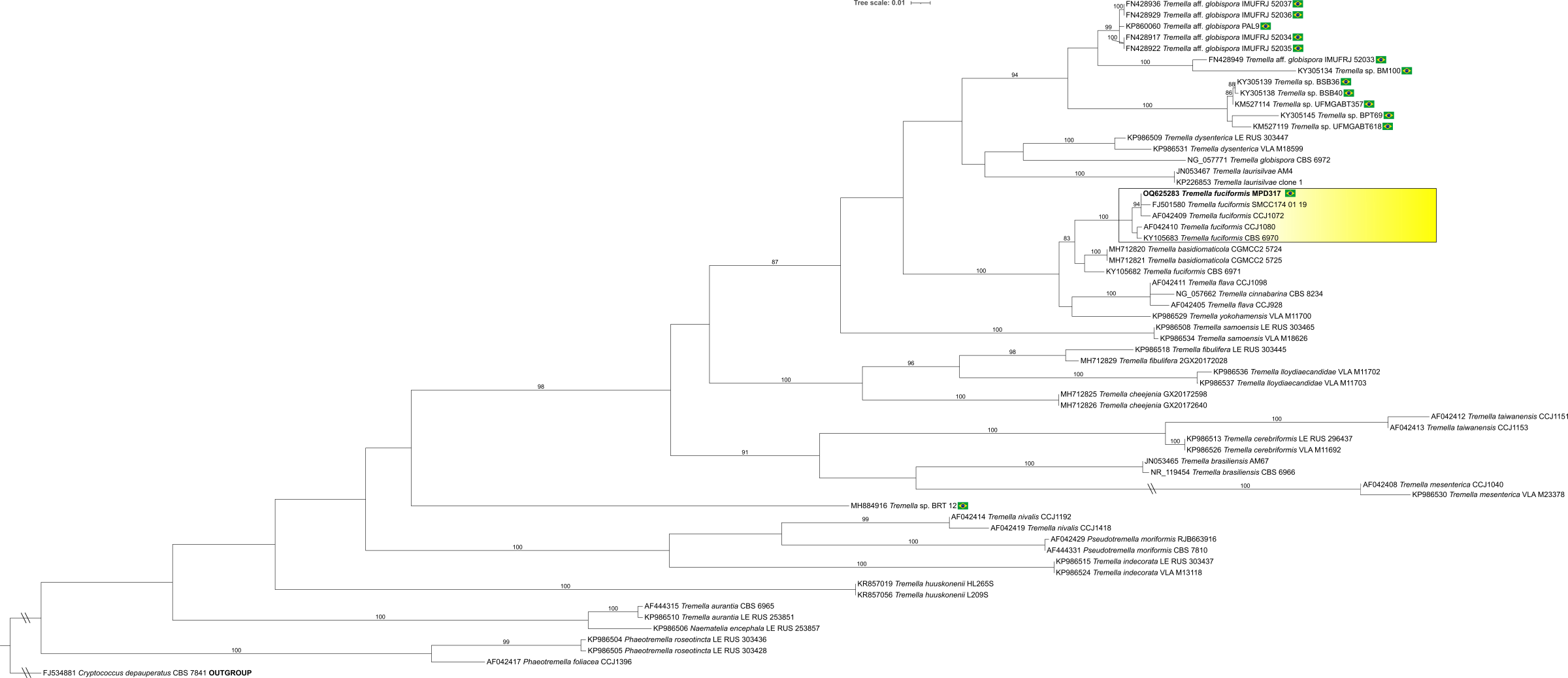


Figure S41. Maximum Likelihood (ML) tree of *Tremella* based on ITS data. Branches are labeled with ML bootstrap higher than 80%. The highlight in yellow represents the clade of species *Tremella fuciformis*. The sequence in bold was generated in this study.


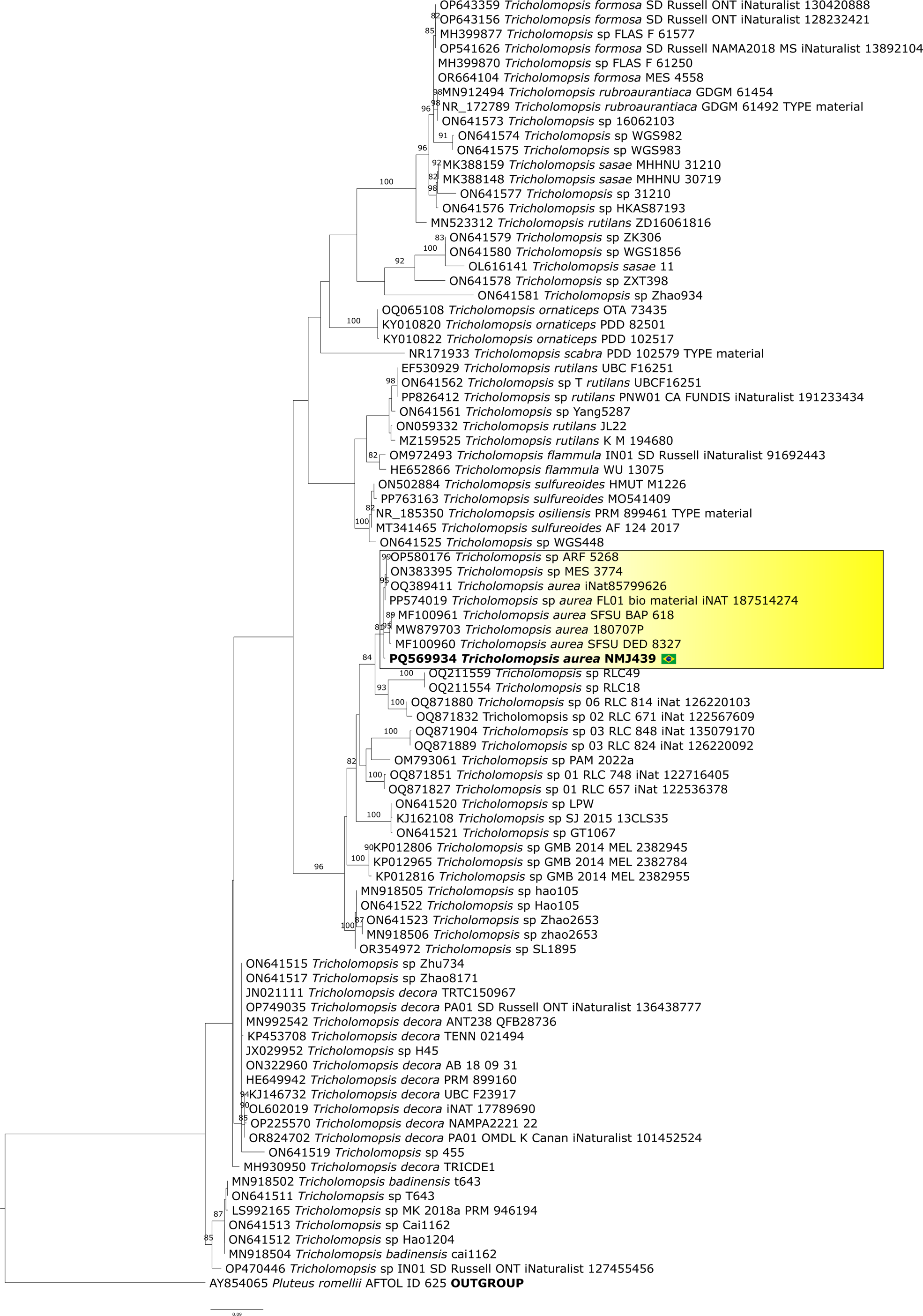


Figure S42. Maximum Likelihood (ML) tree of *Tricholomopsis* based on ITS data. Branches are labeled with ML bootstrap higher than 80%. The highlight in yellow represents the clade of species *Tricholomopsis aurea*. The sequence in bold was generated in this study.


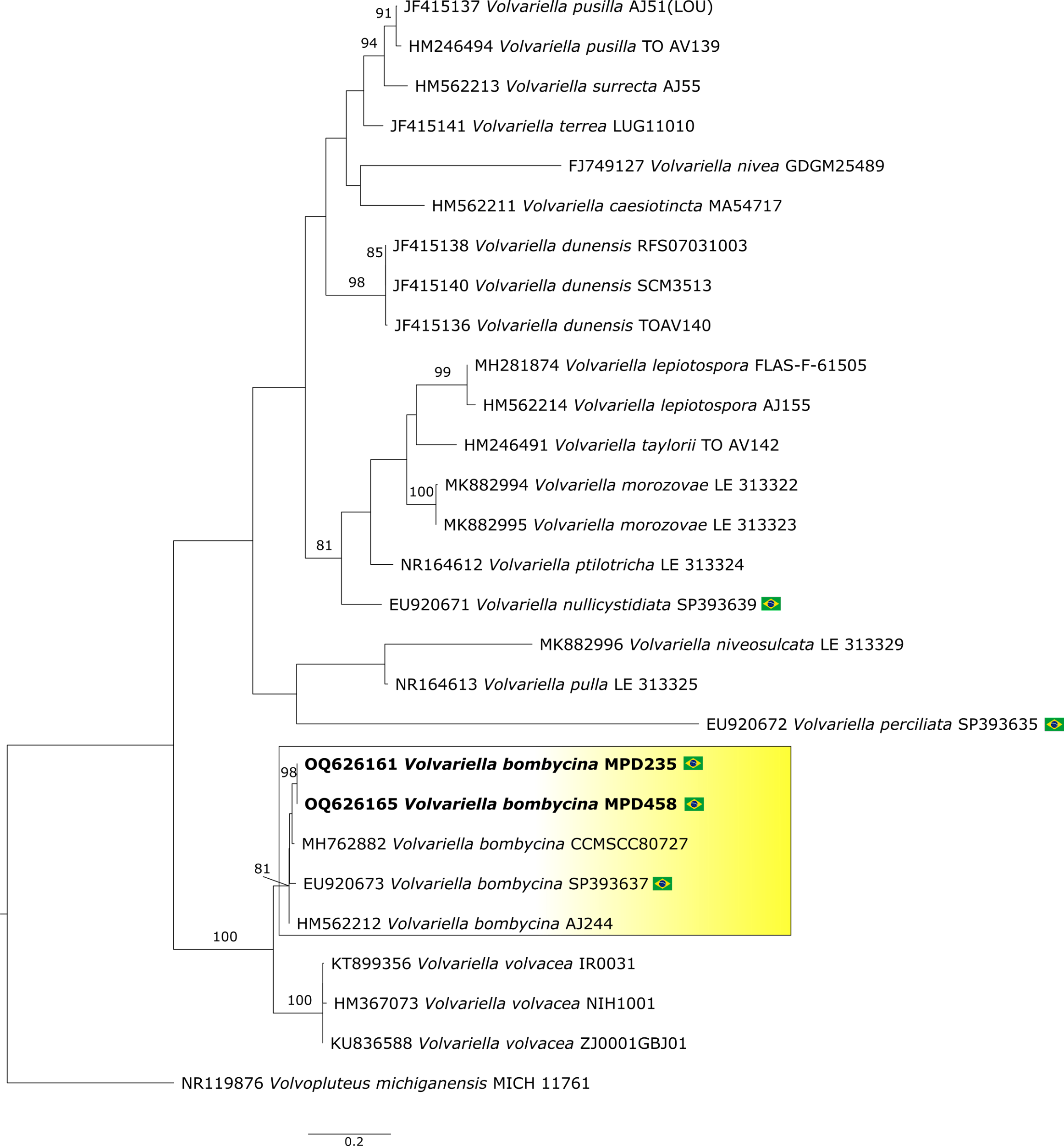


Figure S43. Maximum Likelihood (ML) tree of *Volvariella* based on ITS data. Branches are labeled with ML bootstrap higher than 80%. The highlight in yellow represents the clade of species *Volvariella bombycina*. The sequences in bold were generated in this study.
